# Supplementary material for: Histone modifications and Sp1 promote GPR160 expression in bone cancer pain within rodent models
Source: EMBO Rep. 2024 Oct 24;25(12):5429–55. doi: 10.1038/s44319-024-00292-6 (PMC11624276; doi:10.1038/s44319-024-00292-6)
Supplement: Supplementary file 1 — Appendix [file 44319_2024_292_MOESM1_ESM.pdf]

Table of Contents

Appendix Fig. S1 2

Appendix Fig. S2 4

Appendix Fig. S3 5

Appendix Table S1. Locomotor functions 6

Appendix Table S2. Primers used in this study 7

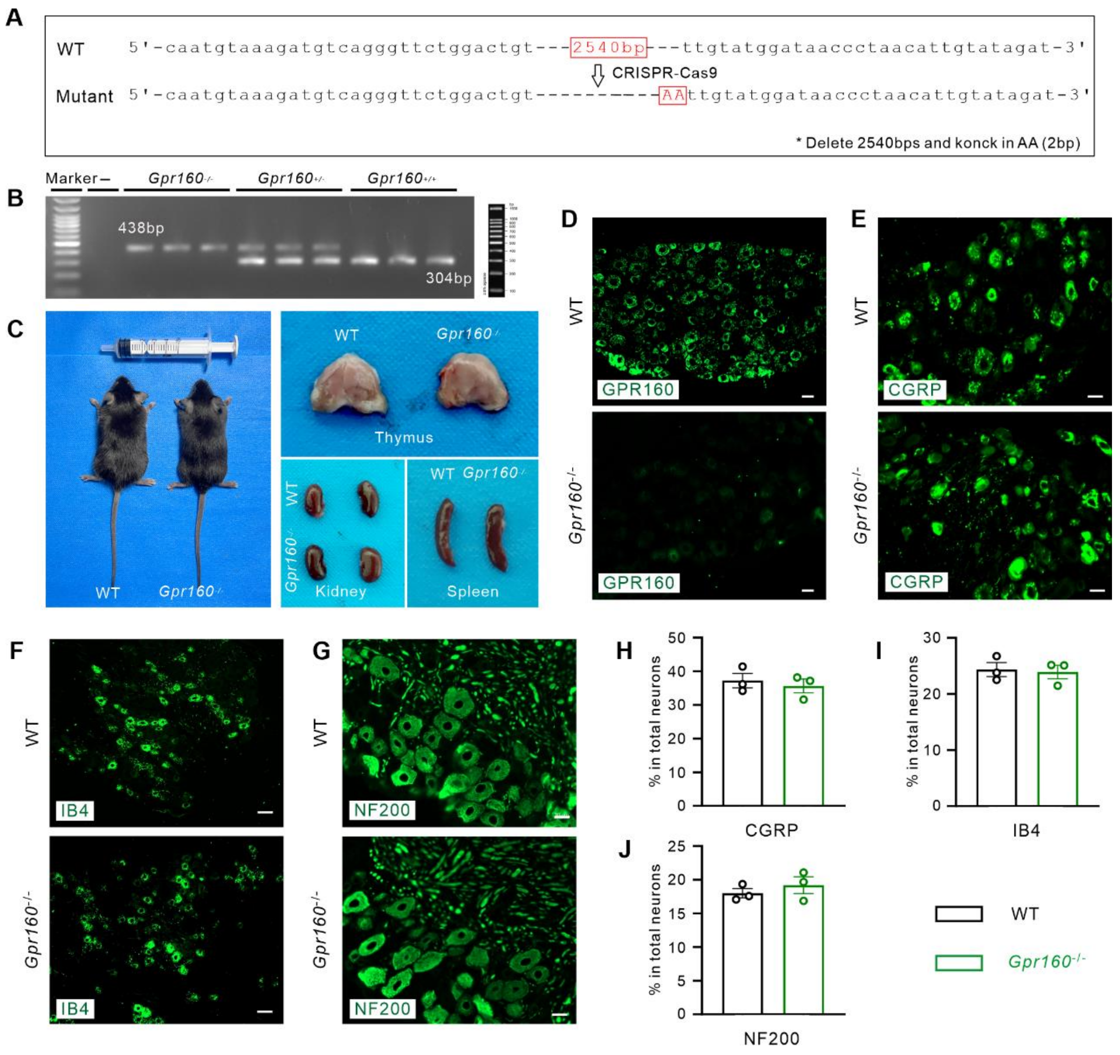

Appendix Fig. S1. Identification of *Gpr160*$^{-/-}$ mice, and the expression of cellular markers in *Gpr160*$^{-/-}$ mice.

(A) A schematic representation illustrates the methodology employed for the generation of *Gpr160*$^{-/-}$ mice through the CRISPR-Cas9 system. (B) Genotyping was performed to distinguish *Gpr160*$^{-/-}$, *Gpr160*$^{+/-}$, and *Gpr160*$^{+/+}$ (*WT*) mice. (C) Photographic documentation of both WT and *Gpr160*$^{-/-}$ mice revealed no discernible anatomical alterations, and gross morphologies of the dissected thymocytes, kidney, and spleen nodes from 8-week-old WT and *Gpr160*$^{-/-}$ mice. (D) Immunostaining clearly exhibited the absence of GPR160 signal in *Gpr160*$^{-/-}$ mice. Scale bar: 20 µm. (E-G) Analysis of CGRP, IB4, and NF200 staining in the DRG was performed in naive WT and *Gpr160*$^{-/-}$ mice. (H-J) The proportions of CGRP^{+}(P = 0.7085 versus WT group), IB4^{+}(P = 0.7373 versus WT group), and NF200^{+}(P = 0.3645 versus WT group) neurons were

quantified in the DRG of naive mice for both WT and *Gpr160*$^{-/-}$ groups. Student's unpaired t test, n = 3 mice/group.

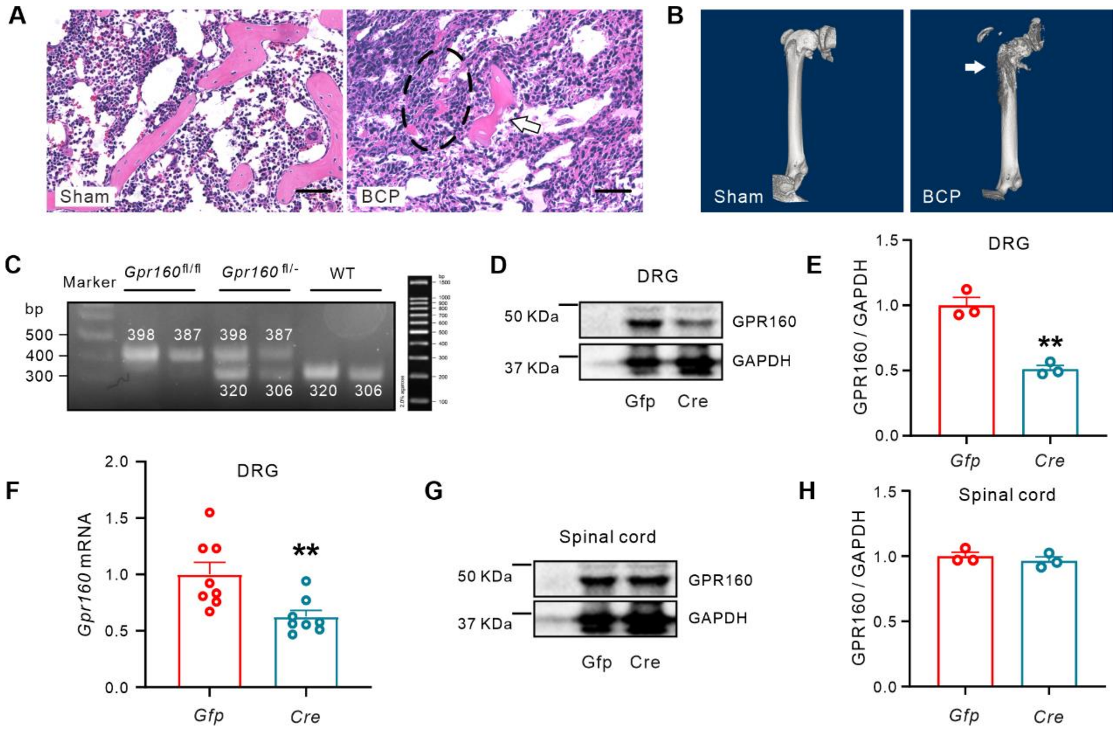

Appendix Fig. S2. Mouse BCP surgery and Generation of *Gpr160*$^{fl/fl}$ mice.

(A) Hematoxylin and eosin (H&E) staining illustrated the replacement of bone marrow by invading tumor cells, resulting in medullary bone loss and femur bone destruction on POD 18. Scale bar: 50 µm. (B) Representative micro-CT images displayed the femur bone microstructure of mice 18 days after tumor infiltration, comparing sham and BCP mice. (C) Genotyping of *Gpr160*$^{fl/fl}$ recombinase DNA. (D-E) Representative immunoblots (D) and summarized data (E) illustrating the GPR160 protein expression in the ipsilateral L3/4 DRG of *Gpr160*$^{fl/fl}$ mice microinjected with AAV-*Pirt*-Cre in the ipsilateral L3/4 DRG. Student's unpaired t test, n = 3 biological repeats (9 mice)/group, ***P* = 0.0019 versus *Gfp* group. (F) Effect of AAV-*Pirt*-Cre microinjection into the ipsilateral L3/4 DRG of *Gpr160*$^{fl/fl}$ mice on the *Gpr160* mRNA level. Student's unpaired t test, n = 8 biological repeats (16 mice)/group, ***P* = 0.0078 versus *Gfp* group. (G-H) Representative immunoblots (G) and summarized data (H) illustrating the GPR160 protein expression in spinal cord of *Gpr160*$^{fl/fl}$ mice microinjected with AAV-*Pirt*-Cre in the ipsilateral L3/4 DRG. Student's unpaired t test, n = 3 biological repeats (9 mice)/group, *P* = 0.4584 versus *Gfp* group.

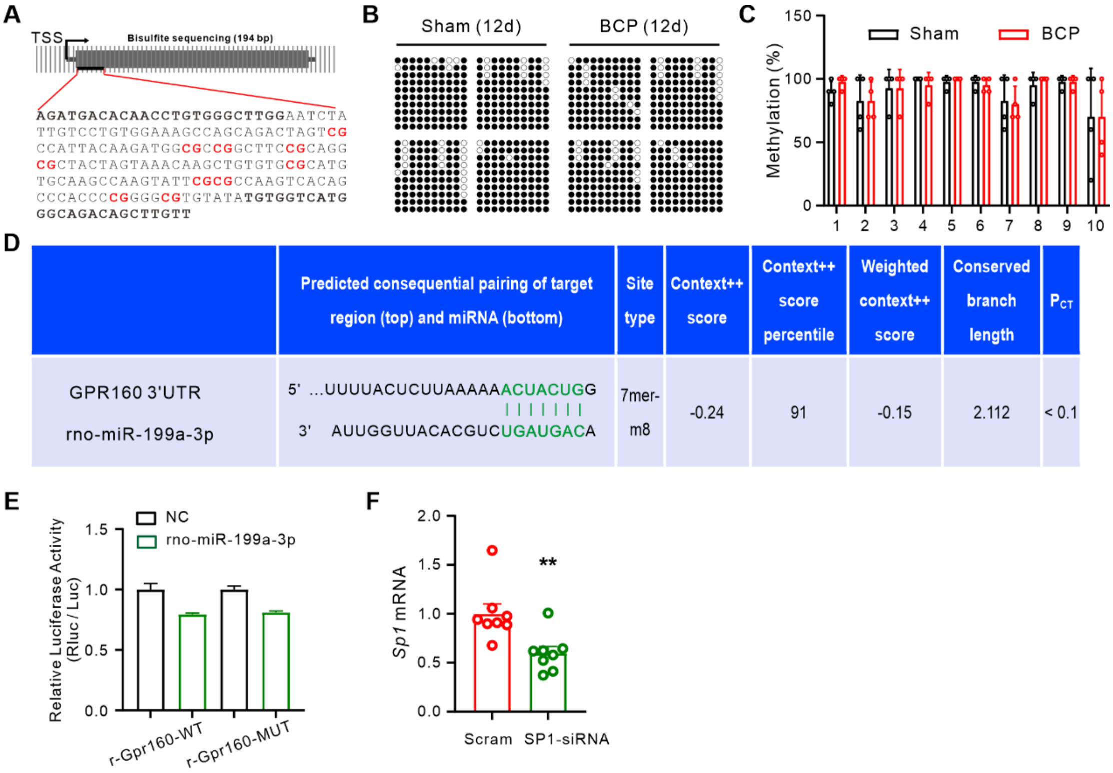

Appendix Fig. S3. The epigenetic modification of the GPR160 gene was explored. (A) The schematic representation delineates the positions of 10 CpG sites (red) situated within a CpG island within the *Gpr160* gene promoter region. (B) Bisulfite sequencing (BSP) analysis of the *Gpr160* promoter region in the DRG of rats subjected to either sham or BCP procedures. Ten clones were randomly sampled from each rat. Methylated CpG sites are denoted by filled circles, while unmethylated CpG sites are represented by unfilled circles. (C) Analysis of the overall methylation status of the *Gpr160* promoter did not reveal a statistically significant difference following the induction of BCP. Two-way ANOVA with repeated measures followed by the post hoc Tukey test. (D) Prediction of binding sites between *Gpr160* and miR-199a-3p was performed utilizing bioinformatics analyses to anticipate miRNA-mediated targeting of *Gpr160*. (E) The dual-luciferase reporter assay for evaluating the interaction between *Gpr160* and miR-199a-3p. (F) RT-PCR validation confirmed a reduction in *Sp1* mRNA levels in the DRGs following knockdown on POD 14. Student's unpaired t test, n = 8 rats/group, $^{**}P = 0.0051$ versus Scram group.

Appendix Table S1: Locomotor functions

Treatments/genotyping	Functional test		
	Placing	Grasping	Righting
Gpr160 siRNA + Sham	5 (0)	5 (0)	5 (0)
Gpr160 siRNA + BCP	5 (0)	5 (0)	5 (0)
Gpr160 scramble + Sham	5 (0)	5 (0)	5 (0)
Gpr160 scramble + BCP	5 (0)	5 (0)	5 (0)
AAV2/9-*Gpr160*-shRNA + Sham	5 (0)	5 (0)	5 (0)
AAV2/9-*Gpr160*-shRNA + BCP	5 (0)	5 (0)	5 (0)
AAV2/9-Scramble-shRNA + Sham	5 (0)	5 (0)	5 (0)
AAV2/9-Scramble-shRNA + BCP	5 (0)	5 (0)	5 (0)
AAV2/9-*Gfp*	5 (0)	5 (0)	5 (0)
AAV2/9-*Gpr160*	5 (0)	5 (0)	5 (0)
PBS	5 (0)	5 (0)	5 (0)
LV-*Gfp*	5 (0)	5 (0)	5 (0)
LV-*Gpr160*	5 (0)	5 (0)	5 (0)
Gpr160$^{fl/fl}$ + AAV2/9-*Prit*-cre + Sham	5 (0)	5 (0)	5 (0)
Gpr160$^{fl/fl}$ + AAV2/9-*Prit*-cre + BCP	5 (0)	5 (0)	5 (0)
Gpr160$^{fl/fl}$ + AAV2/9-*Gfp* + Sham	5 (0)	5 (0)	5 (0)
Gpr160$^{fl/fl}$ + AAV2/9-*Gfp* + BCP	5 (0)	5 (0)	5 (0)

Appendix Table S2: Primers used in this study

Gene	Sequences of PCR primers
Gpr160-F	TTCCTTCGCTTACGGCTTCTTGC
Gpr160-R	GCTTGGTGGCTCTGGACAGATTAC
Sp1-F	GGCAGACTAGCAGCAGCAATACC
Sp1-R	ATGGAGGACAGTTGAGCAGCATTC
βactin-F	ATCACTATCGGCAATGAGCGGTTC
βactin-R	TGTTGGCATAGAGGTCTTTACGGATG
Gapdh-F	ACAGCAACAGGGTGGTGGAC
Gapdh-R	TTTGAGGGTGCAGCGAACTT
Gpr160$^{fl/fl}$-F1	AATTGGTAATTGCCCTGAGCATC
Gpr160$^{fl/fl}$-R1	GGCATAGTCTCACAAGAGACAGCTAT
Gpr160$^{fl/fl}$-F2	TTAATGGCGTGTGCCACCAG
Gpr160$^{fl/fl}$-R2	TGTGGTTAGGAGTTGTGCCCA
Gpr160$^{-/-}$-F1	TGAGCATCACTATCCAGCCTGC
Gpr160$^{-/-}$-R1	GCTAGTGGTGGTTCTTGAAACACG
Gpr160$^{-/-}$-F2	GATTAAGGGCTGGATTATCCTGTC
Gpr160$^{-/-}$-R2	GCTAGTGGTGGTTCTTGAAACACG
GPR160 ChIP-F	GGAGGTTGGTGGCTTCAGA
GPR160 ChIP -R	GAAGGGAAGCCTCGTCGT
