# Supplementary figures and images for: Histone modifications and Sp1 promote GPR160 expression in bone cancer pain within rodent models (part 1 of 2)
Source: EMBO Rep. 2024 Oct 24;25(12):5429–55. doi: 10.1038/s44319-024-00292-6 (PMC11624276; doi:10.1038/s44319-024-00292-6)

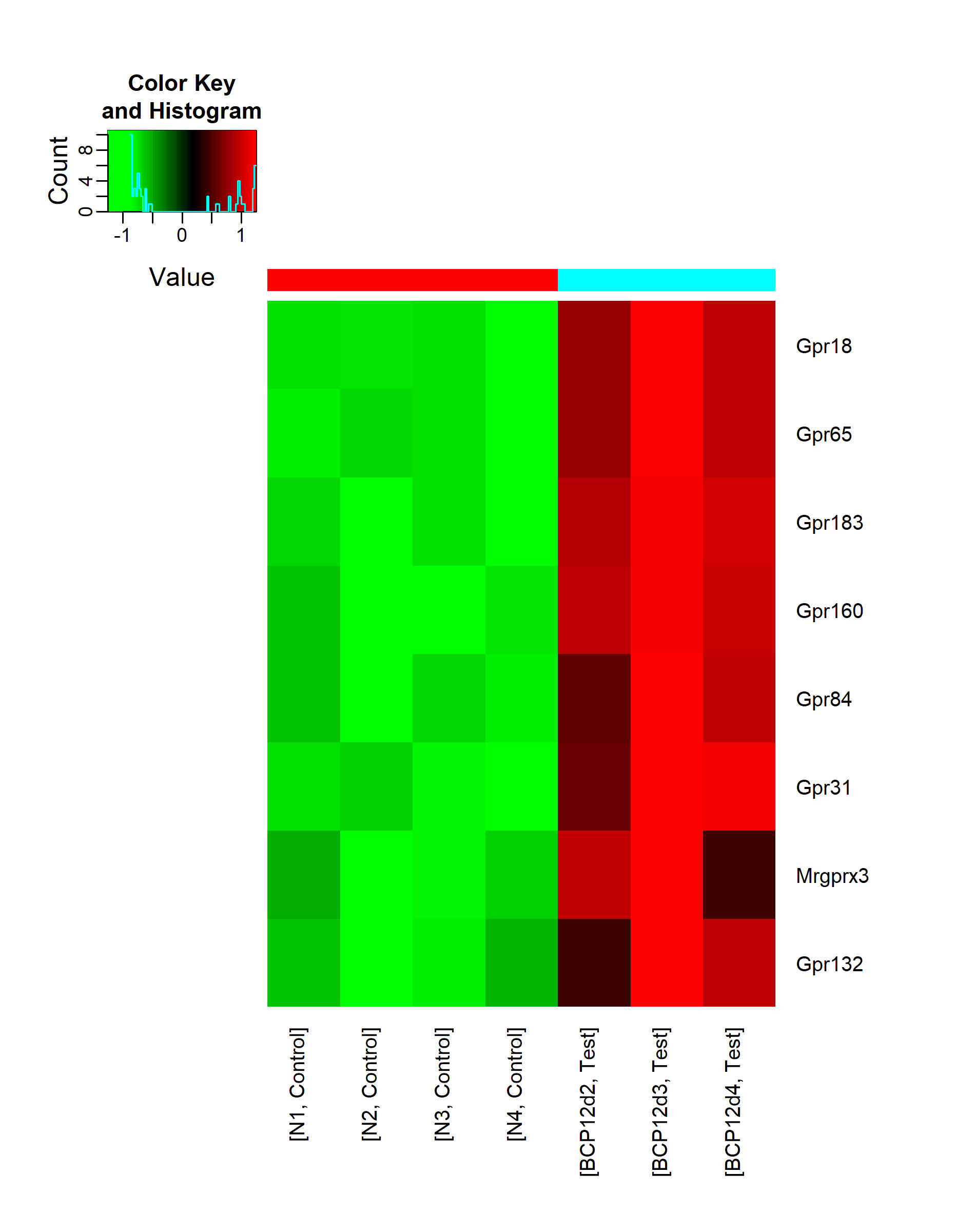

Supplement: Supplementary file 3 — Source data Fig. 1 [file 44319_2024_292_MOESM3_ESM.zip › EMBOR-2024-59294V3-Figure_1_Source_Data-sd/embr202459294-sup-sdatafig1/1A/1A.tif]

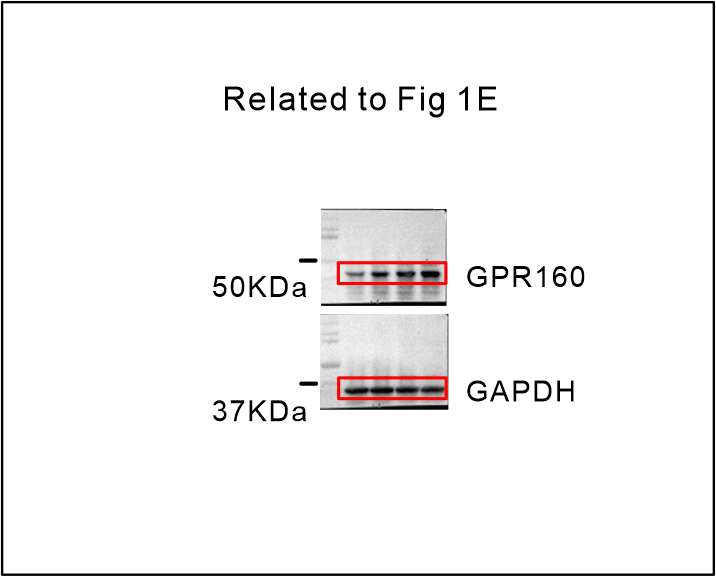

Supplement: Supplementary file 3 — Source data Fig. 1 [file 44319_2024_292_MOESM3_ESM.zip › EMBOR-2024-59294V3-Figure_1_Source_Data-sd/embr202459294-sup-sdatafig1/1E/1E.tif]

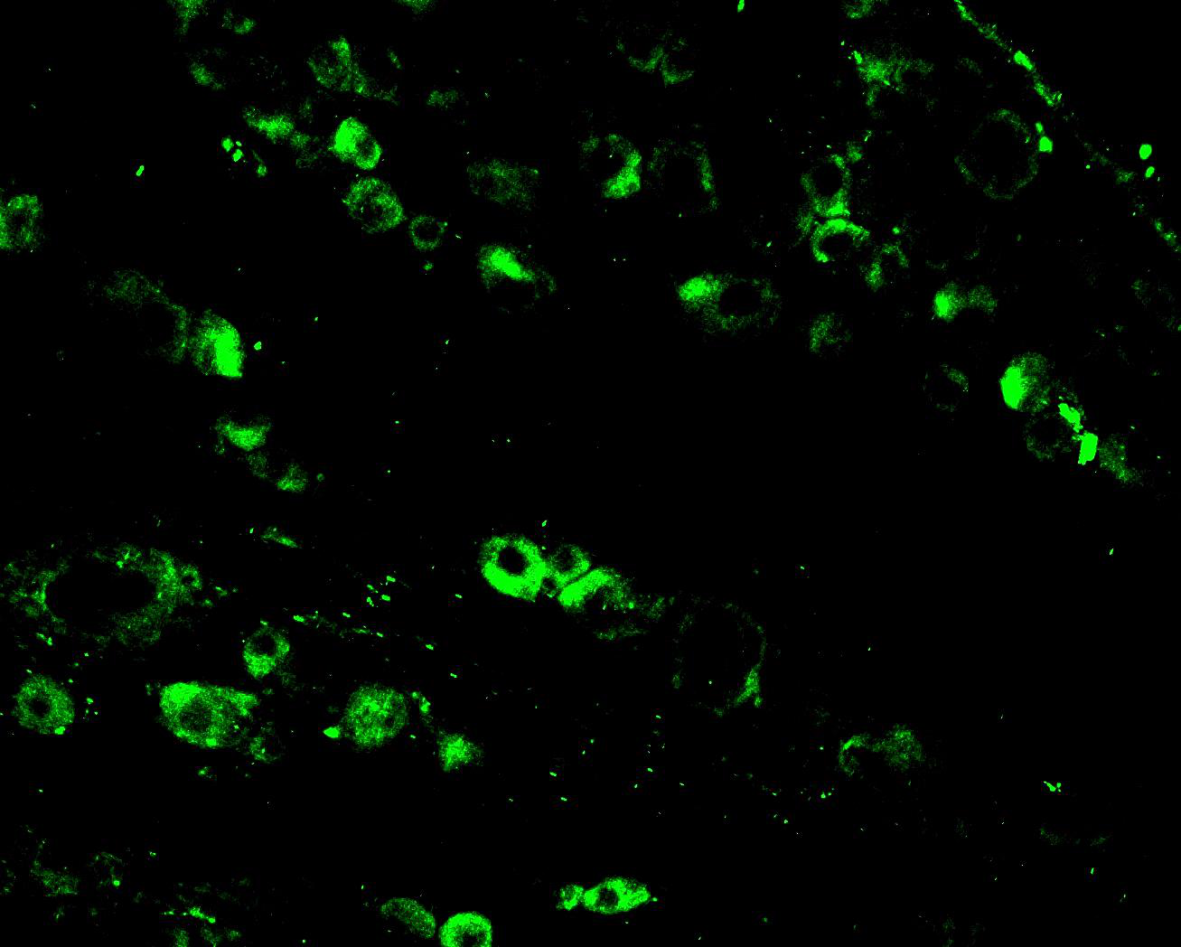

Supplement: Supplementary file 3 — Source data Fig. 1 [file 44319_2024_292_MOESM3_ESM.zip › EMBOR-2024-59294V3-Figure_1_Source_Data-sd/embr202459294-sup-sdatafig1/1G-J/1G.tif]

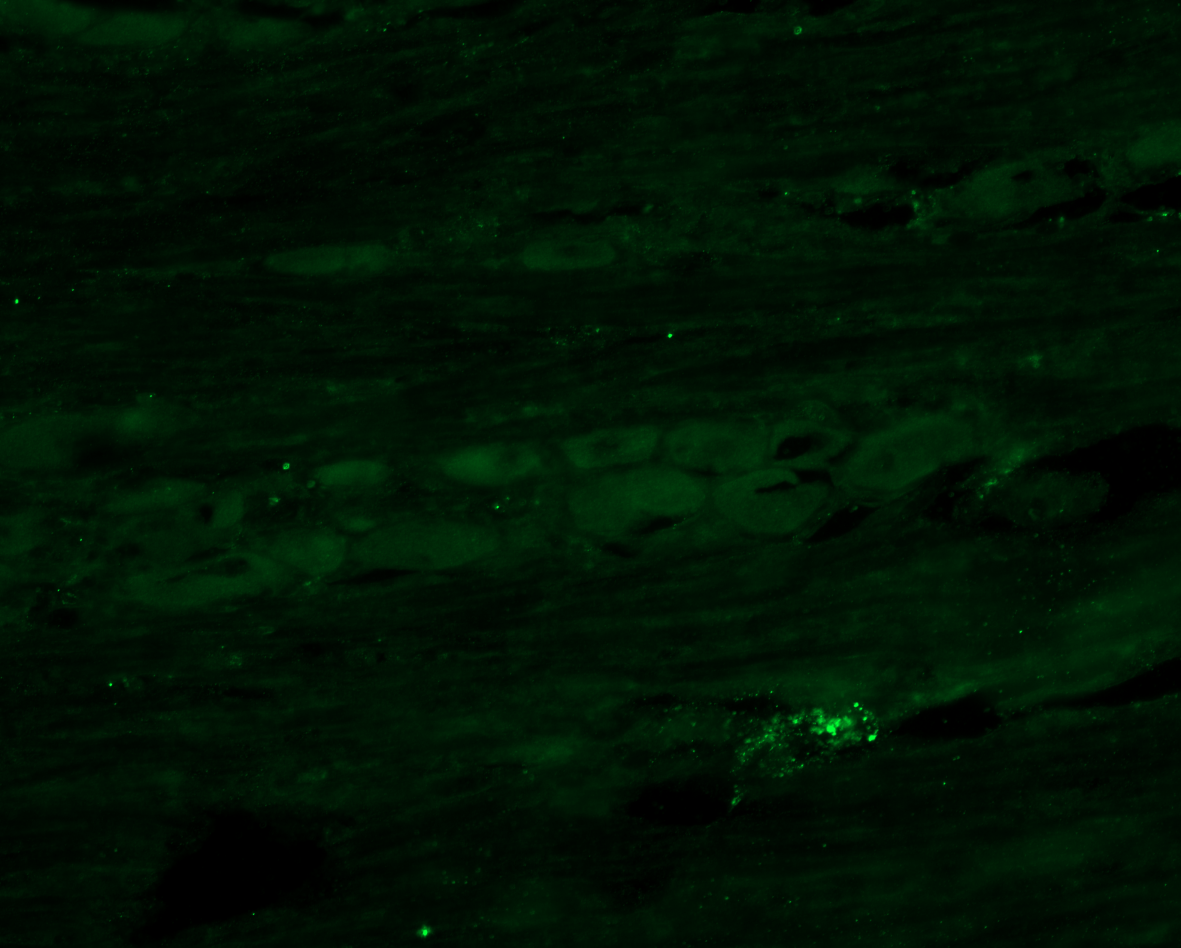

Supplement: Supplementary file 3 — Source data Fig. 1 [file 44319_2024_292_MOESM3_ESM.zip › EMBOR-2024-59294V3-Figure_1_Source_Data-sd/embr202459294-sup-sdatafig1/1G-J/1H.tif]

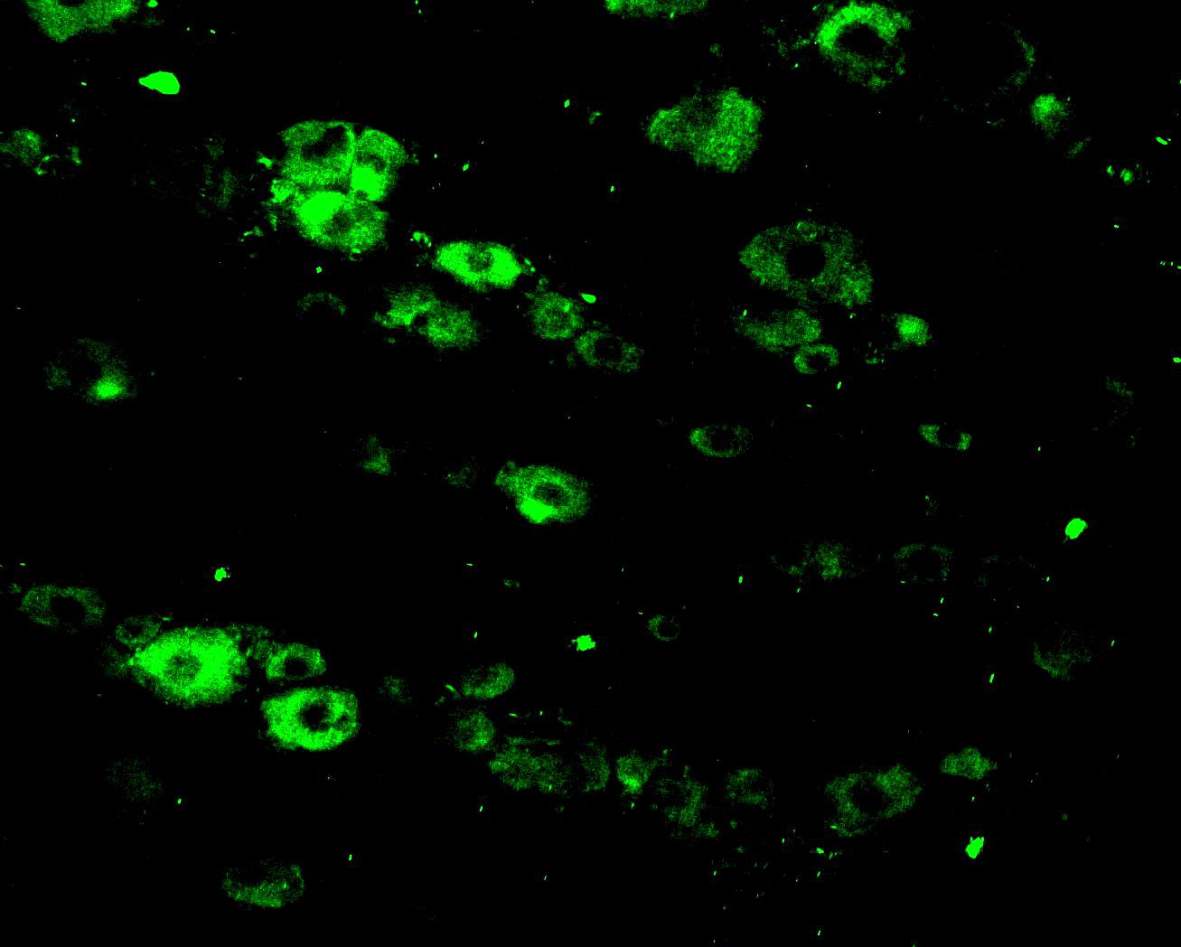

Supplement: Supplementary file 3 — Source data Fig. 1 [file 44319_2024_292_MOESM3_ESM.zip › EMBOR-2024-59294V3-Figure_1_Source_Data-sd/embr202459294-sup-sdatafig1/1G-J/1I.tif]

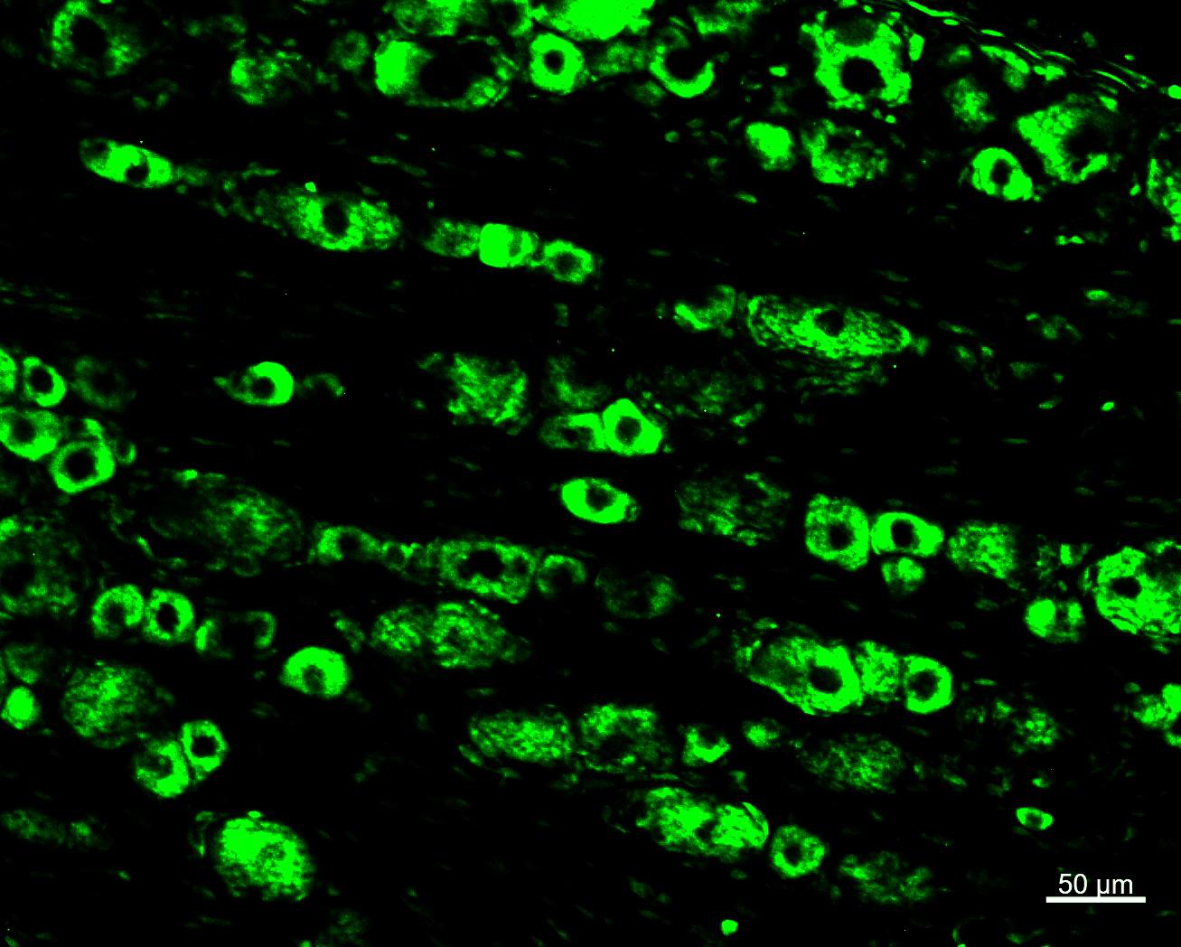

Supplement: Supplementary file 3 — Source data Fig. 1 [file 44319_2024_292_MOESM3_ESM.zip › EMBOR-2024-59294V3-Figure_1_Source_Data-sd/embr202459294-sup-sdatafig1/1G-J/1J.tif]

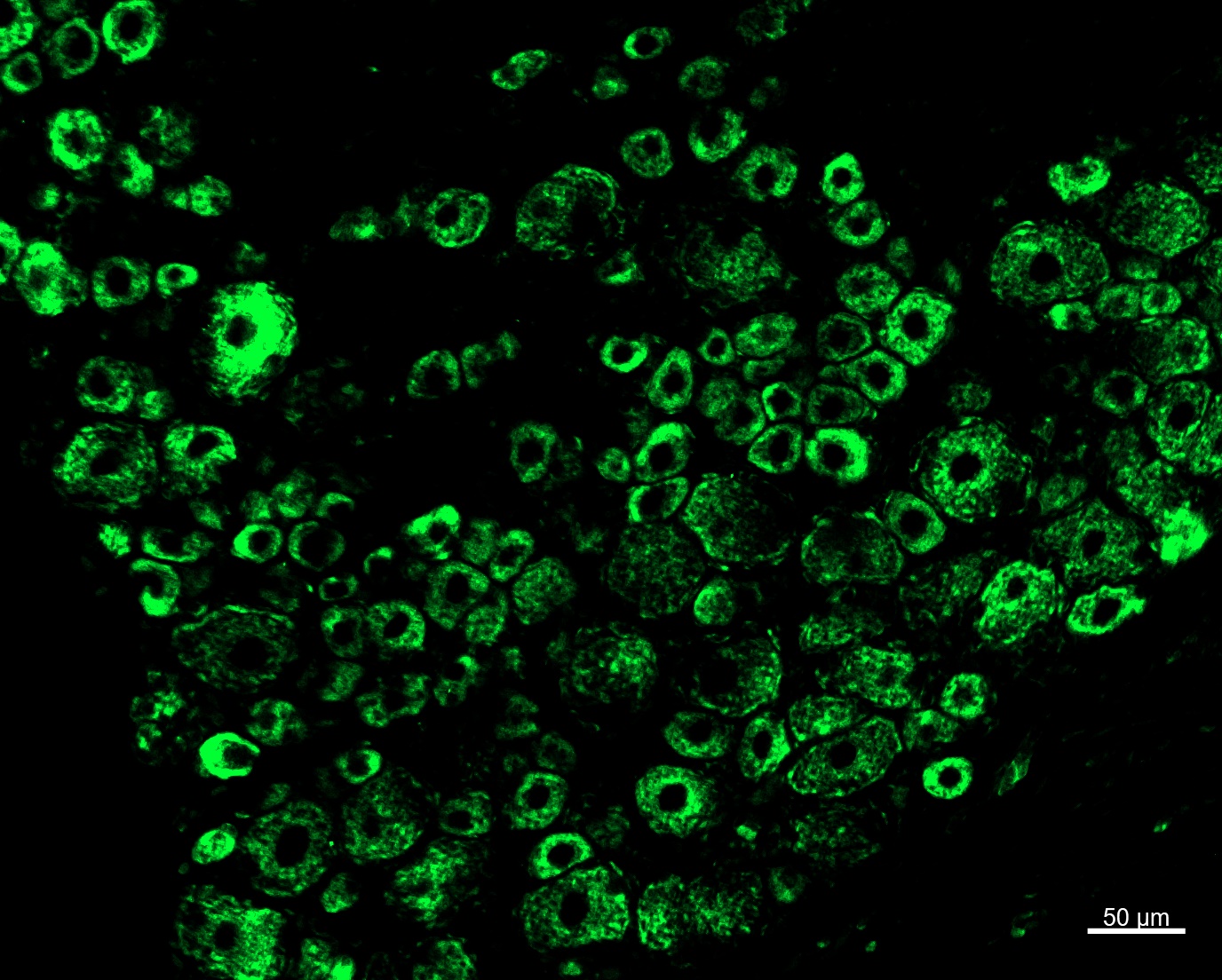

Supplement: Supplementary file 3 — Source data Fig. 1 [file 44319_2024_292_MOESM3_ESM.zip › EMBOR-2024-59294V3-Figure_1_Source_Data-sd/embr202459294-sup-sdatafig1/1K-O/1K.jpg]

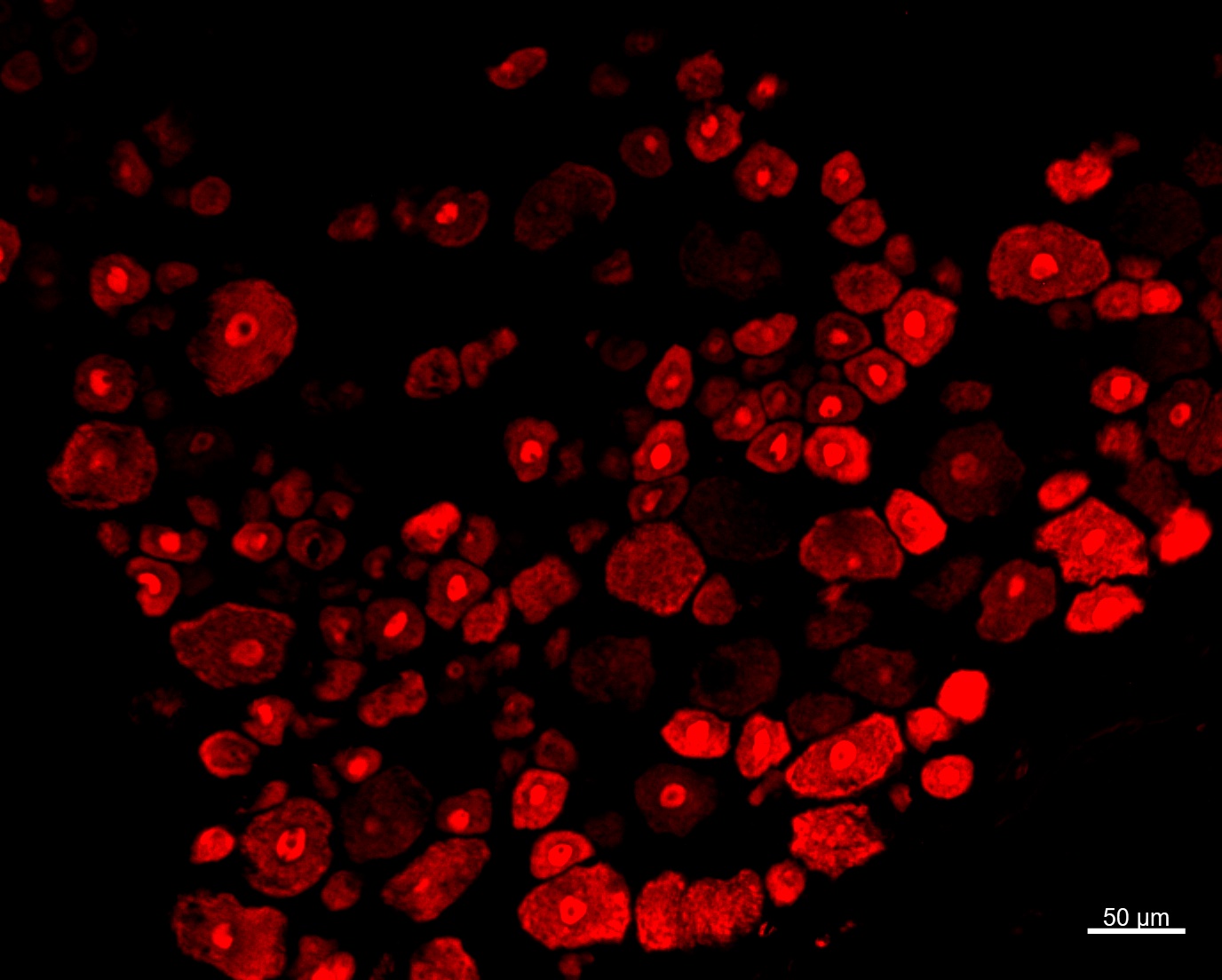

Supplement: Supplementary file 3 — Source data Fig. 1 [file 44319_2024_292_MOESM3_ESM.zip › EMBOR-2024-59294V3-Figure_1_Source_Data-sd/embr202459294-sup-sdatafig1/1K-O/1L.jpg]

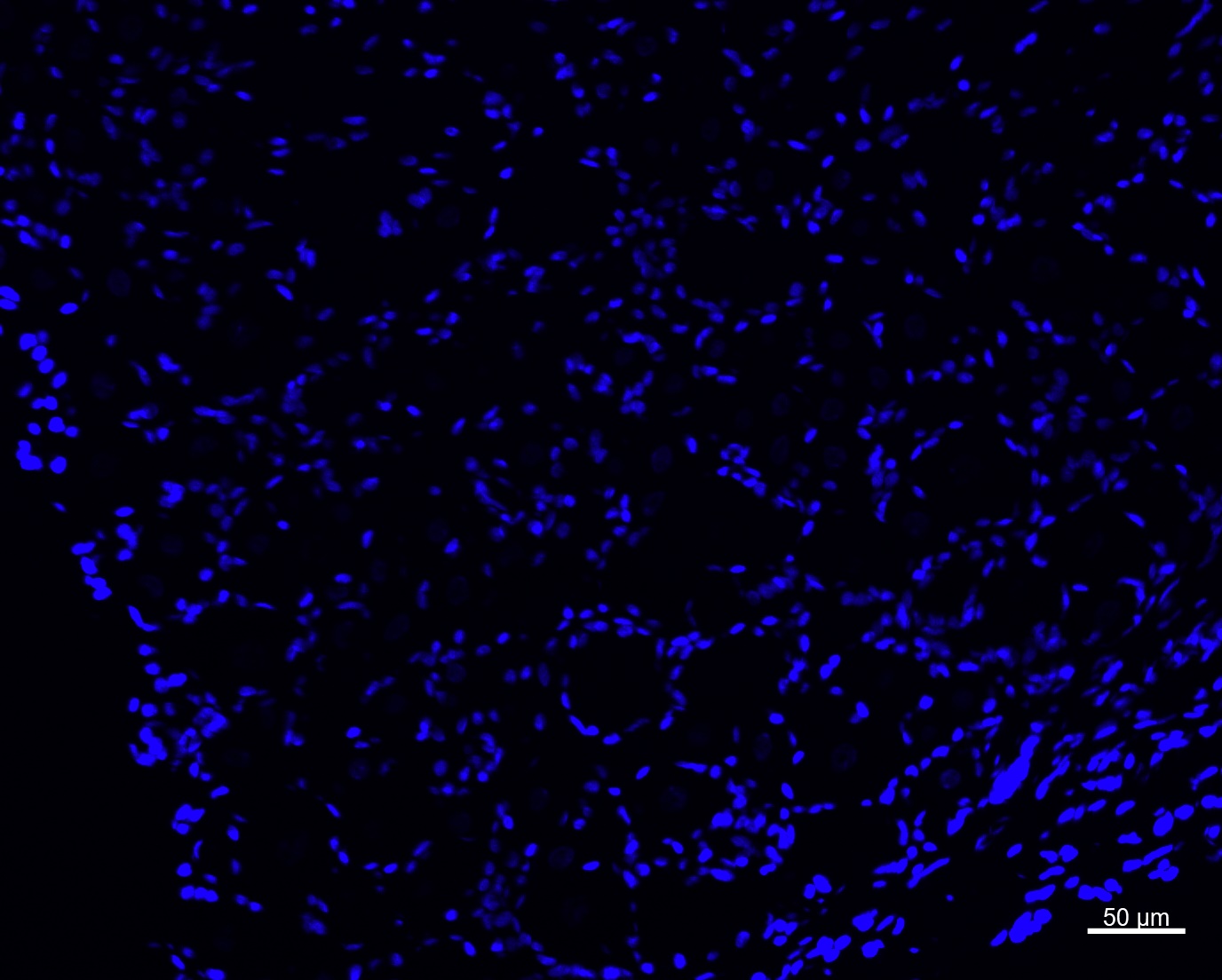

Supplement: Supplementary file 3 — Source data Fig. 1 [file 44319_2024_292_MOESM3_ESM.zip › EMBOR-2024-59294V3-Figure_1_Source_Data-sd/embr202459294-sup-sdatafig1/1K-O/1M.jpg]

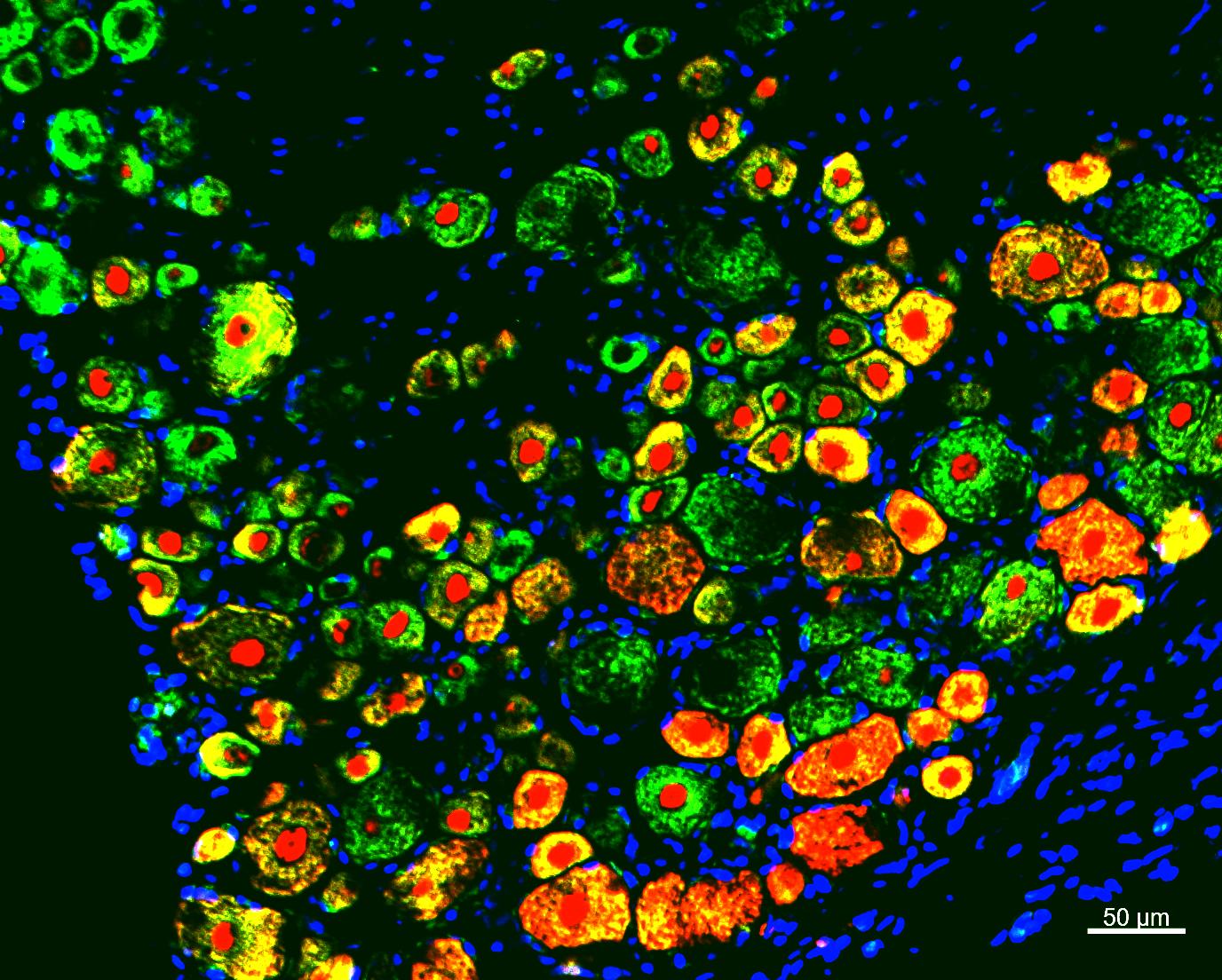

Supplement: Supplementary file 3 — Source data Fig. 1 [file 44319_2024_292_MOESM3_ESM.zip › EMBOR-2024-59294V3-Figure_1_Source_Data-sd/embr202459294-sup-sdatafig1/1K-O/1N.jpg]

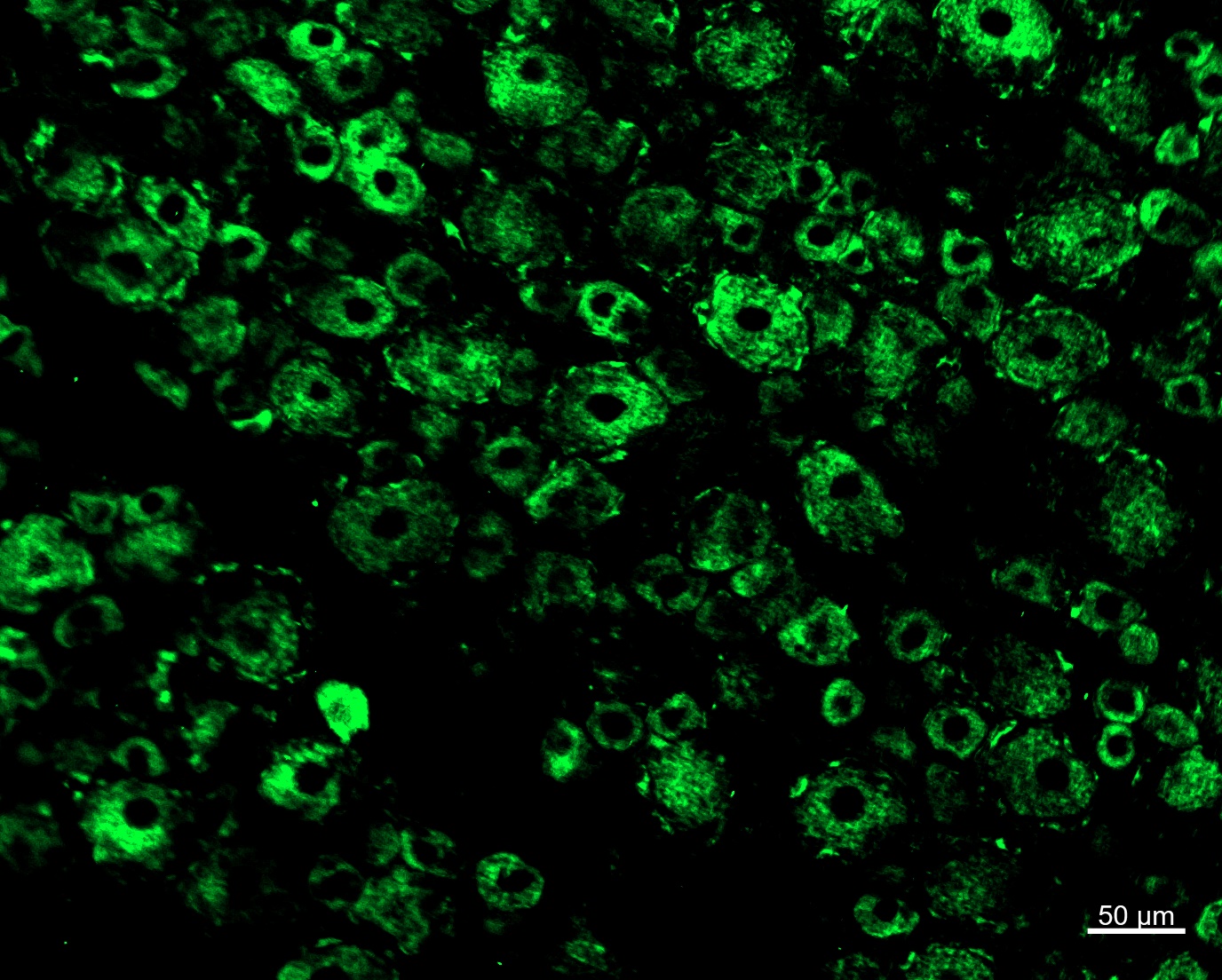

Supplement: Supplementary file 3 — Source data Fig. 1 [file 44319_2024_292_MOESM3_ESM.zip › EMBOR-2024-59294V3-Figure_1_Source_Data-sd/embr202459294-sup-sdatafig1/1P-T/1P.jpg]

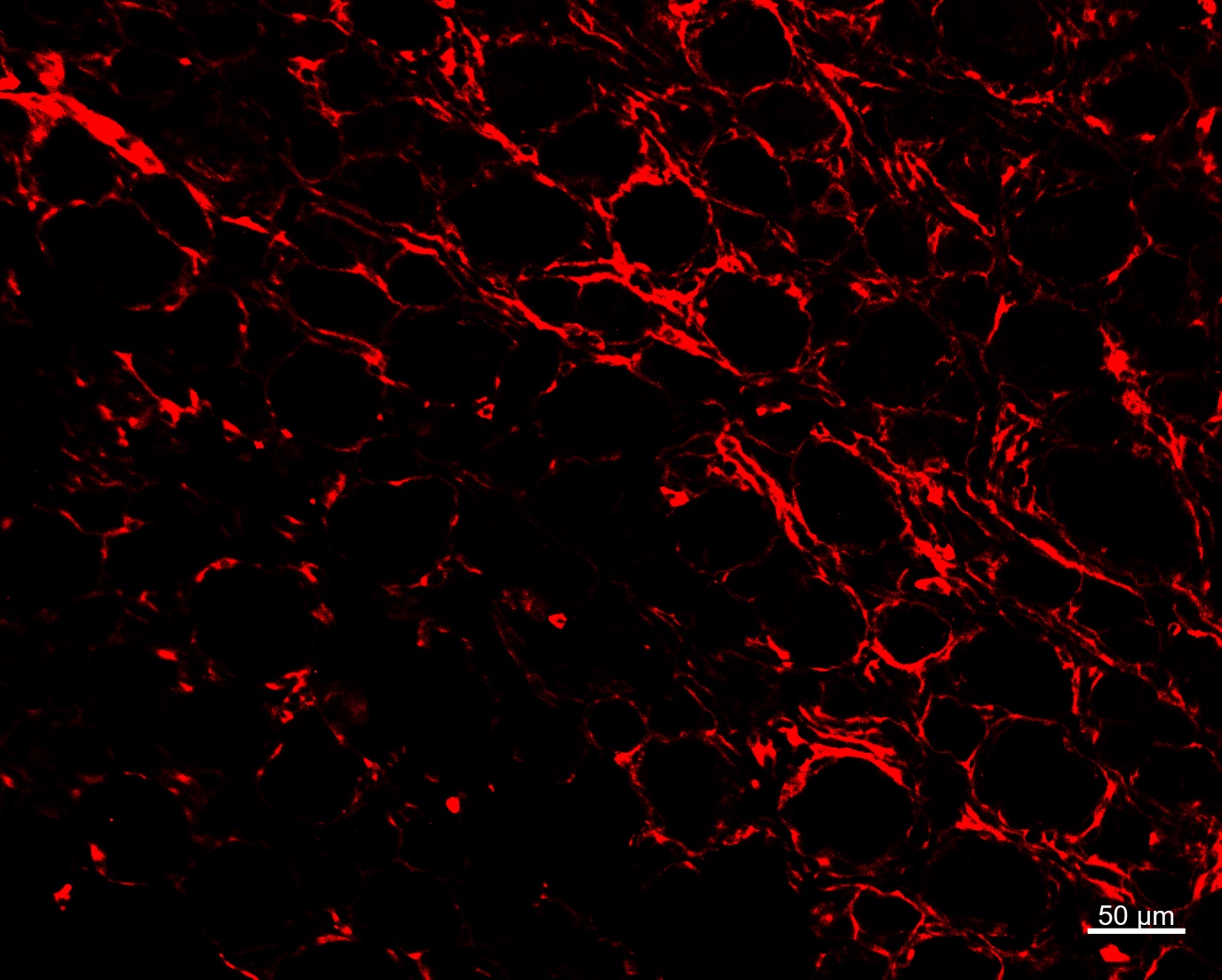

Supplement: Supplementary file 3 — Source data Fig. 1 [file 44319_2024_292_MOESM3_ESM.zip › EMBOR-2024-59294V3-Figure_1_Source_Data-sd/embr202459294-sup-sdatafig1/1P-T/1Q.jpg]

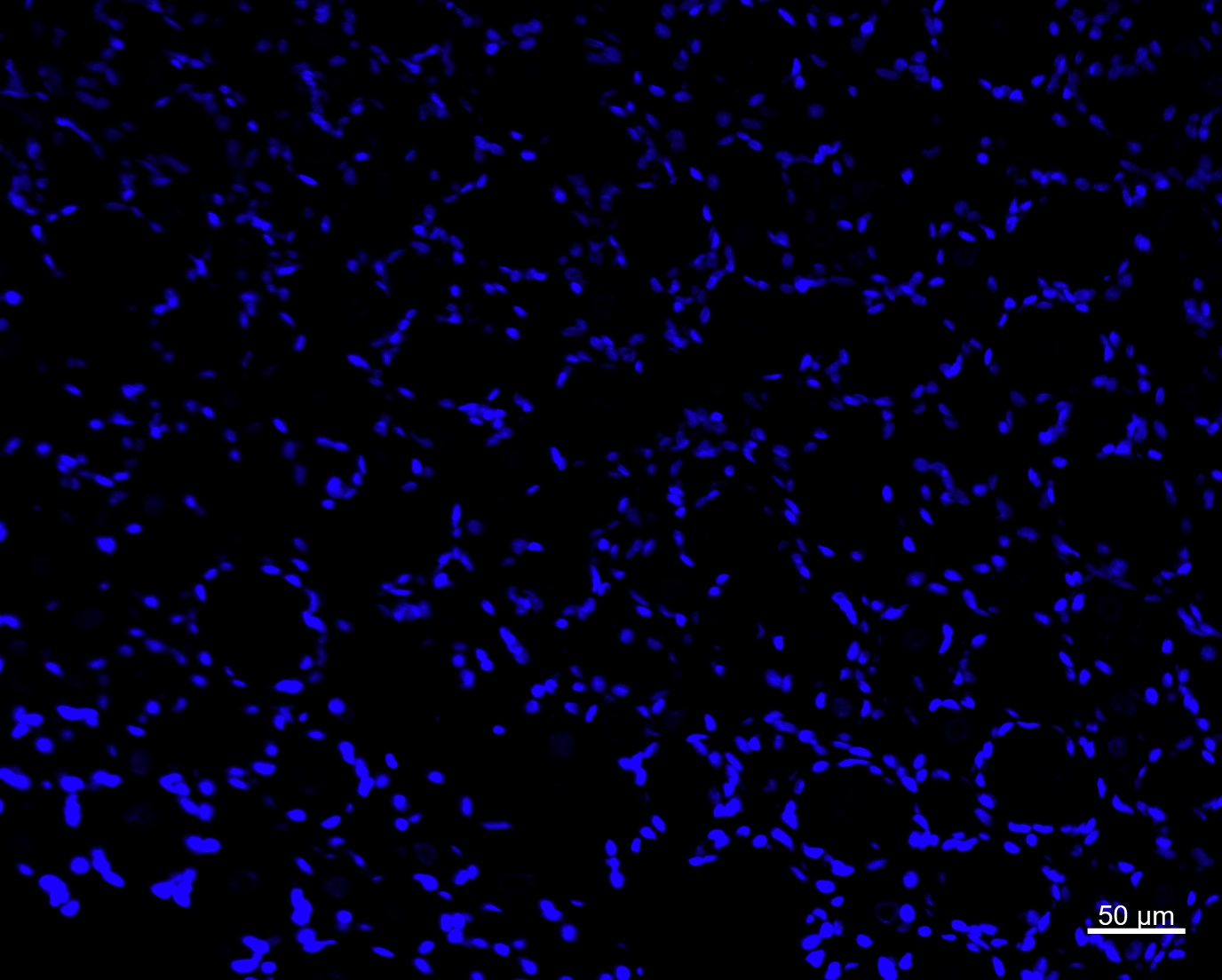

Supplement: Supplementary file 3 — Source data Fig. 1 [file 44319_2024_292_MOESM3_ESM.zip › EMBOR-2024-59294V3-Figure_1_Source_Data-sd/embr202459294-sup-sdatafig1/1P-T/1R.jpg]

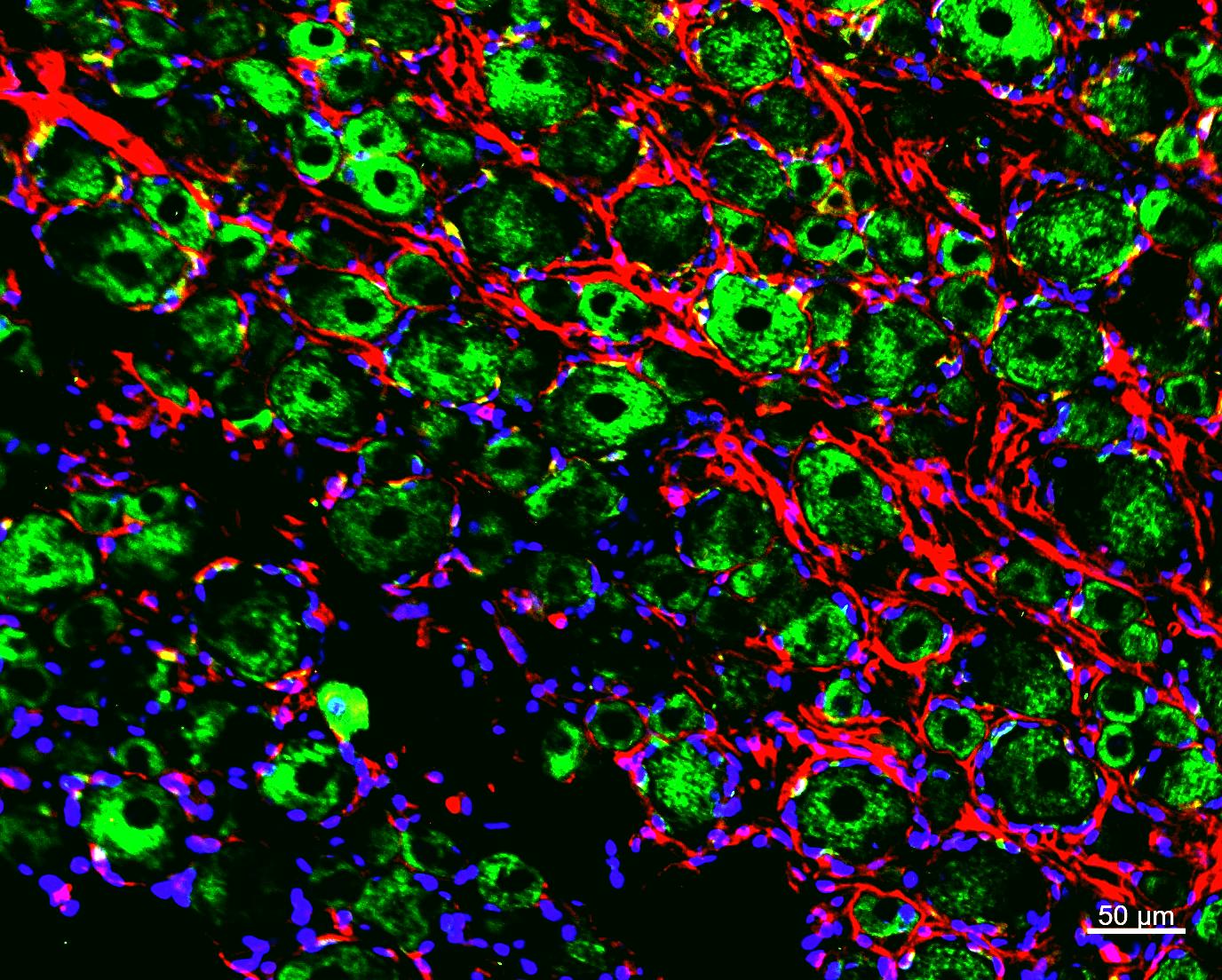

Supplement: Supplementary file 3 — Source data Fig. 1 [file 44319_2024_292_MOESM3_ESM.zip › EMBOR-2024-59294V3-Figure_1_Source_Data-sd/embr202459294-sup-sdatafig1/1P-T/1S.jpg]

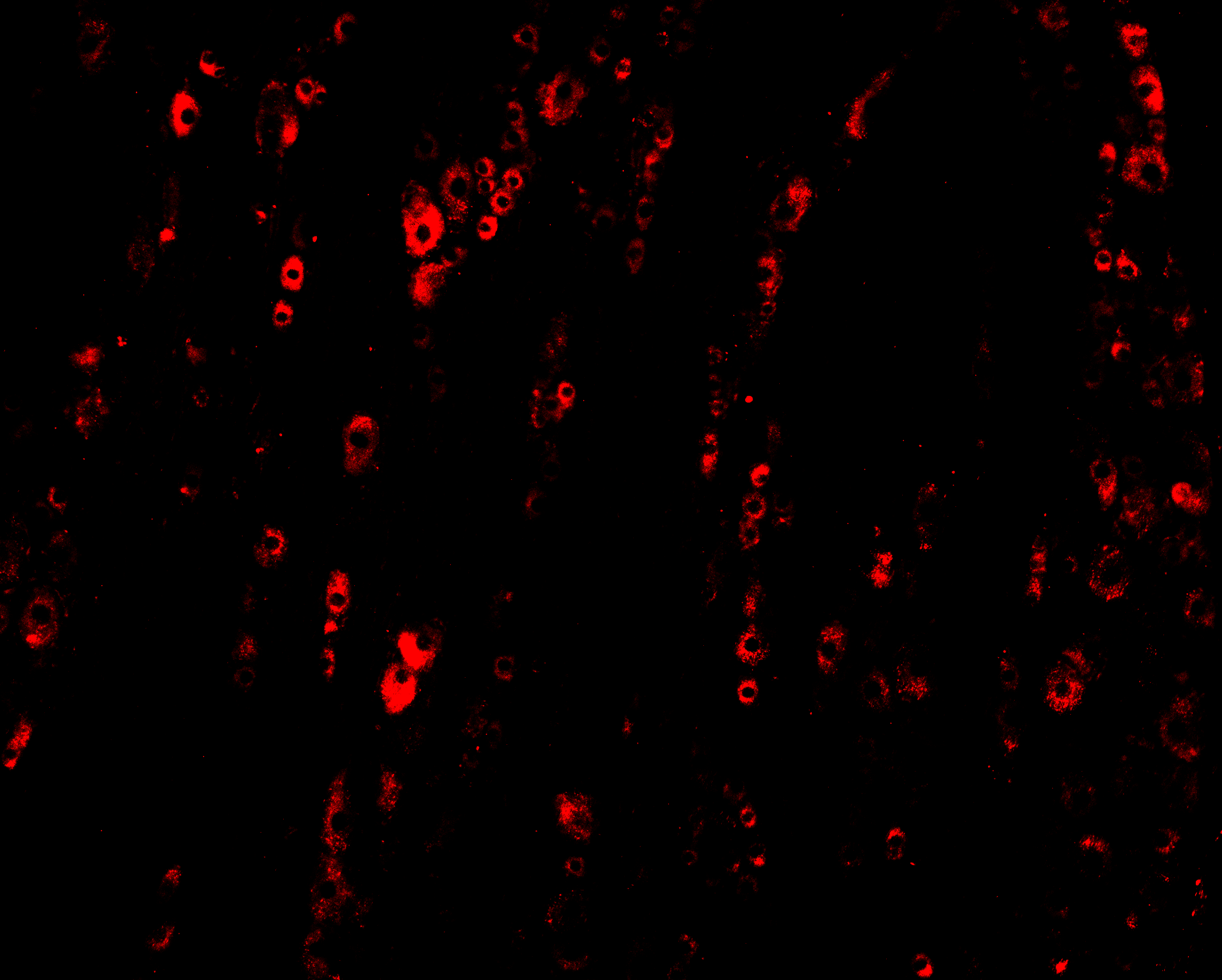

Supplement: Supplementary file 4 — Source data Fig. 2 [file 44319_2024_292_MOESM4_ESM.zip › EMBOR-2024-59294V3-Figure_2_Source_Data-sd/embr202459294-sup-sdatafig2/2A-E/2A.tif]

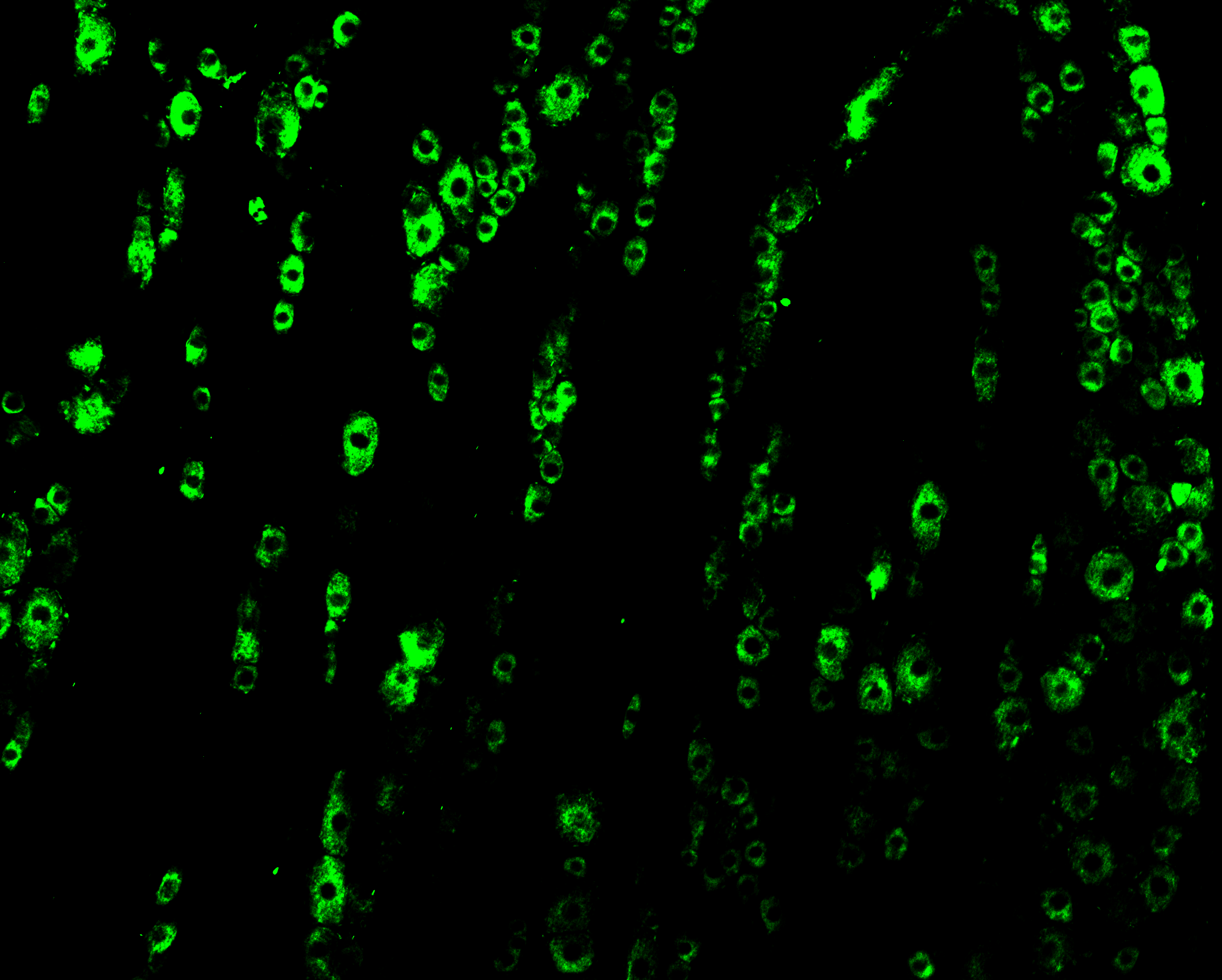

Supplement: Supplementary file 4 — Source data Fig. 2 [file 44319_2024_292_MOESM4_ESM.zip › EMBOR-2024-59294V3-Figure_2_Source_Data-sd/embr202459294-sup-sdatafig2/2A-E/2B.tif]

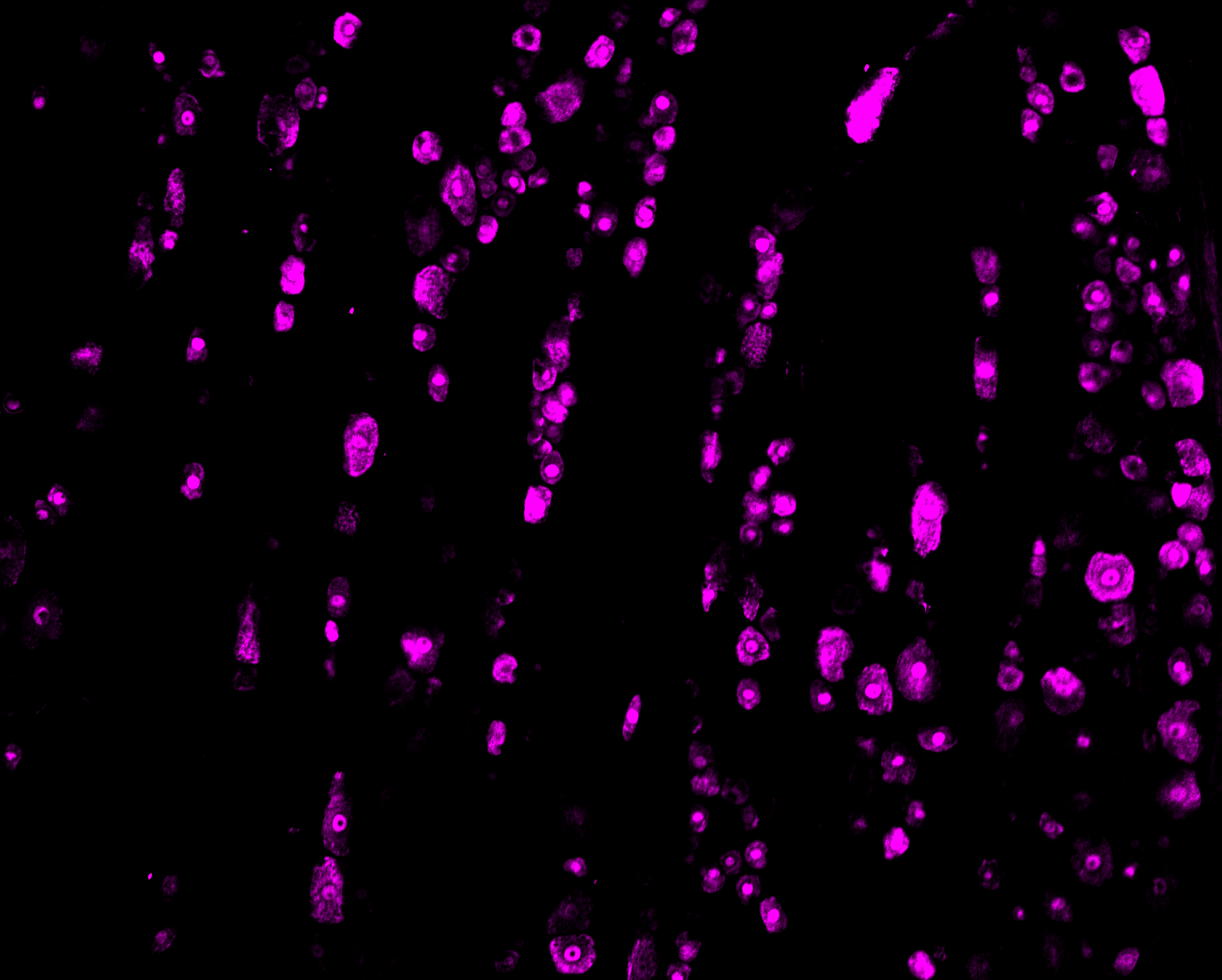

Supplement: Supplementary file 4 — Source data Fig. 2 [file 44319_2024_292_MOESM4_ESM.zip › EMBOR-2024-59294V3-Figure_2_Source_Data-sd/embr202459294-sup-sdatafig2/2A-E/2C.tif]

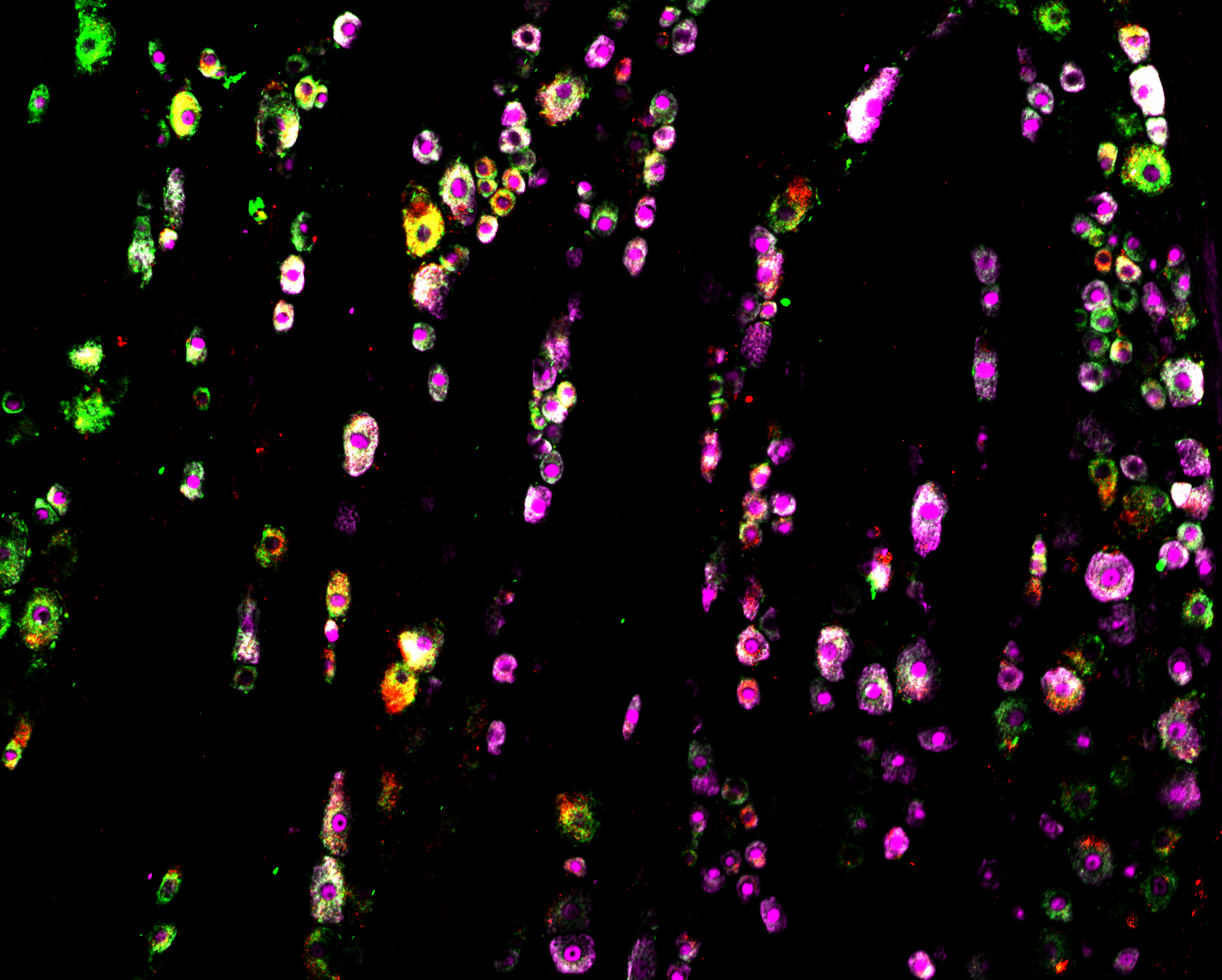

Supplement: Supplementary file 4 — Source data Fig. 2 [file 44319_2024_292_MOESM4_ESM.zip › EMBOR-2024-59294V3-Figure_2_Source_Data-sd/embr202459294-sup-sdatafig2/2A-E/2D.tif]

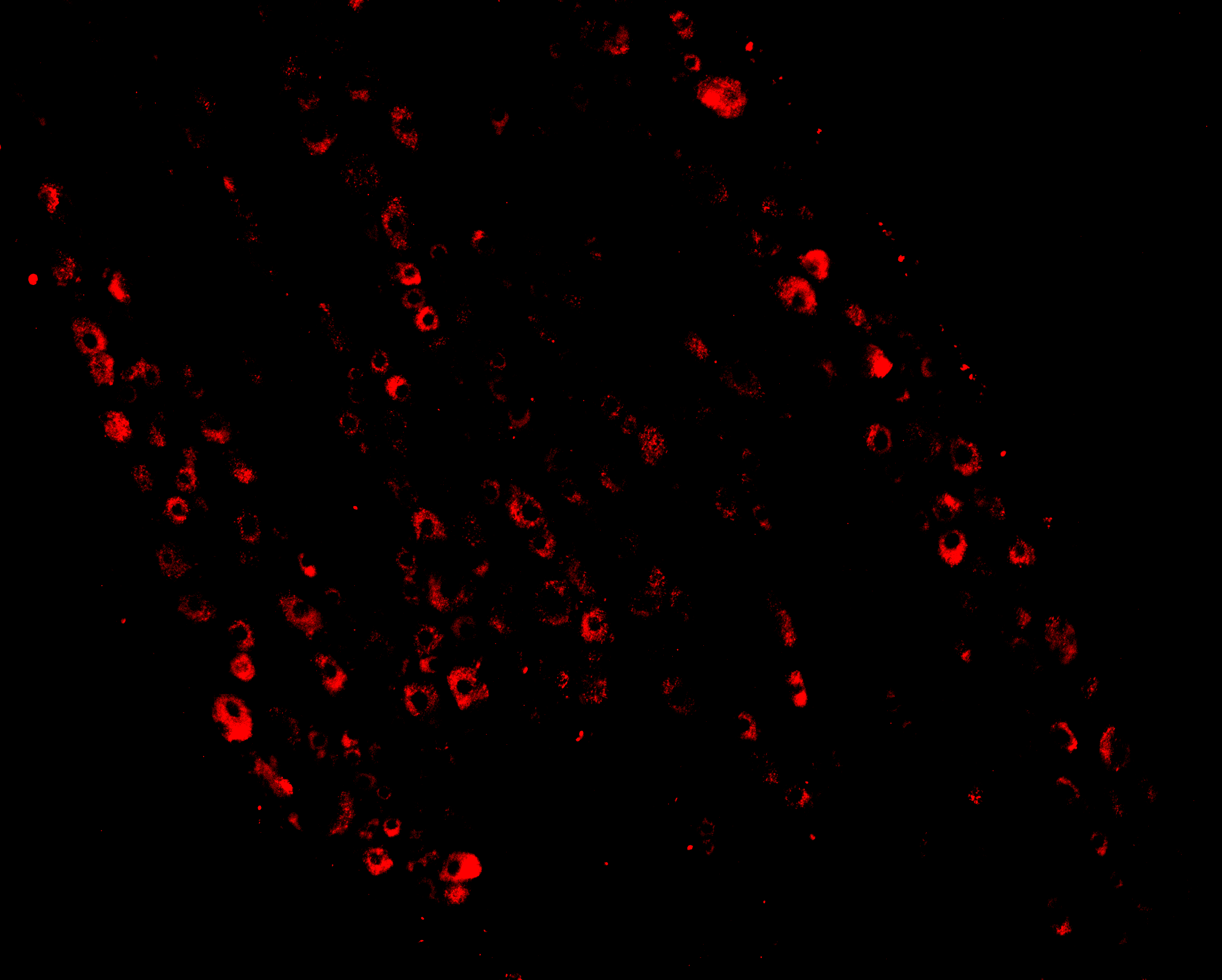

Supplement: Supplementary file 4 — Source data Fig. 2 [file 44319_2024_292_MOESM4_ESM.zip › EMBOR-2024-59294V3-Figure_2_Source_Data-sd/embr202459294-sup-sdatafig2/2F-J/2F.tif]

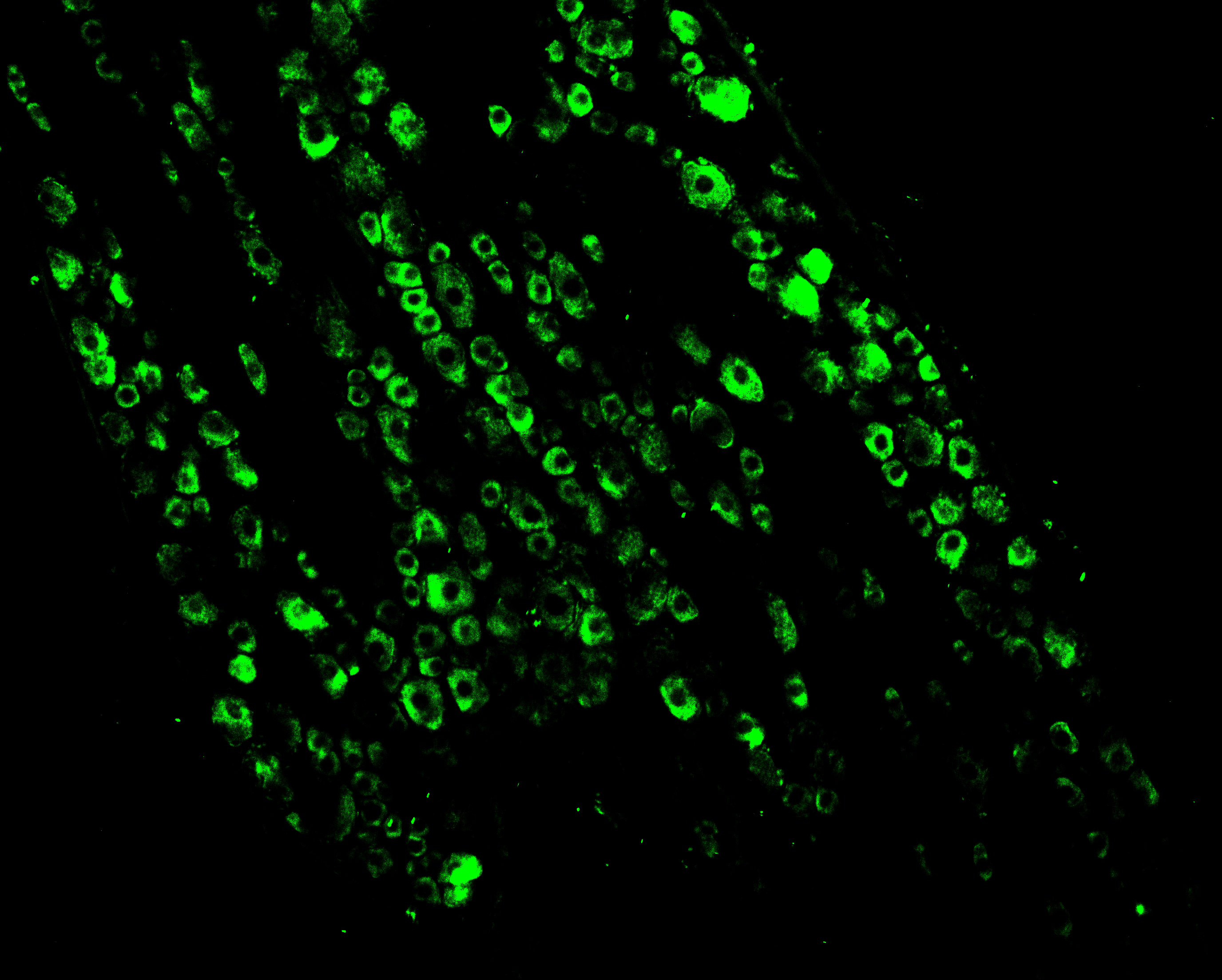

Supplement: Supplementary file 4 — Source data Fig. 2 [file 44319_2024_292_MOESM4_ESM.zip › EMBOR-2024-59294V3-Figure_2_Source_Data-sd/embr202459294-sup-sdatafig2/2F-J/2G.tif]

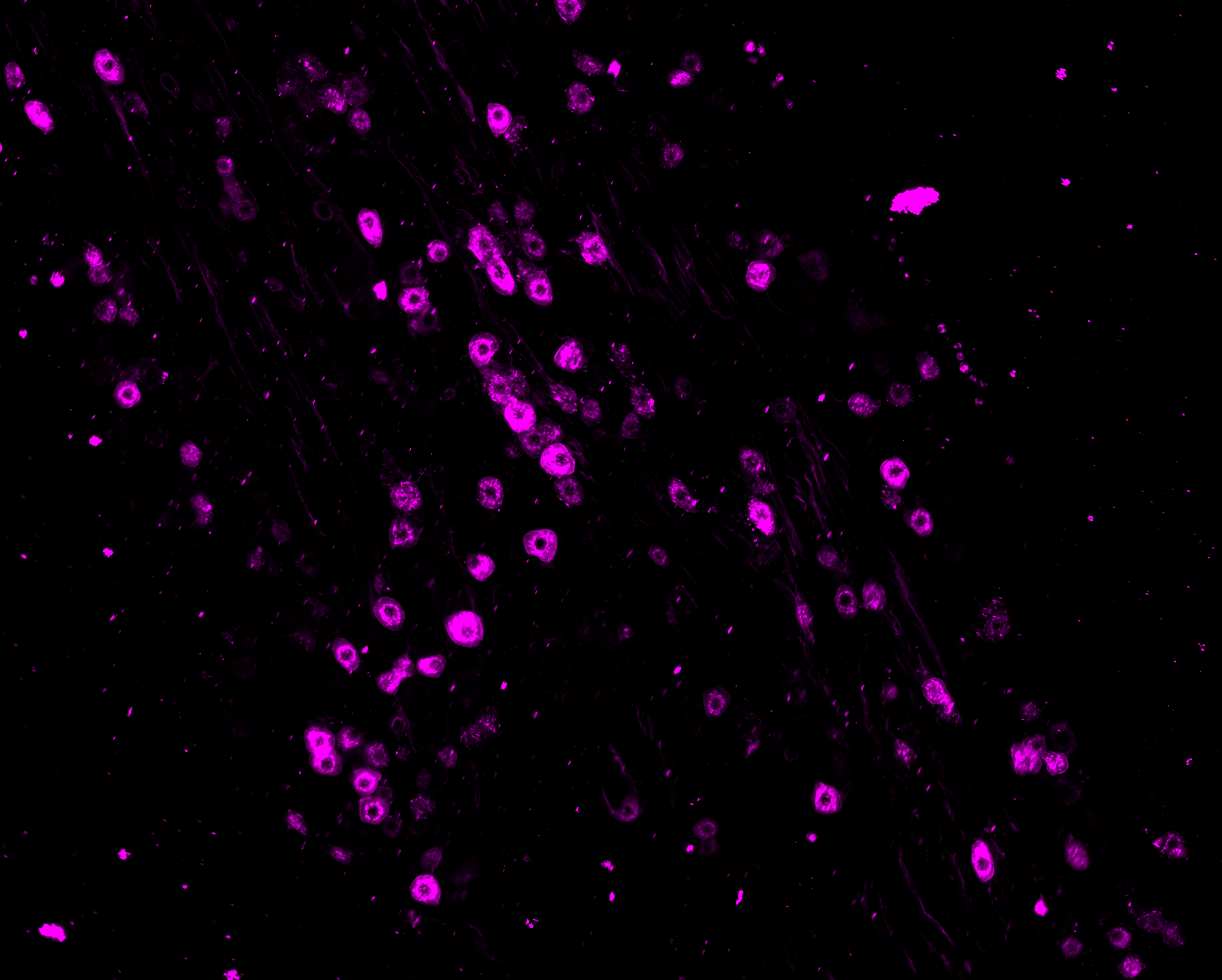

Supplement: Supplementary file 4 — Source data Fig. 2 [file 44319_2024_292_MOESM4_ESM.zip › EMBOR-2024-59294V3-Figure_2_Source_Data-sd/embr202459294-sup-sdatafig2/2F-J/2H.tif]

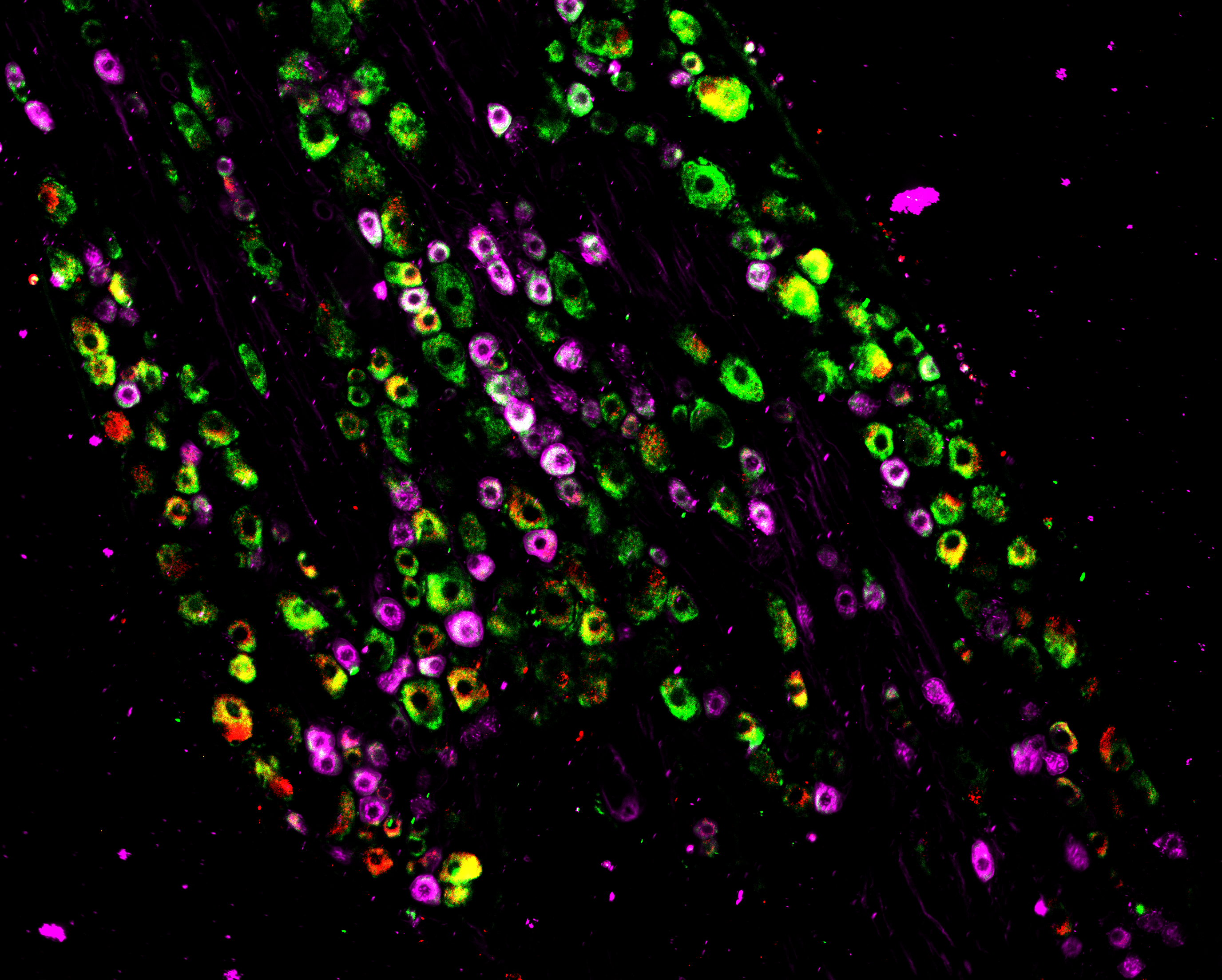

Supplement: Supplementary file 4 — Source data Fig. 2 [file 44319_2024_292_MOESM4_ESM.zip › EMBOR-2024-59294V3-Figure_2_Source_Data-sd/embr202459294-sup-sdatafig2/2F-J/2I.tif]

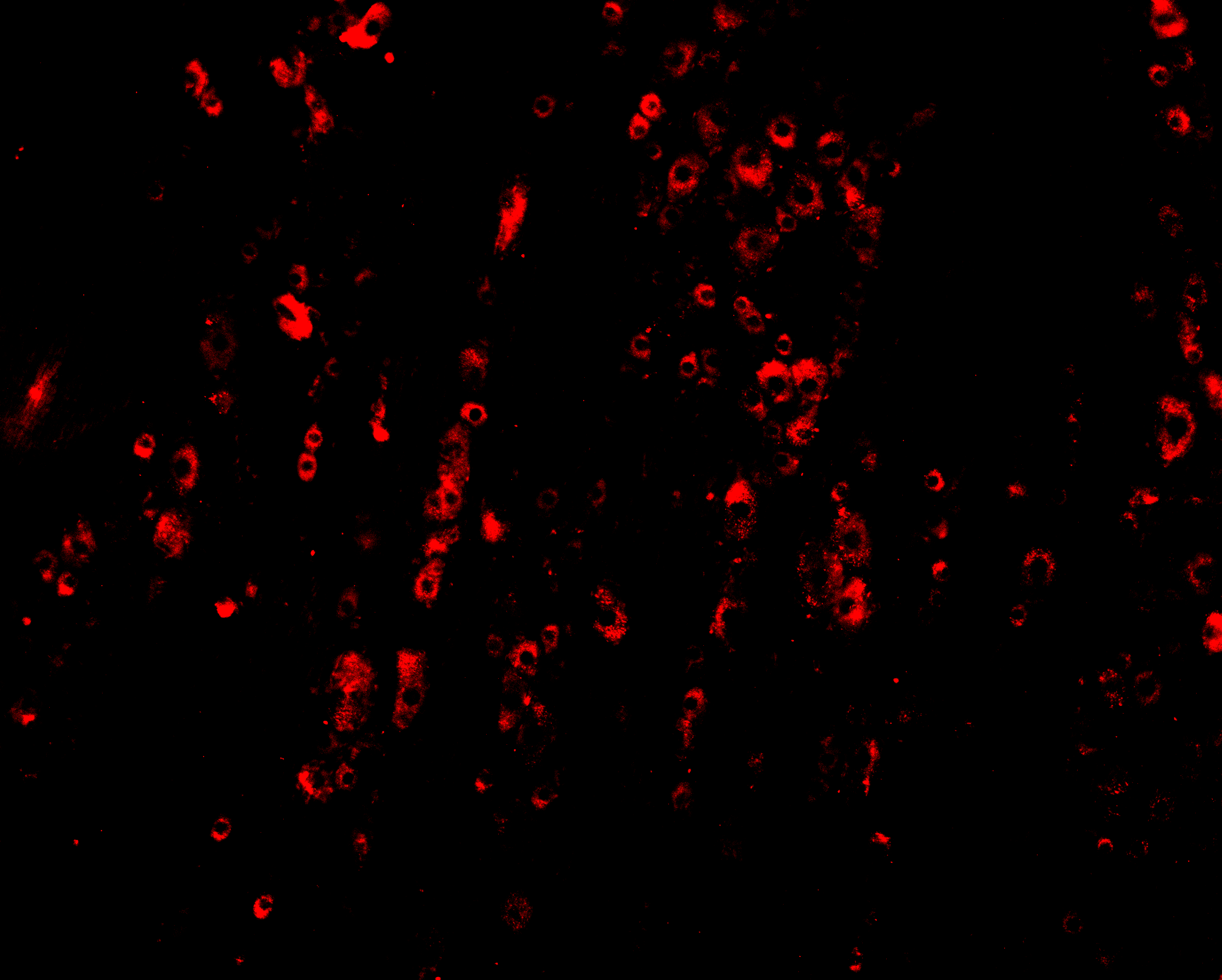

Supplement: Supplementary file 4 — Source data Fig. 2 [file 44319_2024_292_MOESM4_ESM.zip › EMBOR-2024-59294V3-Figure_2_Source_Data-sd/embr202459294-sup-sdatafig2/2K-O/2K.tif]

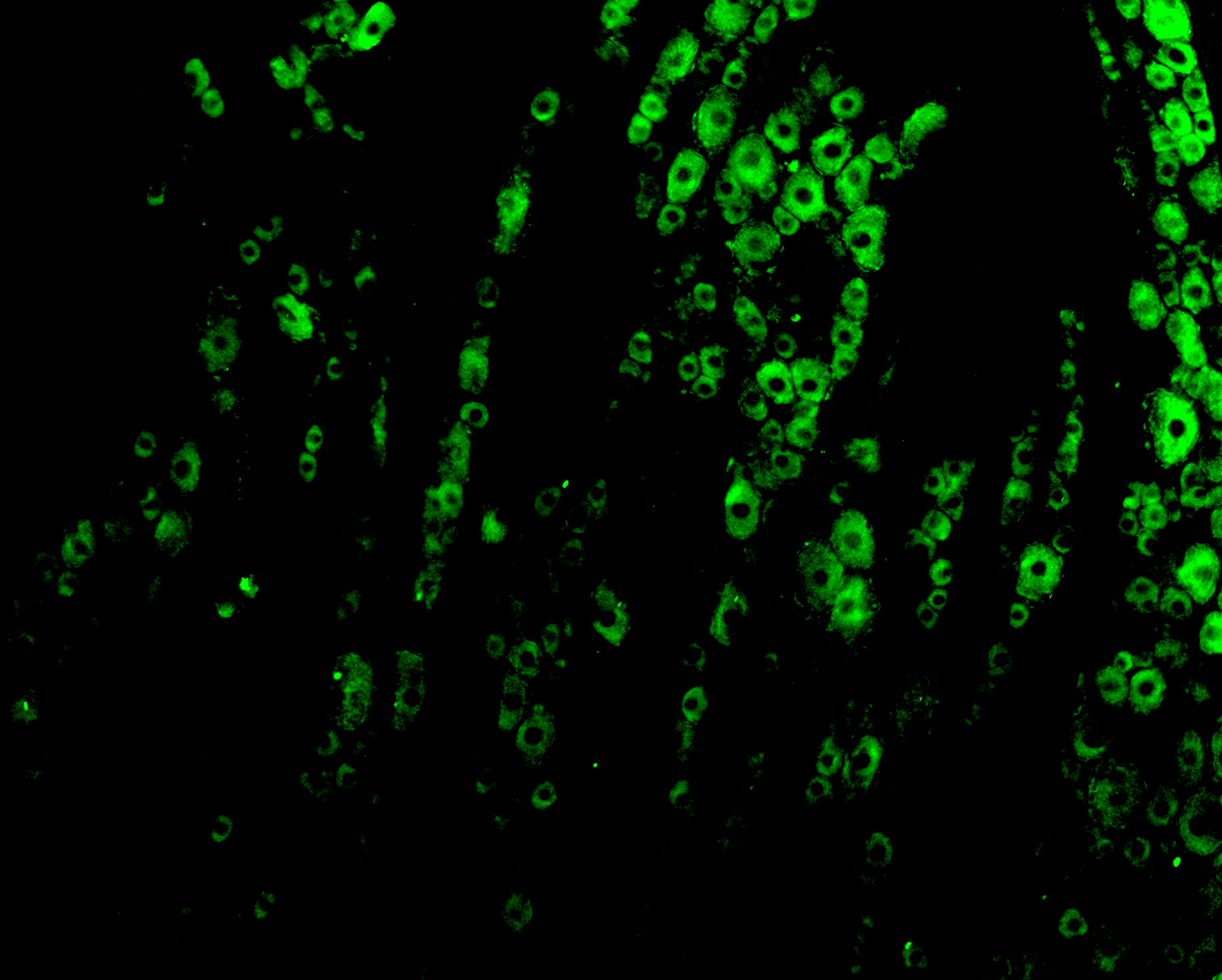

Supplement: Supplementary file 4 — Source data Fig. 2 [file 44319_2024_292_MOESM4_ESM.zip › EMBOR-2024-59294V3-Figure_2_Source_Data-sd/embr202459294-sup-sdatafig2/2K-O/2L.tif]

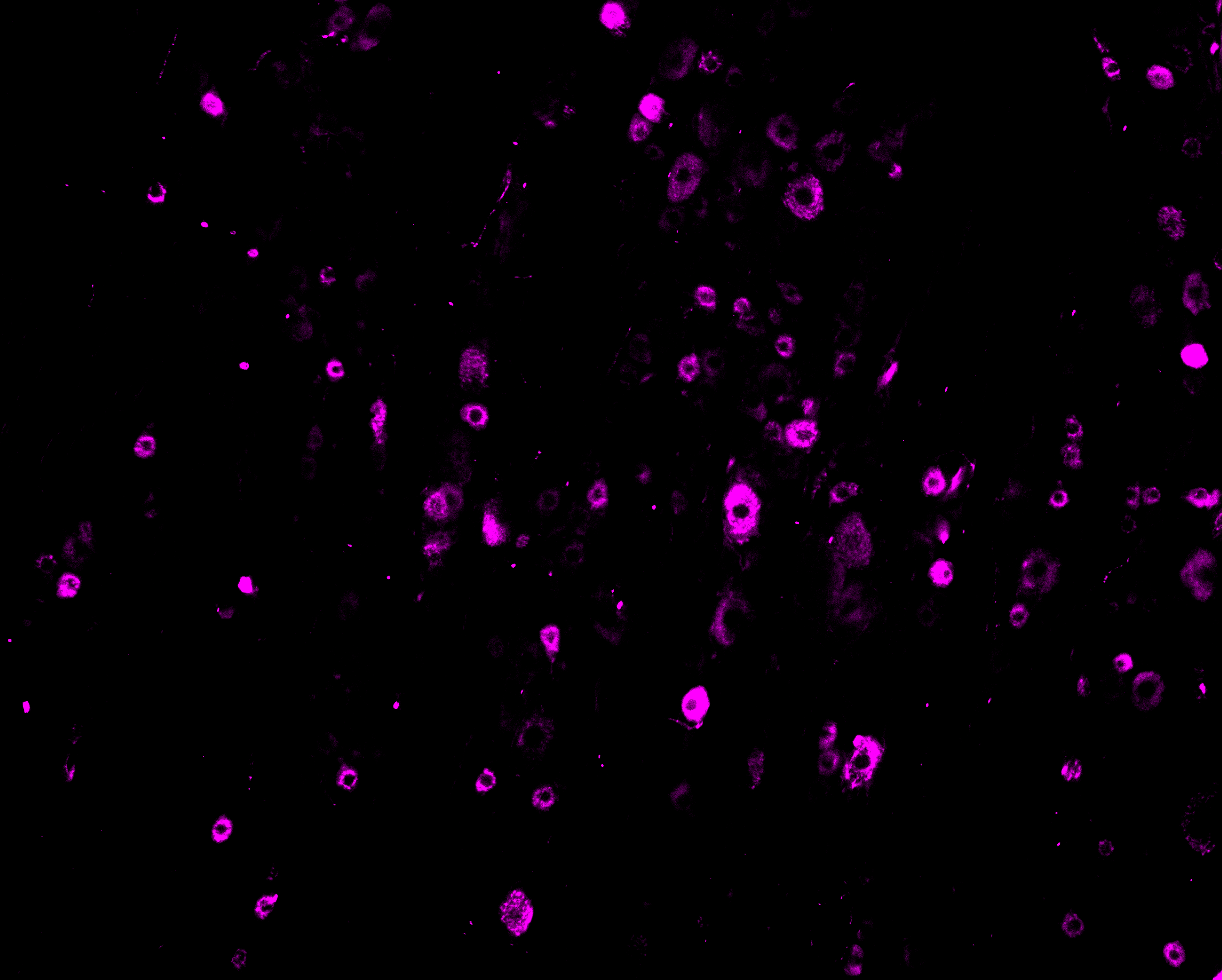

Supplement: Supplementary file 4 — Source data Fig. 2 [file 44319_2024_292_MOESM4_ESM.zip › EMBOR-2024-59294V3-Figure_2_Source_Data-sd/embr202459294-sup-sdatafig2/2K-O/2M.tif]

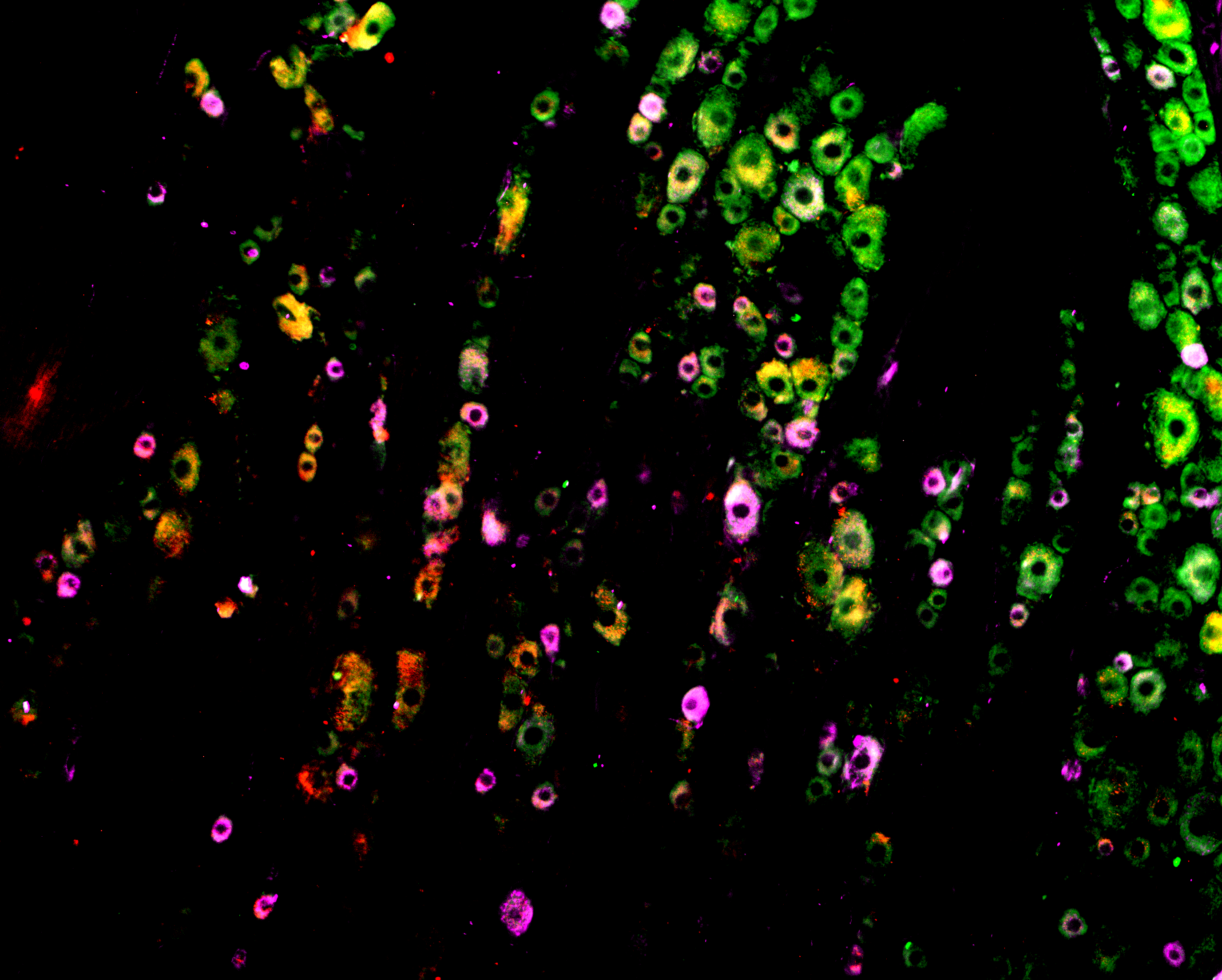

Supplement: Supplementary file 4 — Source data Fig. 2 [file 44319_2024_292_MOESM4_ESM.zip › EMBOR-2024-59294V3-Figure_2_Source_Data-sd/embr202459294-sup-sdatafig2/2K-O/2N.tif]

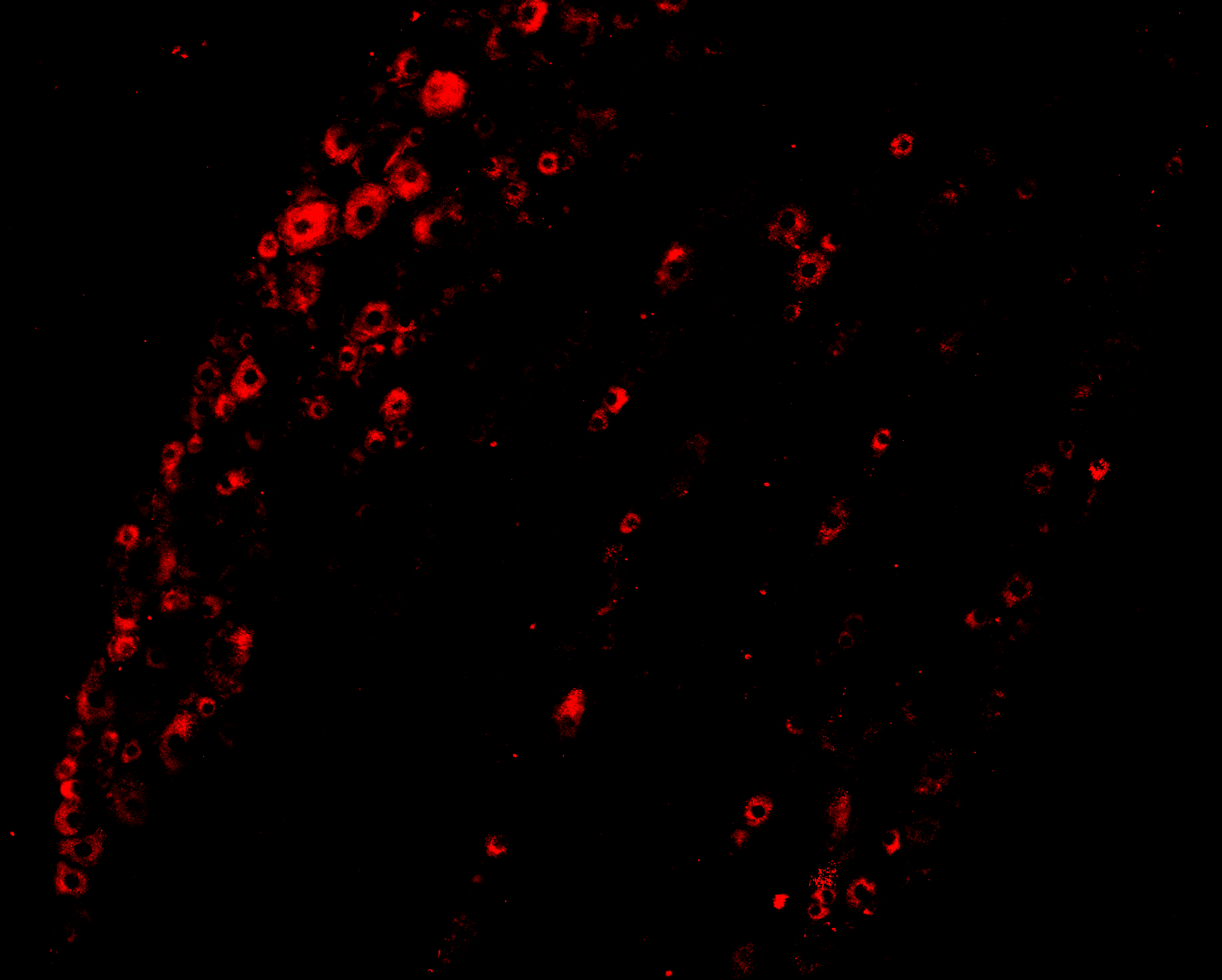

Supplement: Supplementary file 4 — Source data Fig. 2 [file 44319_2024_292_MOESM4_ESM.zip › EMBOR-2024-59294V3-Figure_2_Source_Data-sd/embr202459294-sup-sdatafig2/2P-T/2P.tif]

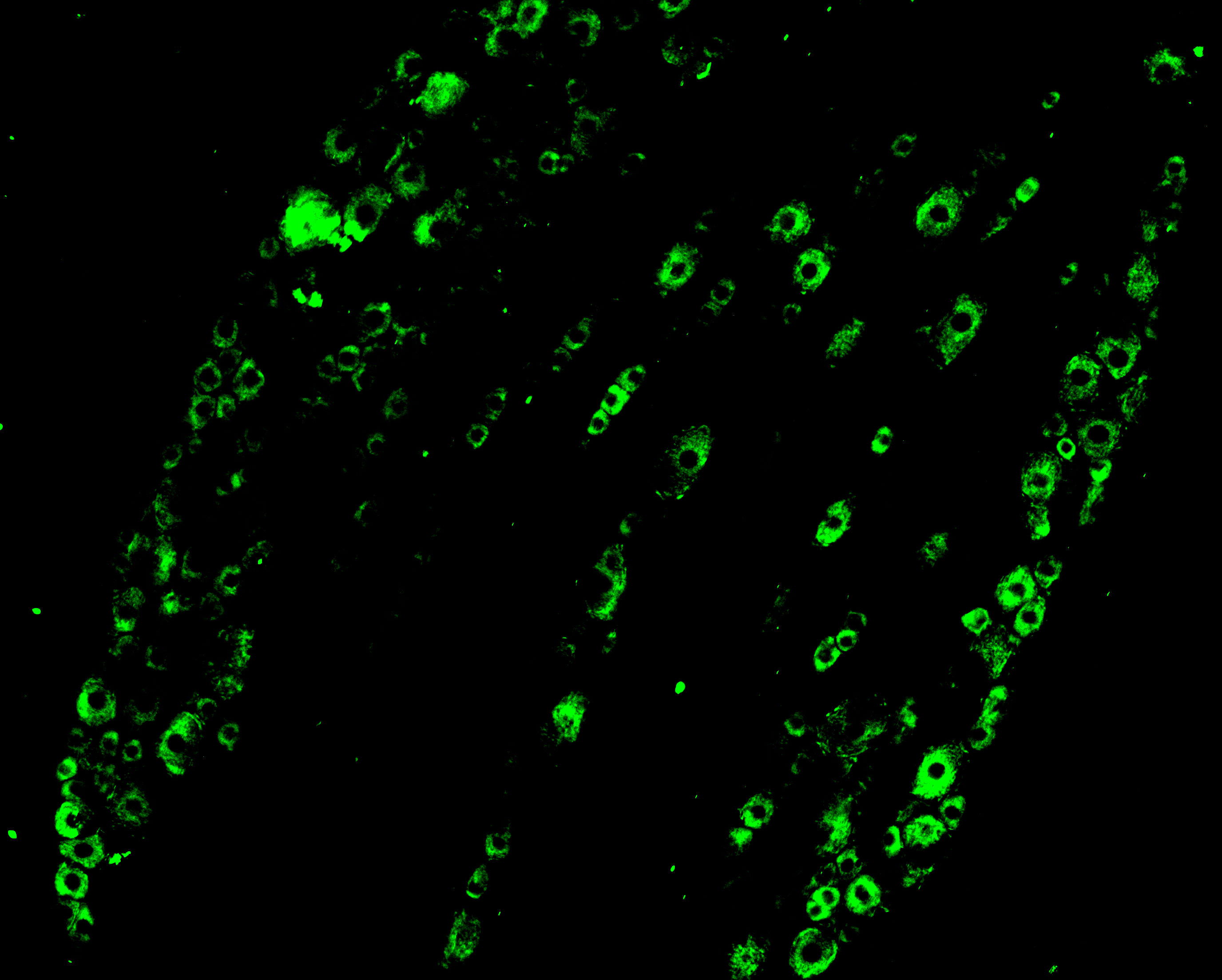

Supplement: Supplementary file 4 — Source data Fig. 2 [file 44319_2024_292_MOESM4_ESM.zip › EMBOR-2024-59294V3-Figure_2_Source_Data-sd/embr202459294-sup-sdatafig2/2P-T/2Q.tif]

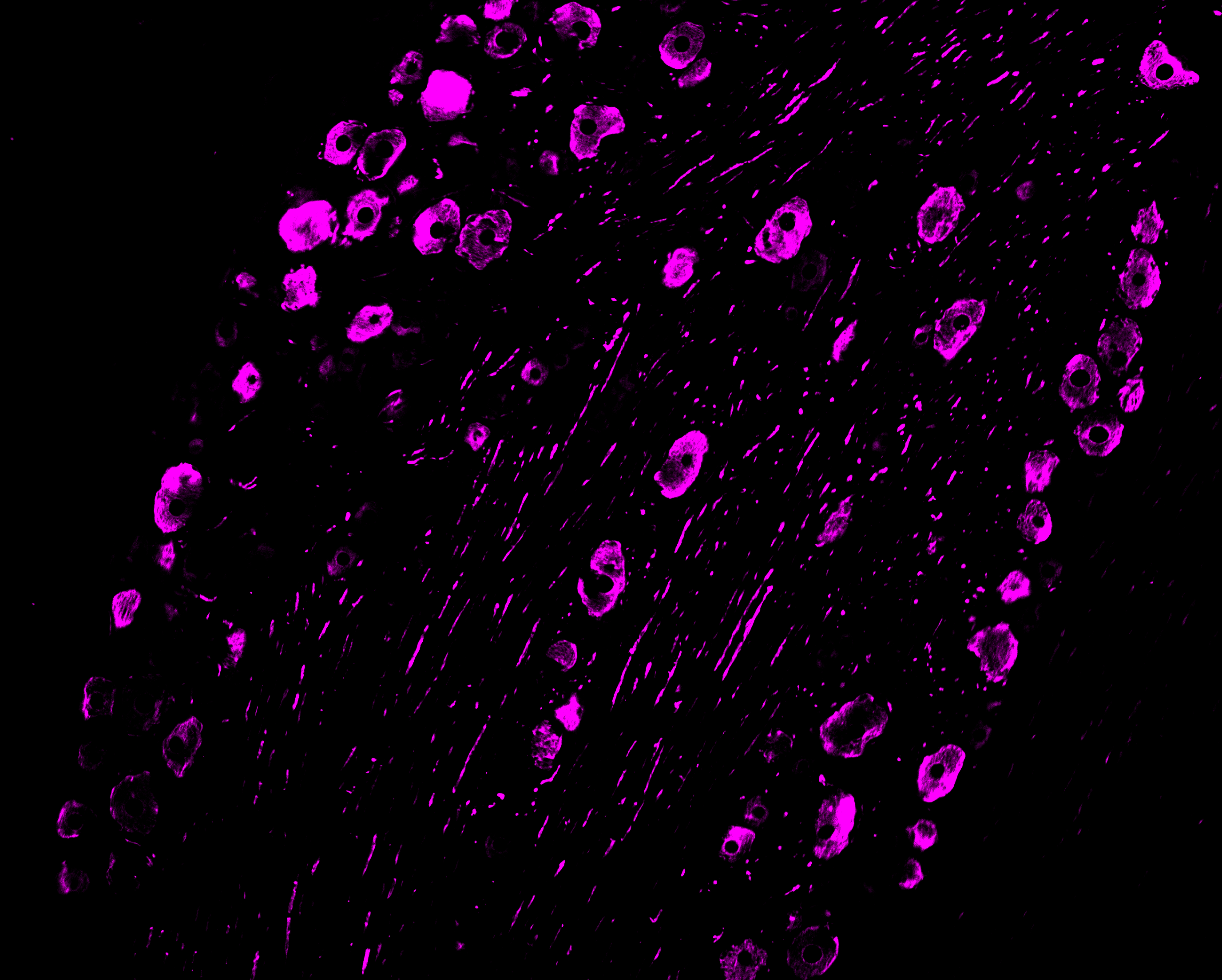

Supplement: Supplementary file 4 — Source data Fig. 2 [file 44319_2024_292_MOESM4_ESM.zip › EMBOR-2024-59294V3-Figure_2_Source_Data-sd/embr202459294-sup-sdatafig2/2P-T/2R.tif]

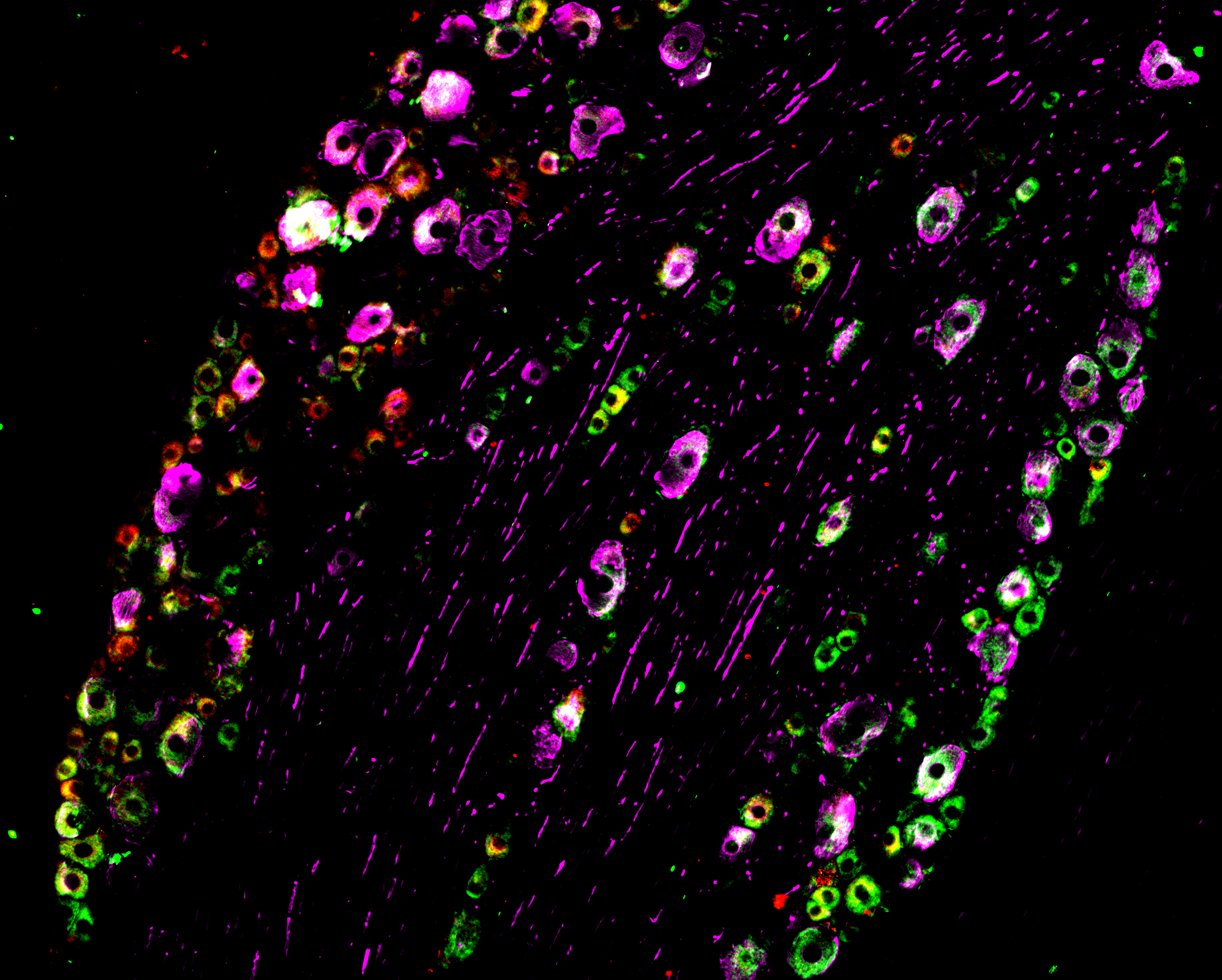

Supplement: Supplementary file 4 — Source data Fig. 2 [file 44319_2024_292_MOESM4_ESM.zip › EMBOR-2024-59294V3-Figure_2_Source_Data-sd/embr202459294-sup-sdatafig2/2P-T/2S.tif]

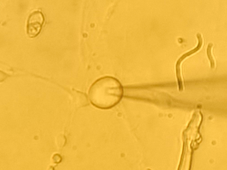

Supplement: Supplementary file 7 — Source data Fig. 5 [file 44319_2024_292_MOESM7_ESM.zip › EMBOR-2024-59294V3-Figure_5_Source_Data-sd/embr202459294-sup-sdatafig5/5A/5A.png]

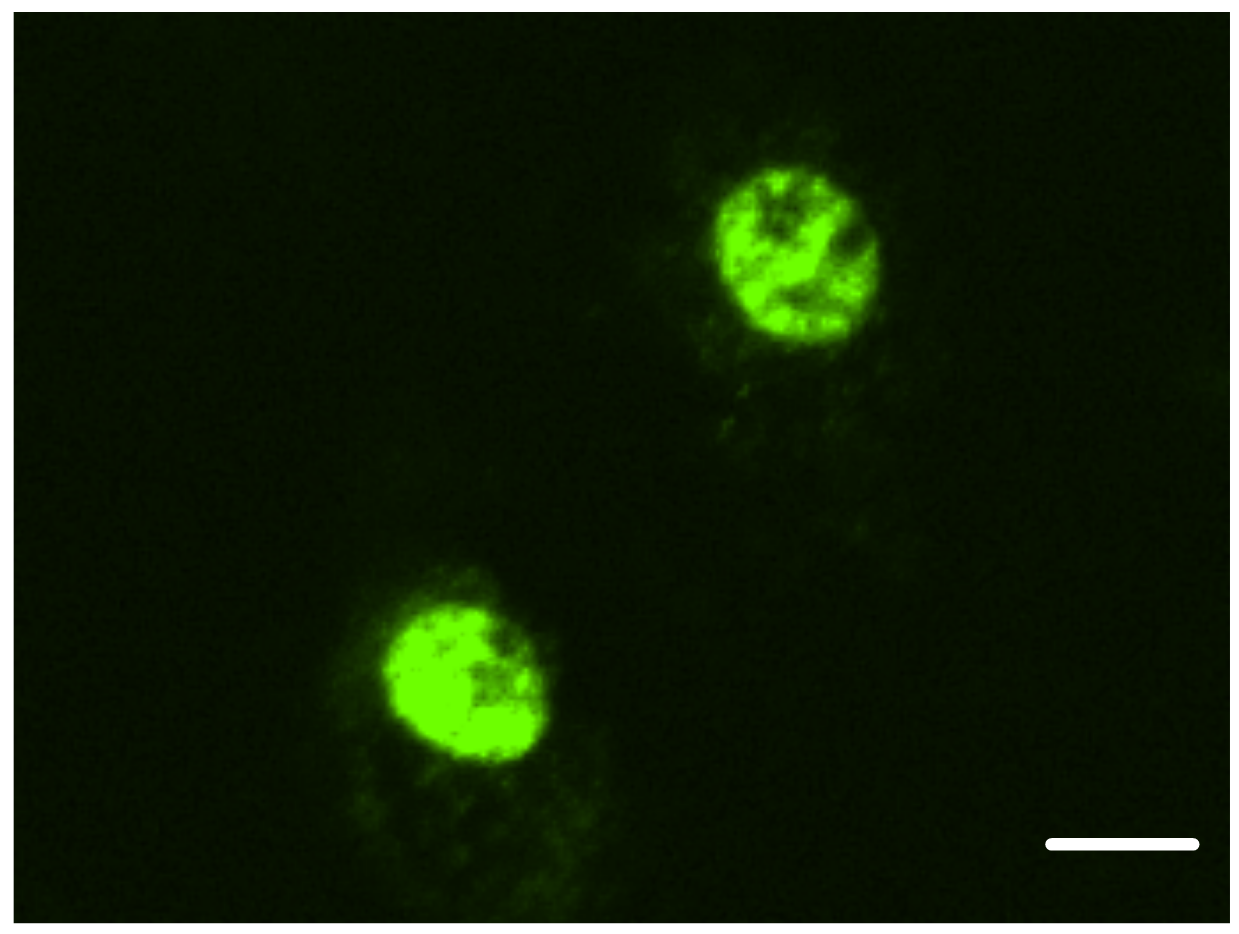

Supplement: Supplementary file 7 — Source data Fig. 5 [file 44319_2024_292_MOESM7_ESM.zip › EMBOR-2024-59294V3-Figure_5_Source_Data-sd/embr202459294-sup-sdatafig5/5H/5H.jpg]

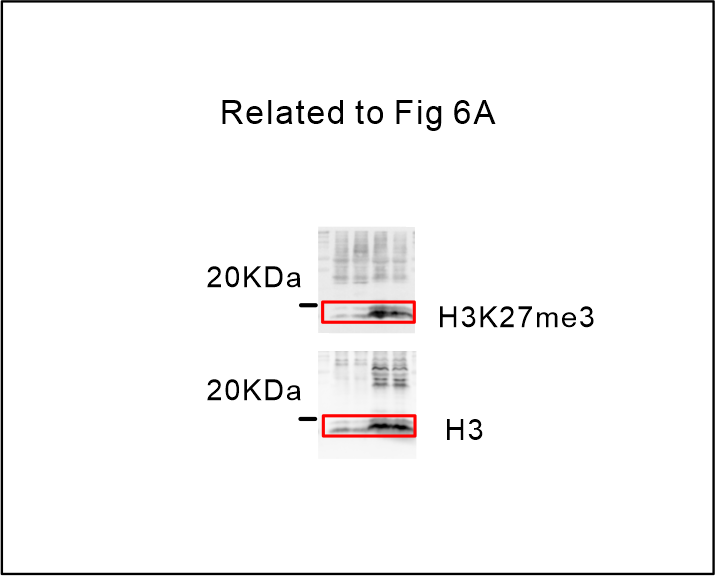

Supplement: Supplementary file 8 — Source data Fig. 6 [file 44319_2024_292_MOESM8_ESM.zip › EMBOR-2024-59294V3-Figure_6_Source_Data-sd/embr202459294-sup-sdatafig6/6A/6A.tif]

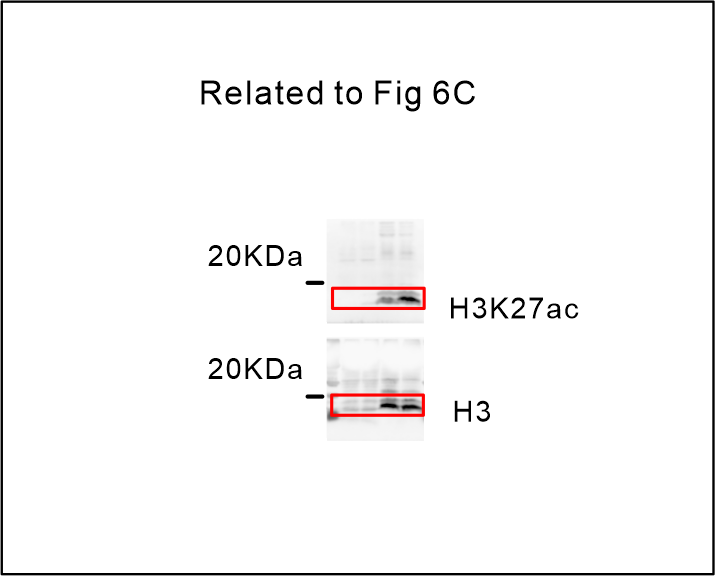

Supplement: Supplementary file 8 — Source data Fig. 6 [file 44319_2024_292_MOESM8_ESM.zip › EMBOR-2024-59294V3-Figure_6_Source_Data-sd/embr202459294-sup-sdatafig6/6C/6C.tif]

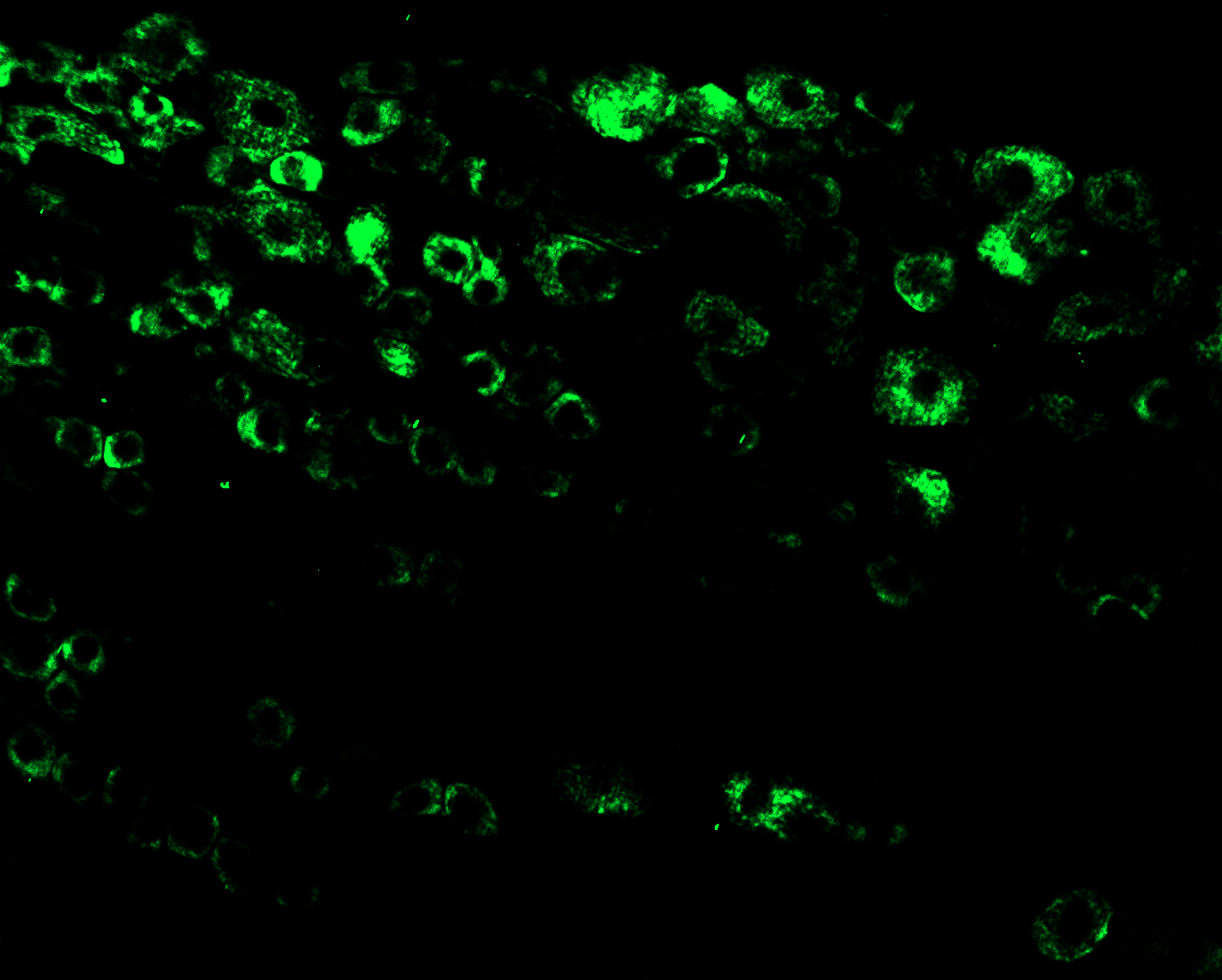

Supplement: Supplementary file 8 — Source data Fig. 6 [file 44319_2024_292_MOESM8_ESM.zip › EMBOR-2024-59294V3-Figure_6_Source_Data-sd/embr202459294-sup-sdatafig6/6E/1-1.tif]

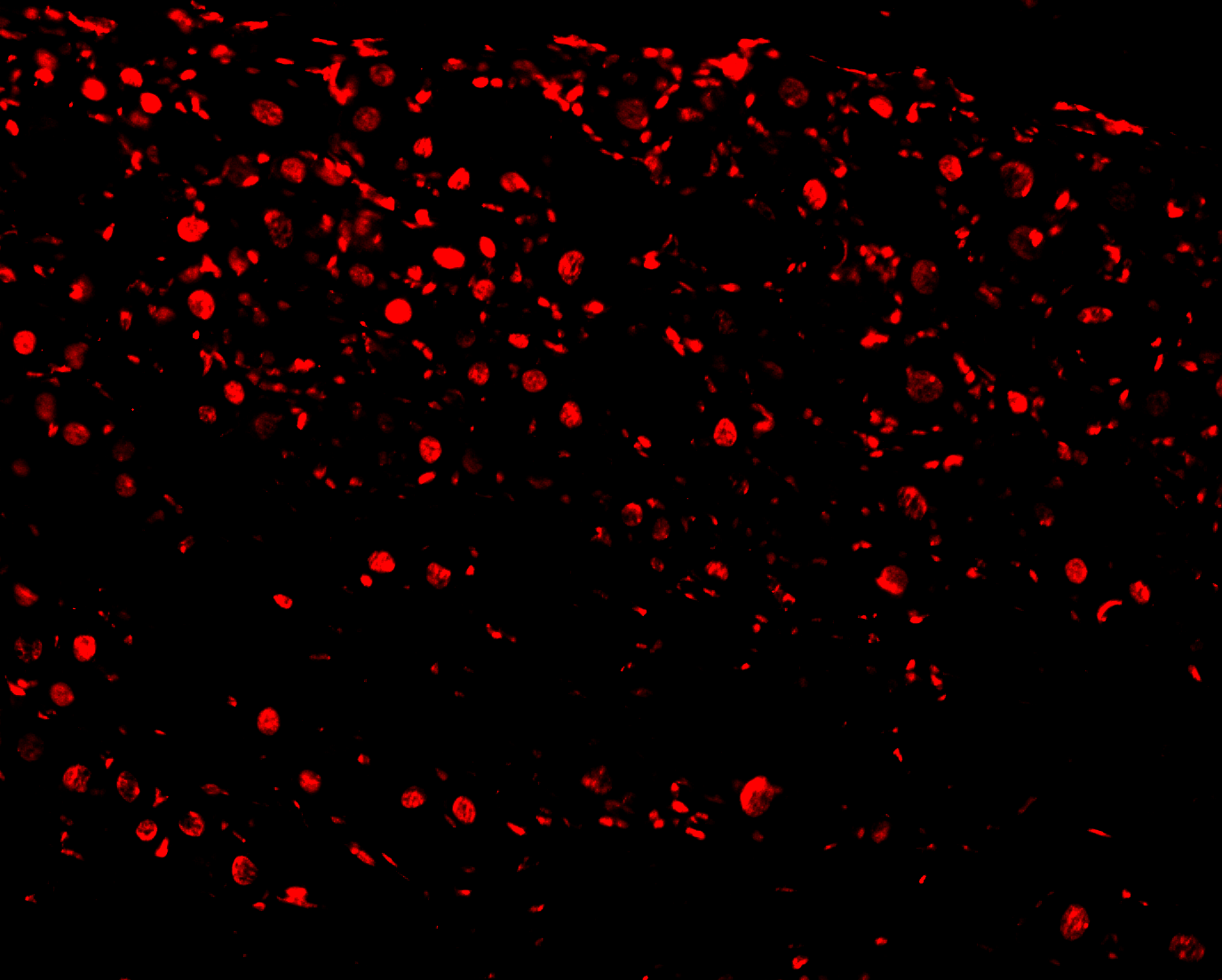

Supplement: Supplementary file 8 — Source data Fig. 6 [file 44319_2024_292_MOESM8_ESM.zip › EMBOR-2024-59294V3-Figure_6_Source_Data-sd/embr202459294-sup-sdatafig6/6E/1-2.tif]

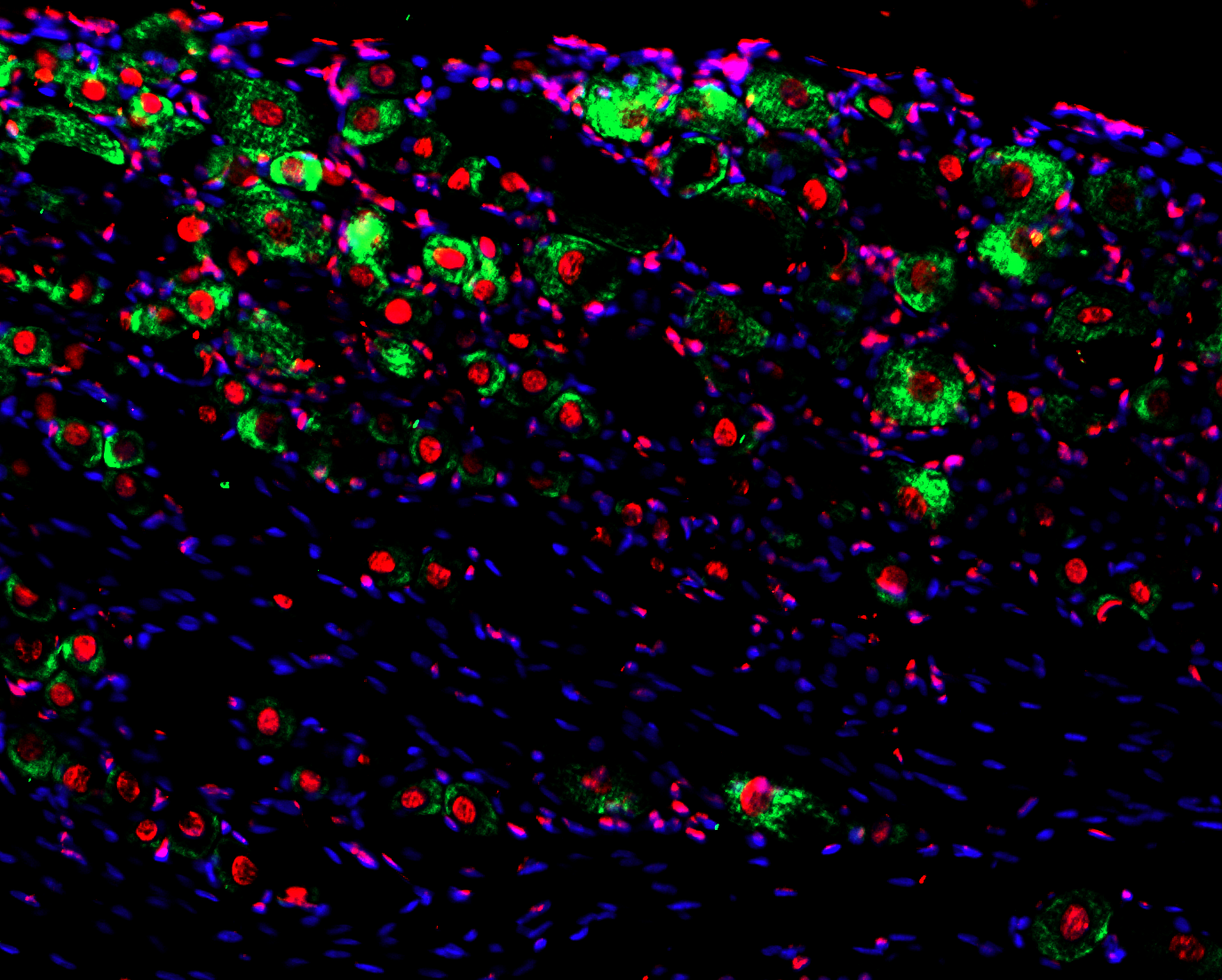

Supplement: Supplementary file 8 — Source data Fig. 6 [file 44319_2024_292_MOESM8_ESM.zip › EMBOR-2024-59294V3-Figure_6_Source_Data-sd/embr202459294-sup-sdatafig6/6E/1-3.tif]

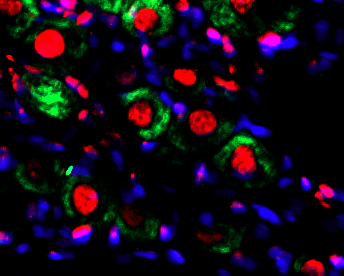

Supplement: Supplementary file 8 — Source data Fig. 6 [file 44319_2024_292_MOESM8_ESM.zip › EMBOR-2024-59294V3-Figure_6_Source_Data-sd/embr202459294-sup-sdatafig6/6E/1-4.tif]

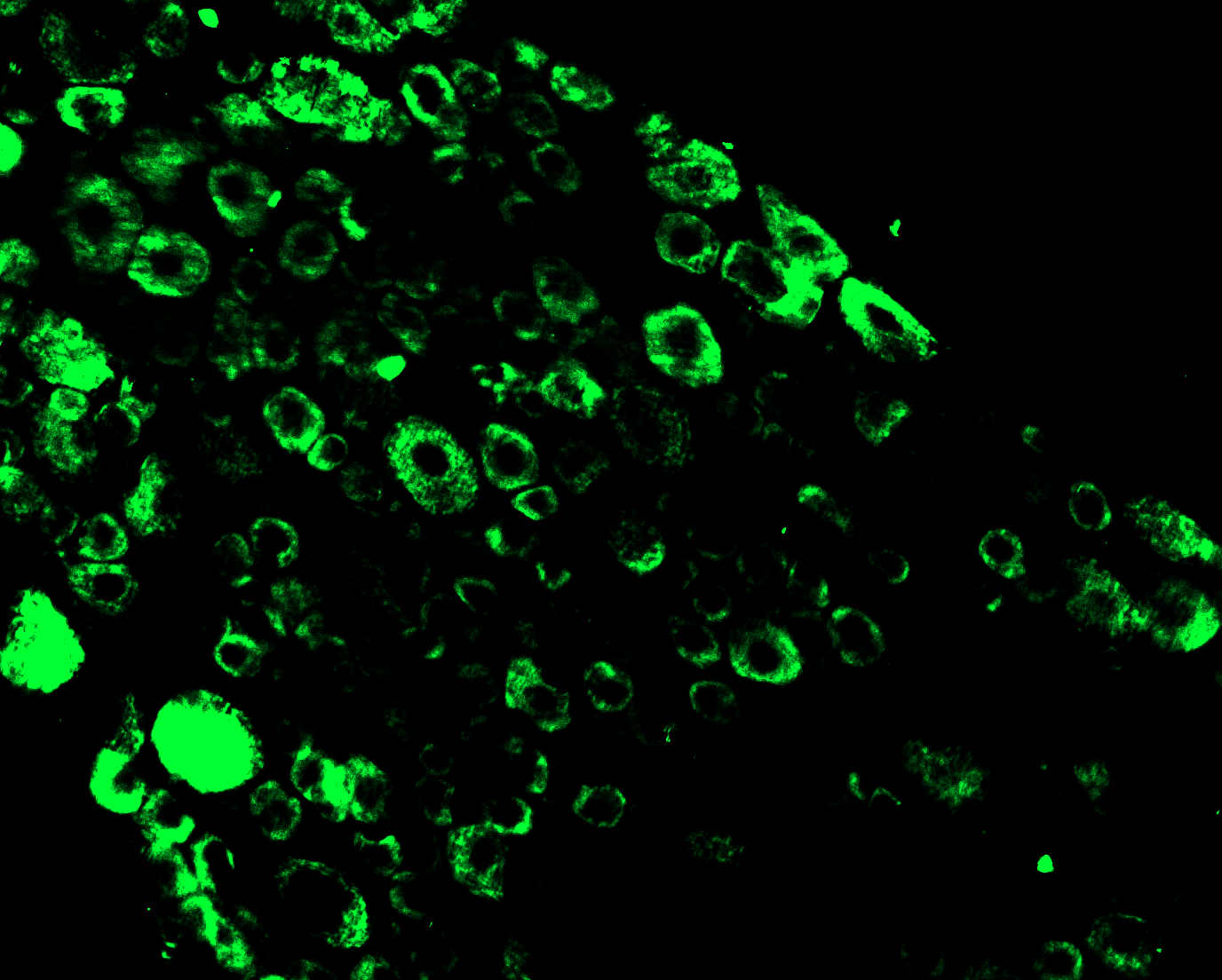

Supplement: Supplementary file 8 — Source data Fig. 6 [file 44319_2024_292_MOESM8_ESM.zip › EMBOR-2024-59294V3-Figure_6_Source_Data-sd/embr202459294-sup-sdatafig6/6E/2-1.tif]

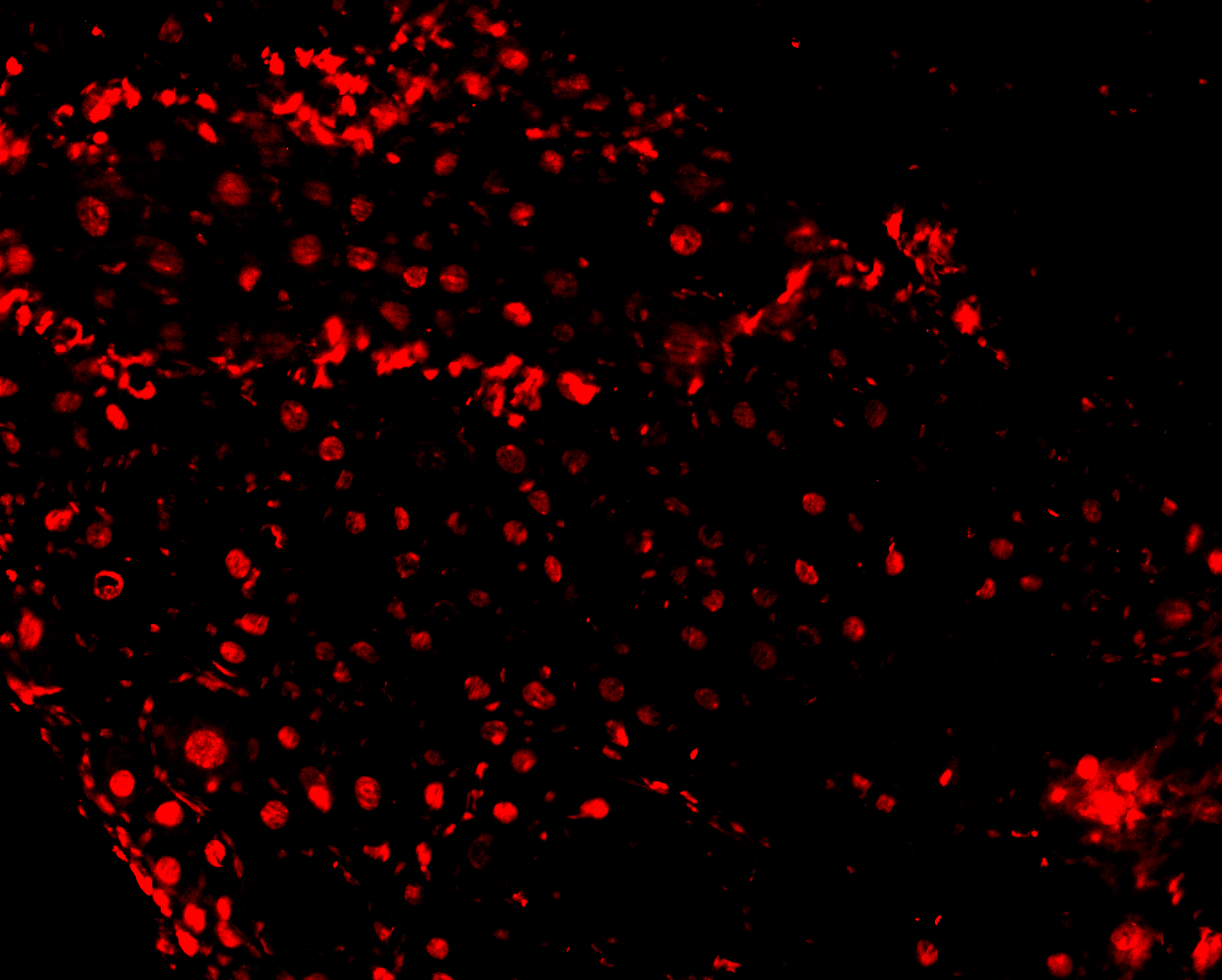

Supplement: Supplementary file 8 — Source data Fig. 6 [file 44319_2024_292_MOESM8_ESM.zip › EMBOR-2024-59294V3-Figure_6_Source_Data-sd/embr202459294-sup-sdatafig6/6E/2-2.tif]

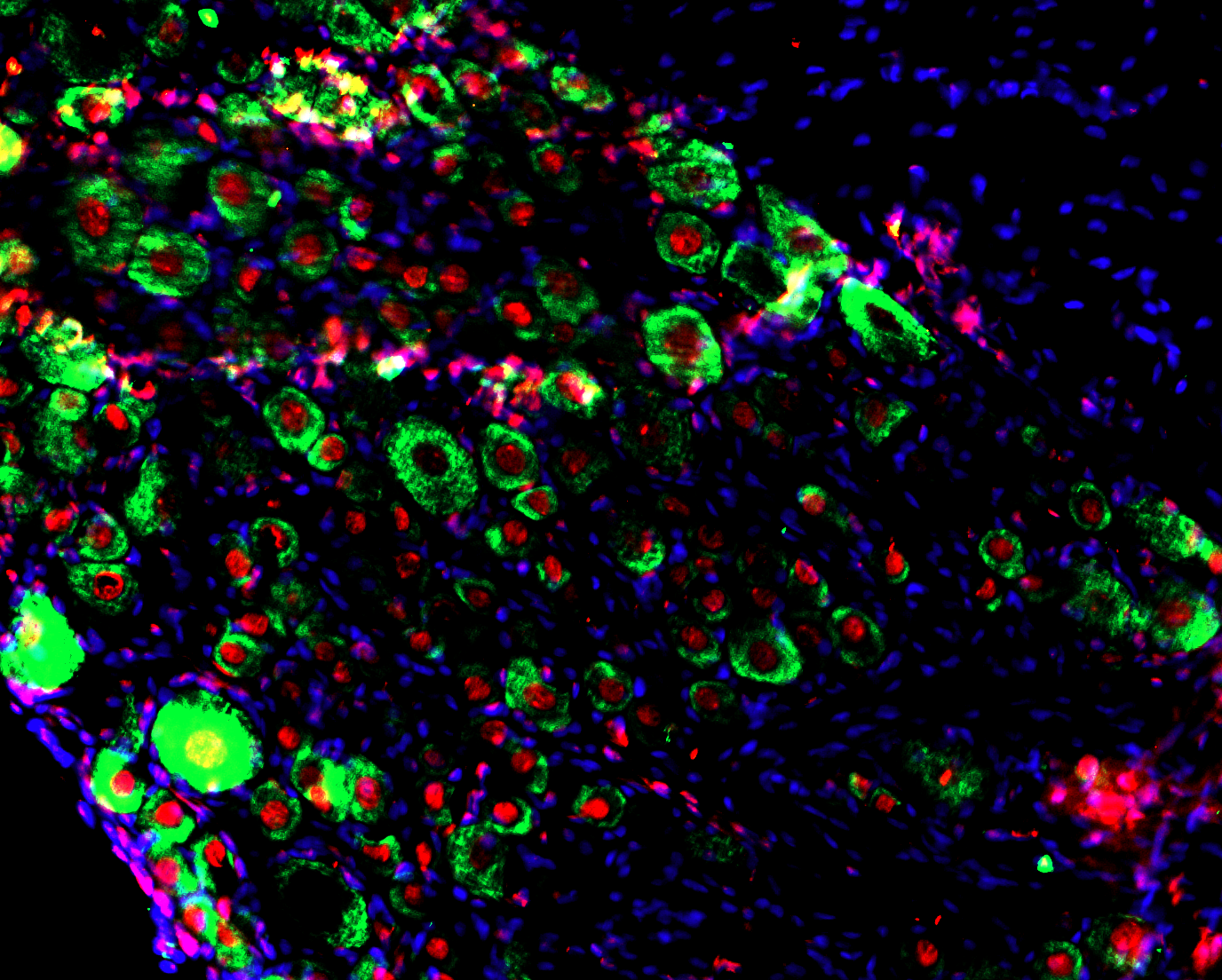

Supplement: Supplementary file 8 — Source data Fig. 6 [file 44319_2024_292_MOESM8_ESM.zip › EMBOR-2024-59294V3-Figure_6_Source_Data-sd/embr202459294-sup-sdatafig6/6E/2-3.tif]

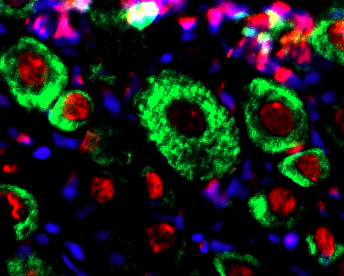

Supplement: Supplementary file 8 — Source data Fig. 6 [file 44319_2024_292_MOESM8_ESM.zip › EMBOR-2024-59294V3-Figure_6_Source_Data-sd/embr202459294-sup-sdatafig6/6E/2-4.tif]

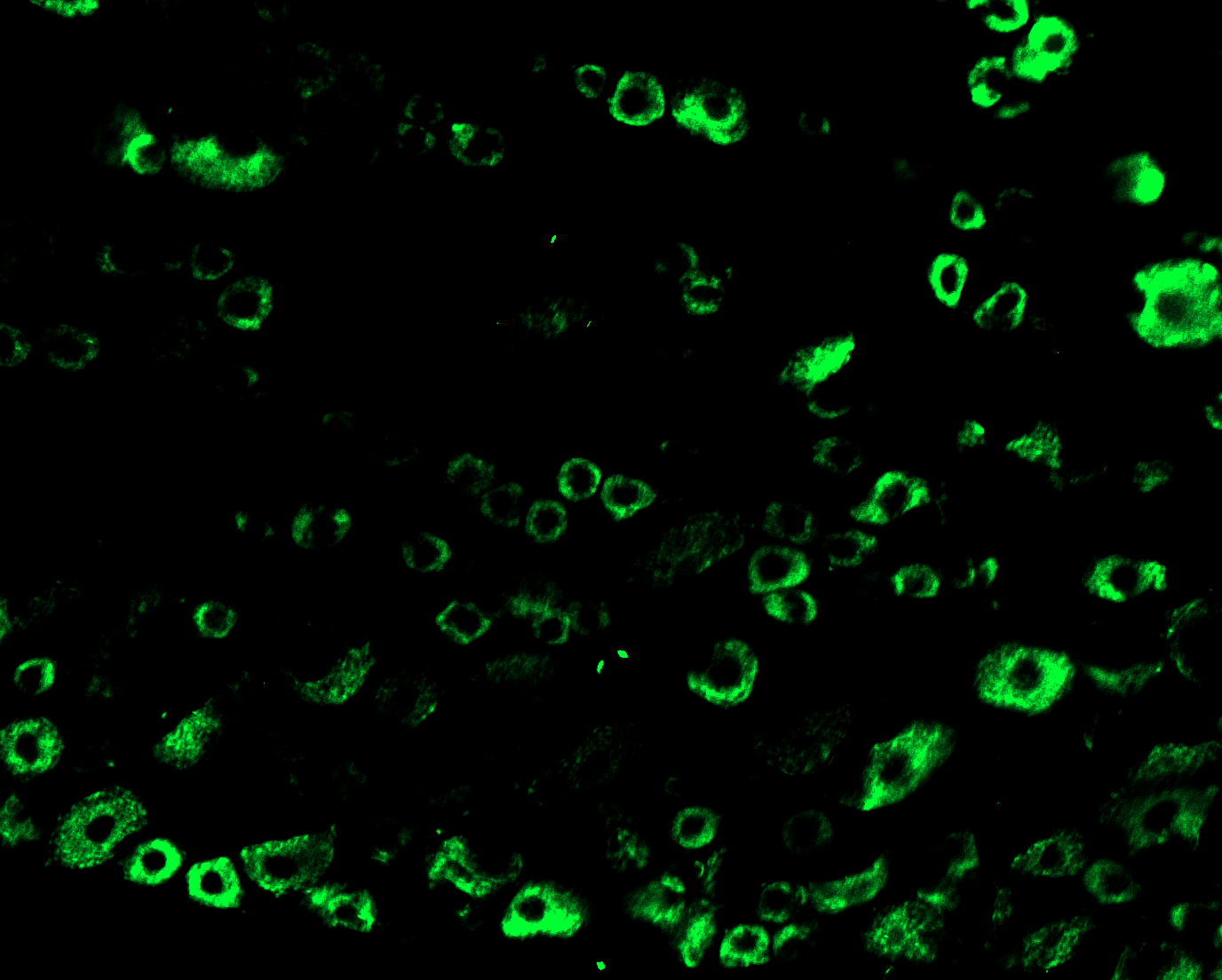

Supplement: Supplementary file 8 — Source data Fig. 6 [file 44319_2024_292_MOESM8_ESM.zip › EMBOR-2024-59294V3-Figure_6_Source_Data-sd/embr202459294-sup-sdatafig6/6E/3-1.tif]

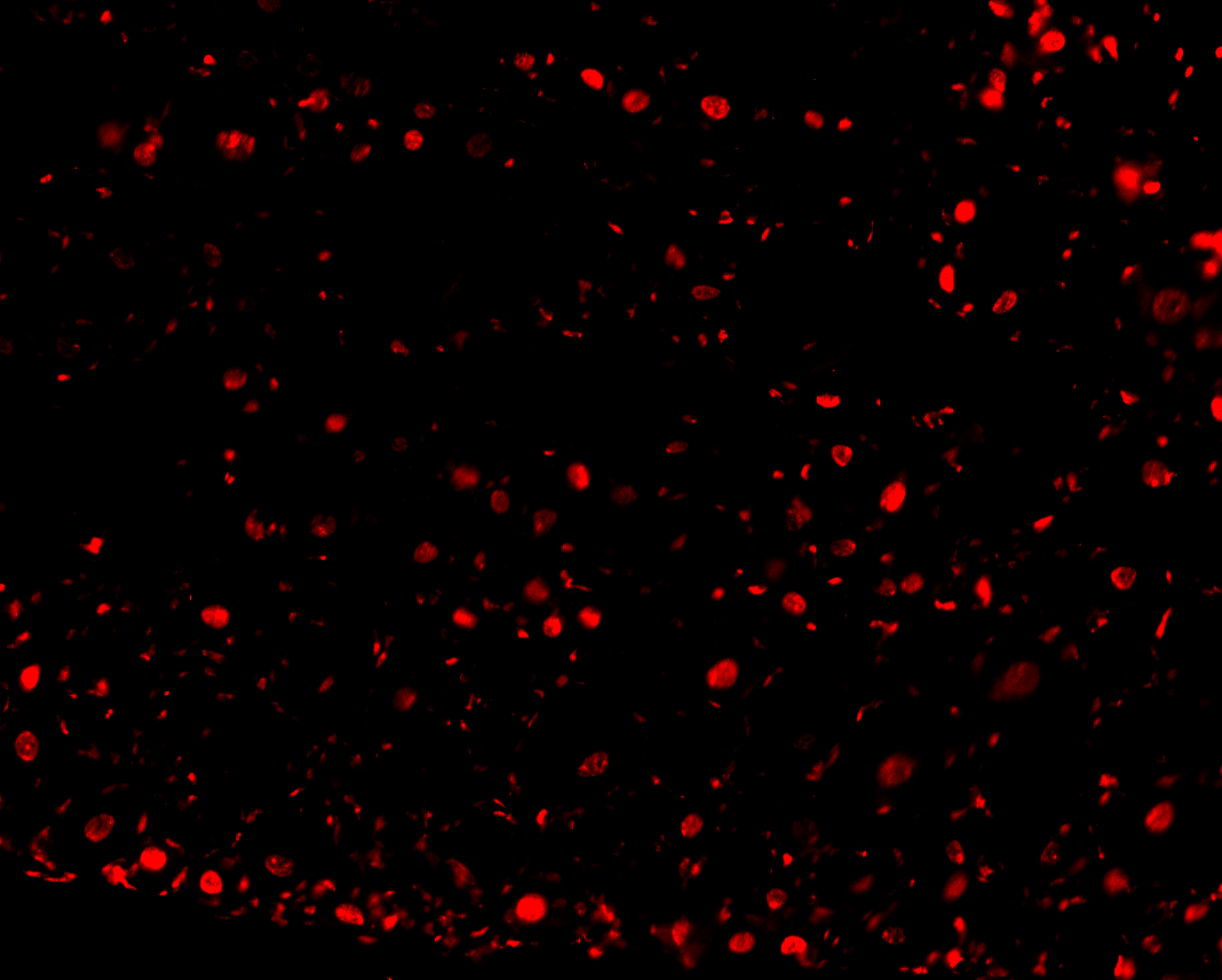

Supplement: Supplementary file 8 — Source data Fig. 6 [file 44319_2024_292_MOESM8_ESM.zip › EMBOR-2024-59294V3-Figure_6_Source_Data-sd/embr202459294-sup-sdatafig6/6E/3-2.tif]

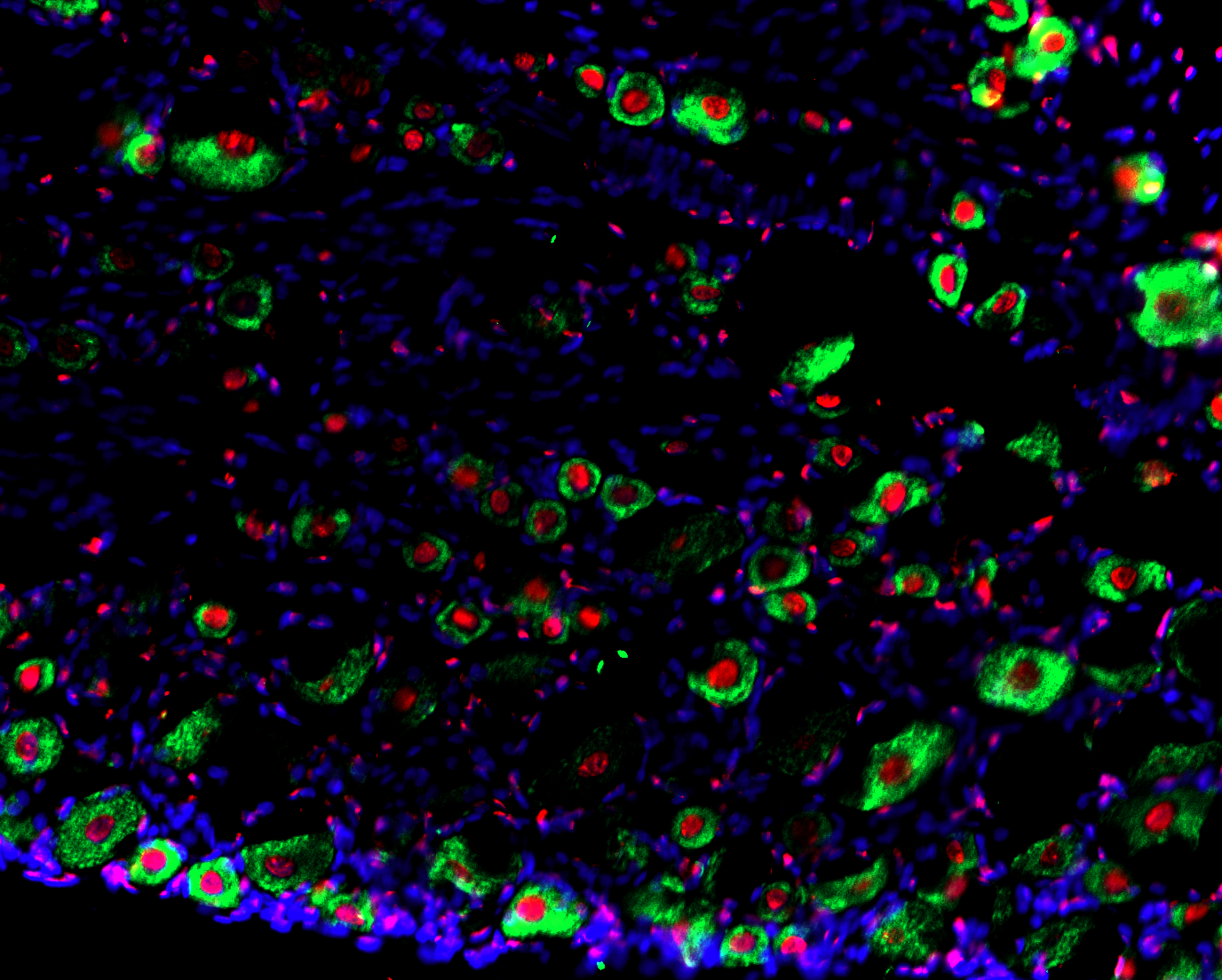

Supplement: Supplementary file 8 — Source data Fig. 6 [file 44319_2024_292_MOESM8_ESM.zip › EMBOR-2024-59294V3-Figure_6_Source_Data-sd/embr202459294-sup-sdatafig6/6E/3-3.tif]

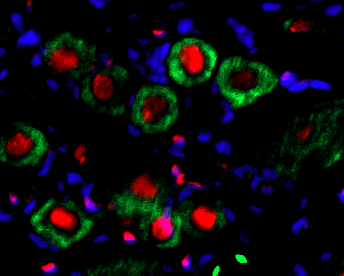

Supplement: Supplementary file 8 — Source data Fig. 6 [file 44319_2024_292_MOESM8_ESM.zip › EMBOR-2024-59294V3-Figure_6_Source_Data-sd/embr202459294-sup-sdatafig6/6E/3-4.tif]

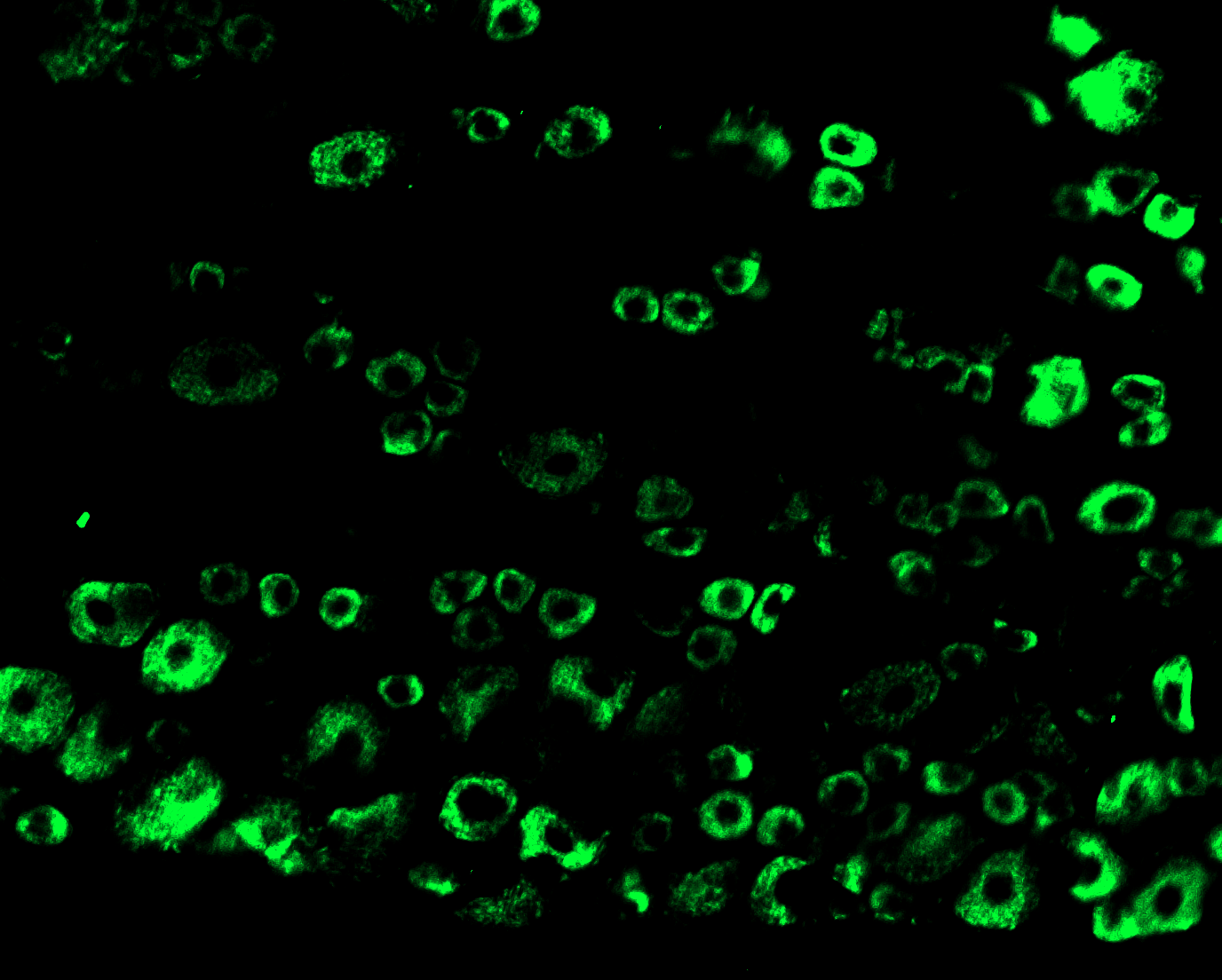

Supplement: Supplementary file 8 — Source data Fig. 6 [file 44319_2024_292_MOESM8_ESM.zip › EMBOR-2024-59294V3-Figure_6_Source_Data-sd/embr202459294-sup-sdatafig6/6E/4-1.tif]

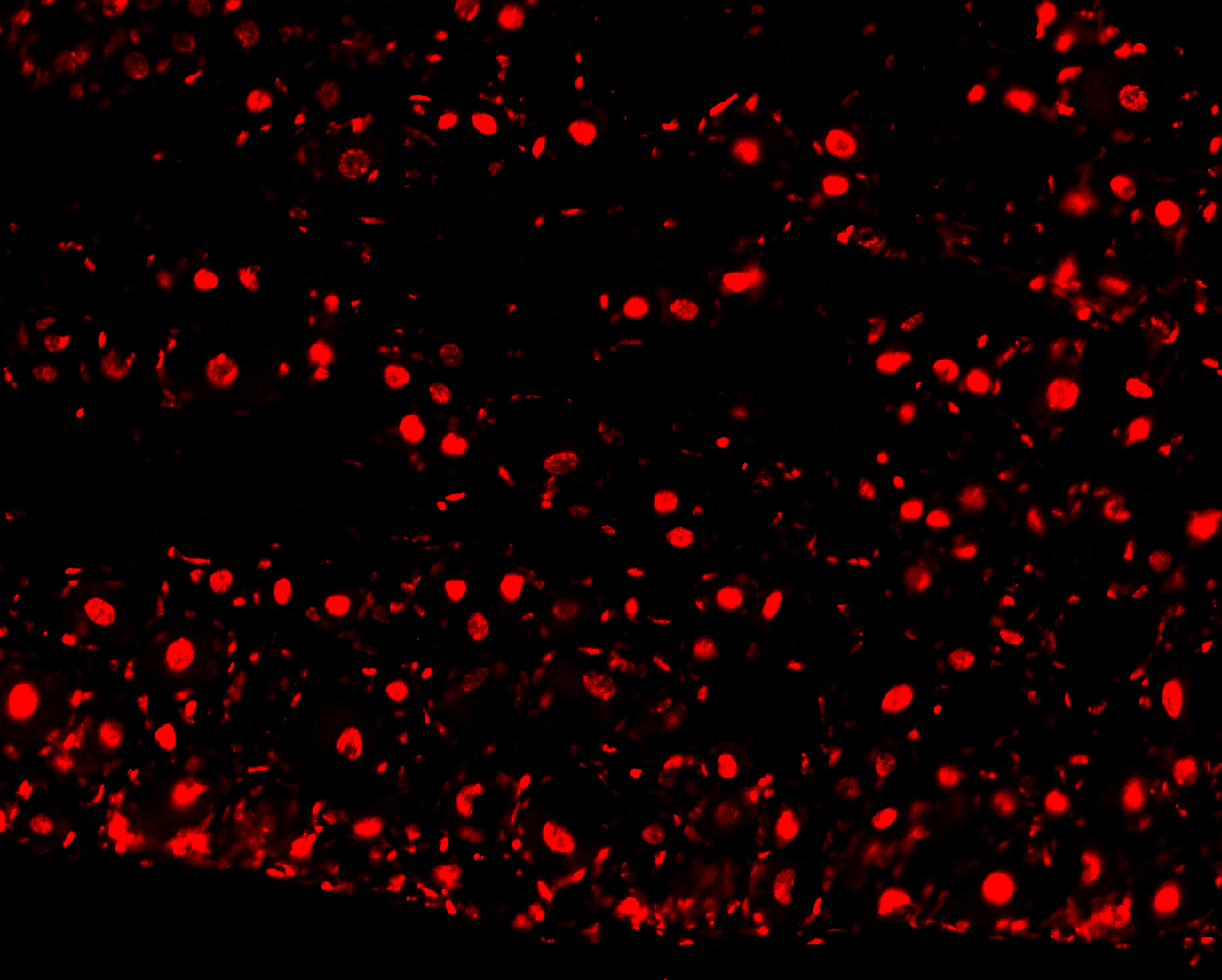

Supplement: Supplementary file 8 — Source data Fig. 6 [file 44319_2024_292_MOESM8_ESM.zip › EMBOR-2024-59294V3-Figure_6_Source_Data-sd/embr202459294-sup-sdatafig6/6E/4-2.tif]

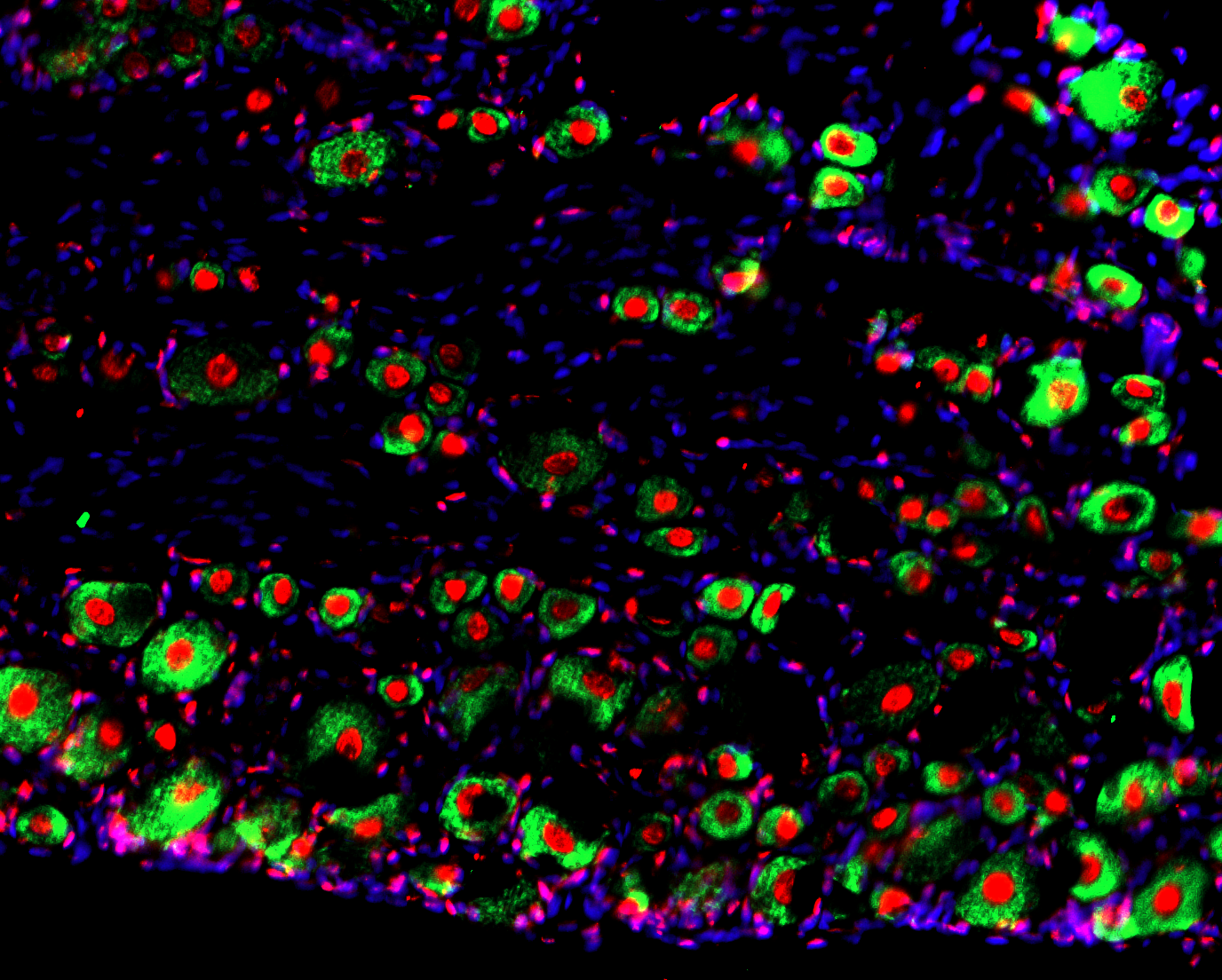

Supplement: Supplementary file 8 — Source data Fig. 6 [file 44319_2024_292_MOESM8_ESM.zip › EMBOR-2024-59294V3-Figure_6_Source_Data-sd/embr202459294-sup-sdatafig6/6E/4-3.tif]

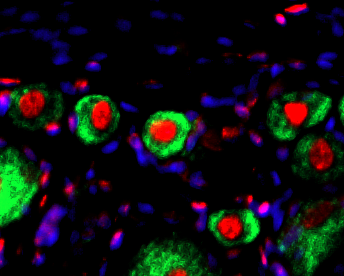

Supplement: Supplementary file 8 — Source data Fig. 6 [file 44319_2024_292_MOESM8_ESM.zip › EMBOR-2024-59294V3-Figure_6_Source_Data-sd/embr202459294-sup-sdatafig6/6E/4-4.tif]

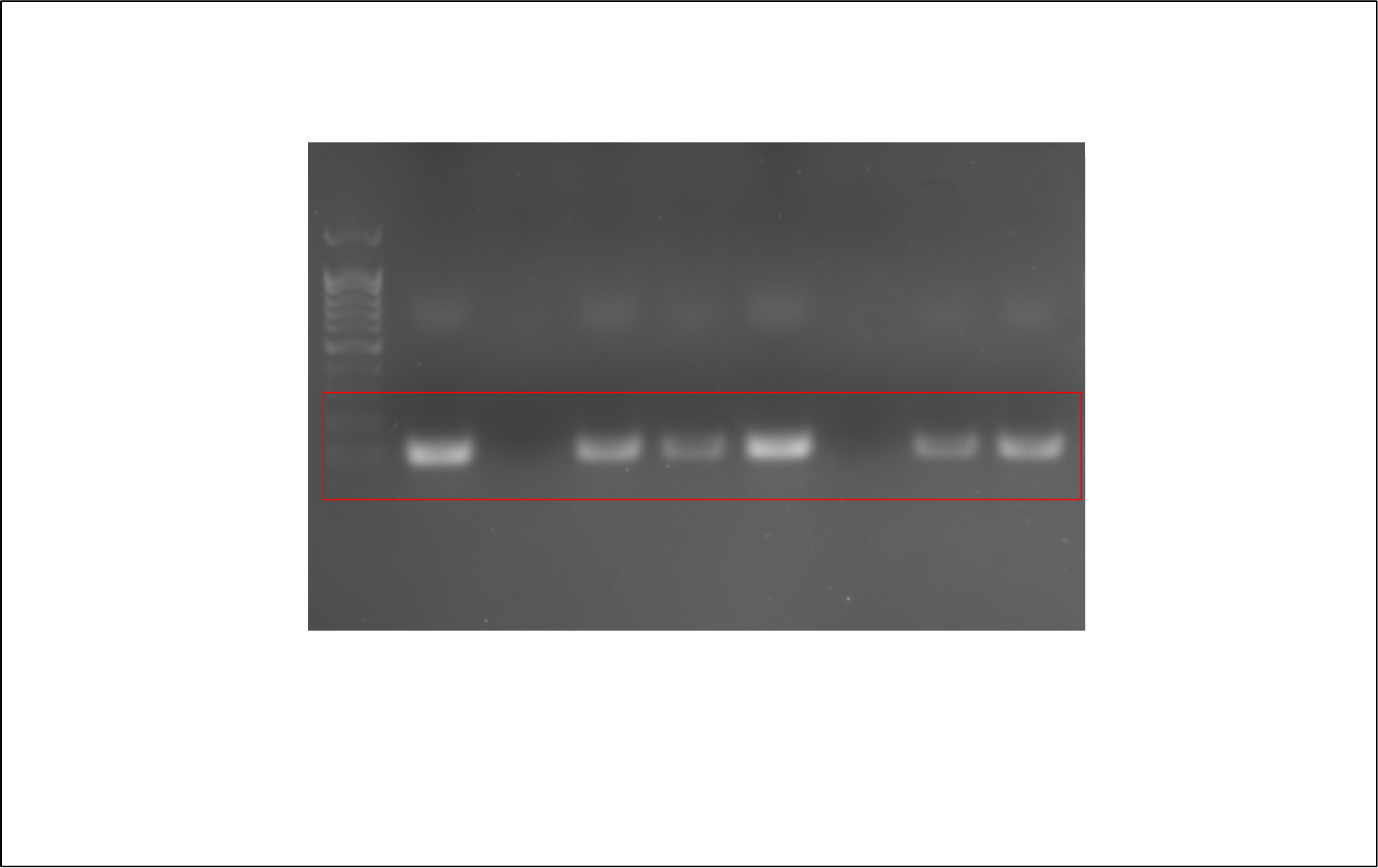

Supplement: Supplementary file 8 — Source data Fig. 6 [file 44319_2024_292_MOESM8_ESM.zip › EMBOR-2024-59294V3-Figure_6_Source_Data-sd/embr202459294-sup-sdatafig6/6G/6G-1.tif]

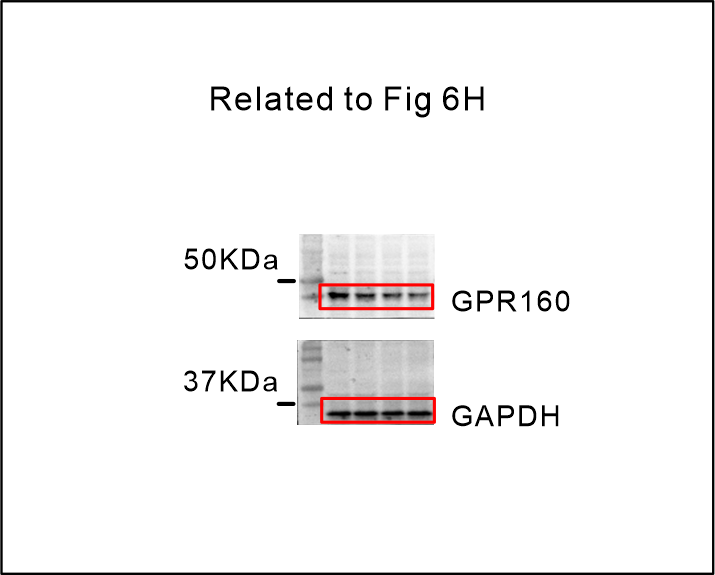

Supplement: Supplementary file 8 — Source data Fig. 6 [file 44319_2024_292_MOESM8_ESM.zip › EMBOR-2024-59294V3-Figure_6_Source_Data-sd/embr202459294-sup-sdatafig6/6H/6H.tif]

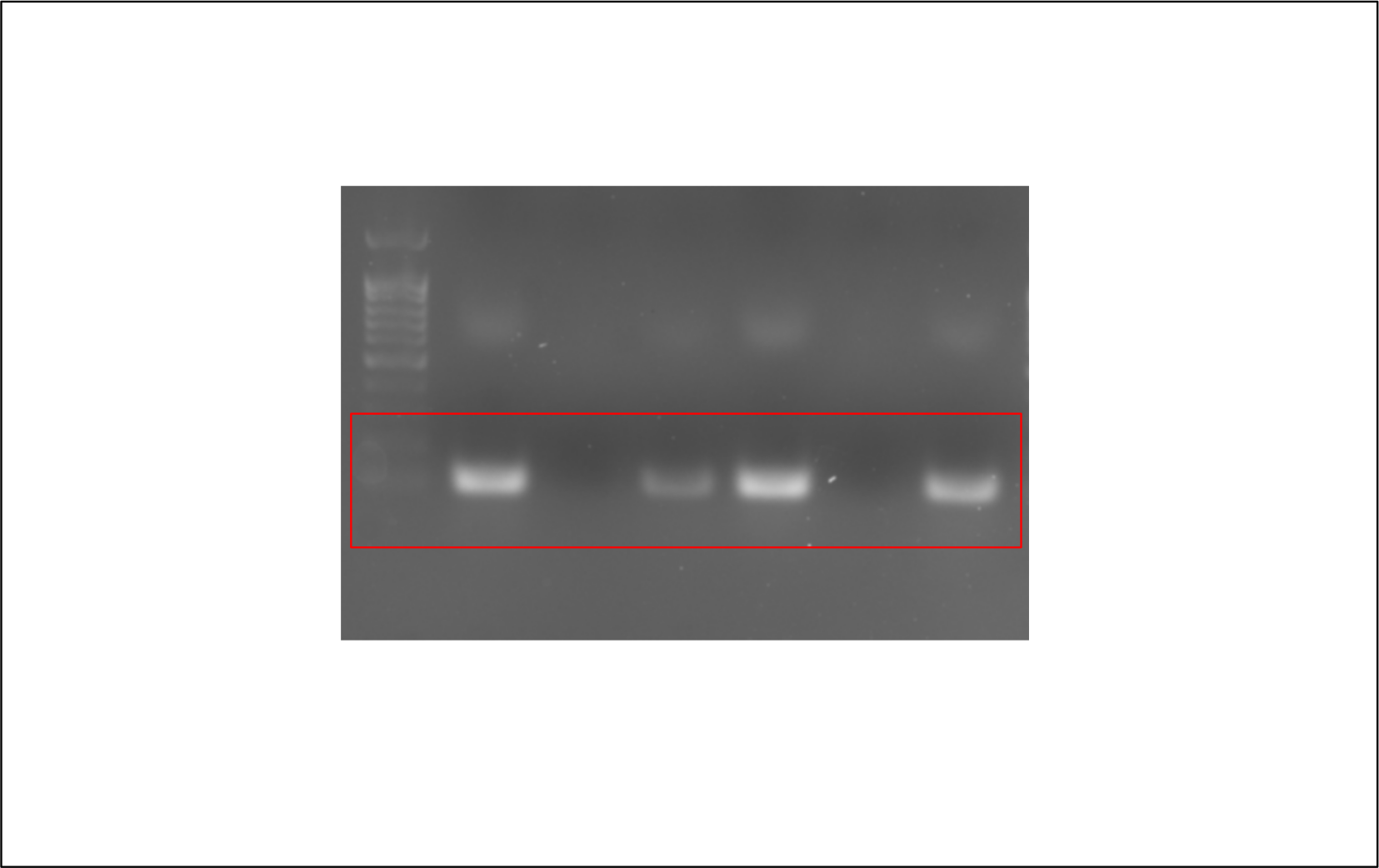

Supplement: Supplementary file 9 — Source data Fig. 7 [file 44319_2024_292_MOESM9_ESM.zip › EMBOR-2024-59294V3-Figure_7_Source_Data-sd/embr202459294-sup-sdatafig7/7D/7D-1.tif]

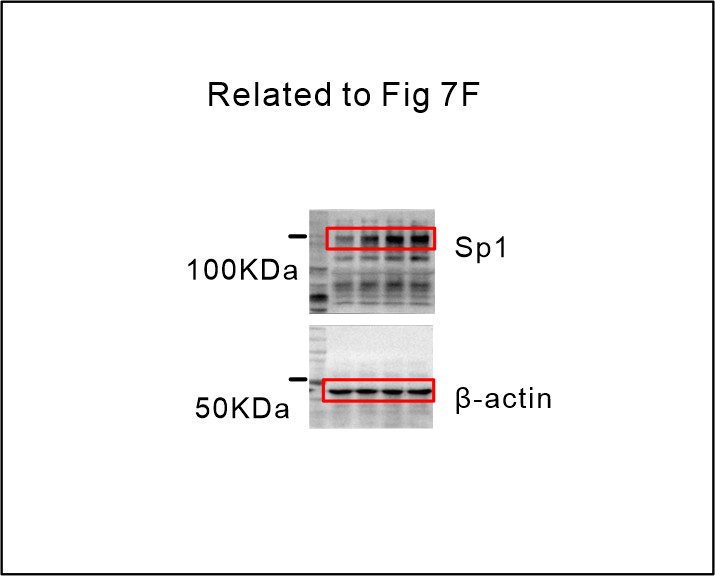

Supplement: Supplementary file 9 — Source data Fig. 7 [file 44319_2024_292_MOESM9_ESM.zip › EMBOR-2024-59294V3-Figure_7_Source_Data-sd/embr202459294-sup-sdatafig7/7F/7F.tif]

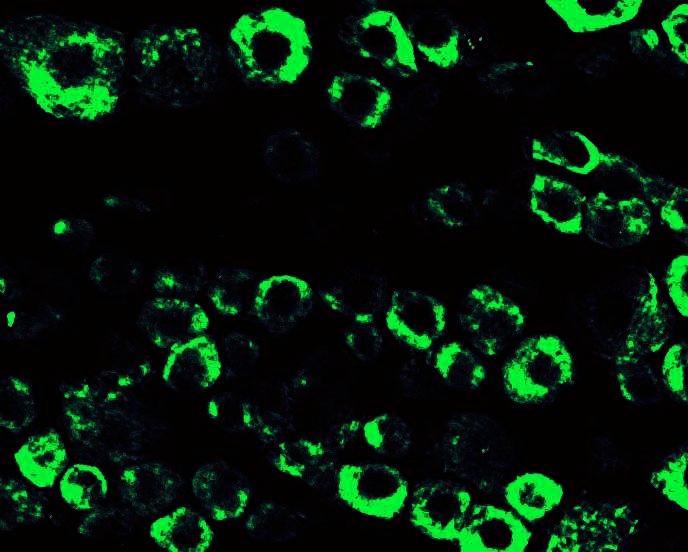

Supplement: Supplementary file 9 — Source data Fig. 7 [file 44319_2024_292_MOESM9_ESM.zip › EMBOR-2024-59294V3-Figure_7_Source_Data-sd/embr202459294-sup-sdatafig7/7G/B1.jpg]

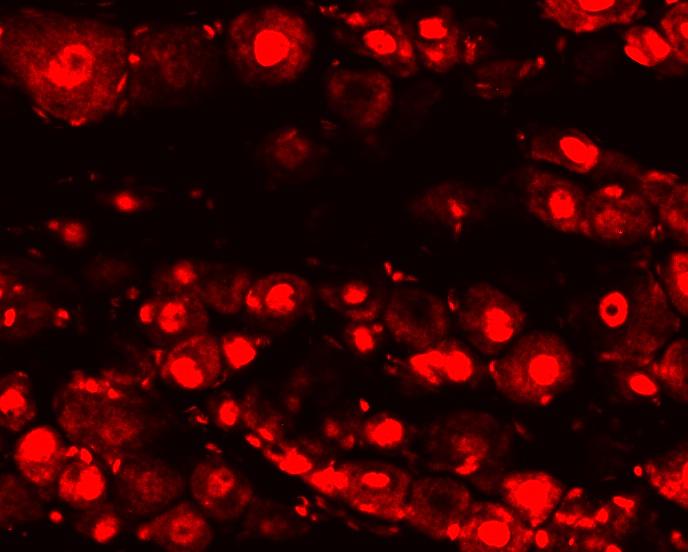

Supplement: Supplementary file 9 — Source data Fig. 7 [file 44319_2024_292_MOESM9_ESM.zip › EMBOR-2024-59294V3-Figure_7_Source_Data-sd/embr202459294-sup-sdatafig7/7G/B2.jpg]

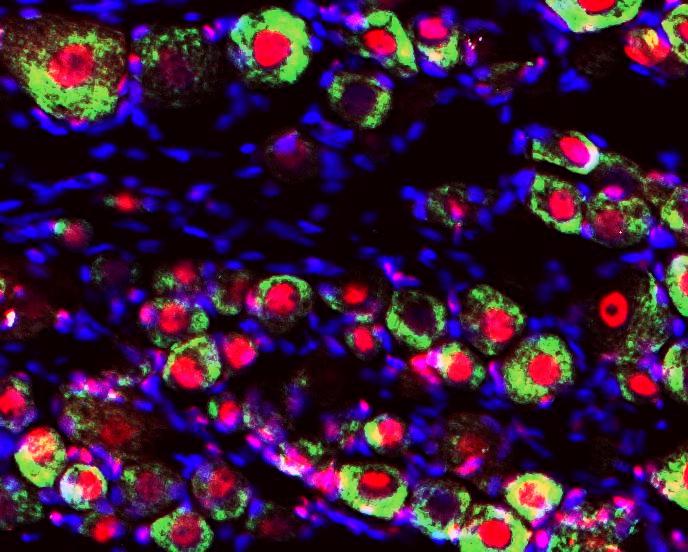

Supplement: Supplementary file 9 — Source data Fig. 7 [file 44319_2024_292_MOESM9_ESM.zip › EMBOR-2024-59294V3-Figure_7_Source_Data-sd/embr202459294-sup-sdatafig7/7G/B3.jpg]

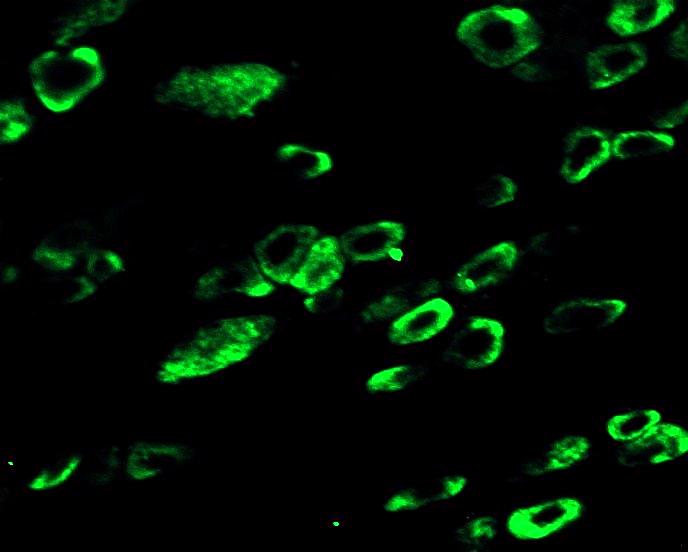

Supplement: Supplementary file 9 — Source data Fig. 7 [file 44319_2024_292_MOESM9_ESM.zip › EMBOR-2024-59294V3-Figure_7_Source_Data-sd/embr202459294-sup-sdatafig7/7G/S1.jpg]

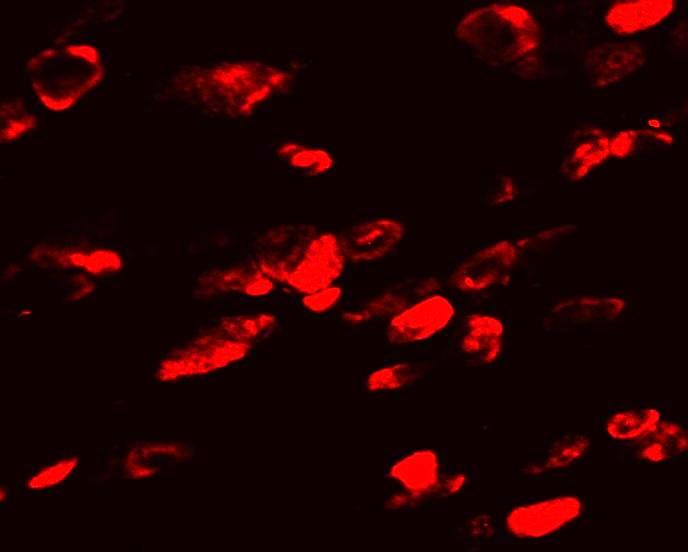

Supplement: Supplementary file 9 — Source data Fig. 7 [file 44319_2024_292_MOESM9_ESM.zip › EMBOR-2024-59294V3-Figure_7_Source_Data-sd/embr202459294-sup-sdatafig7/7G/S2.jpg]

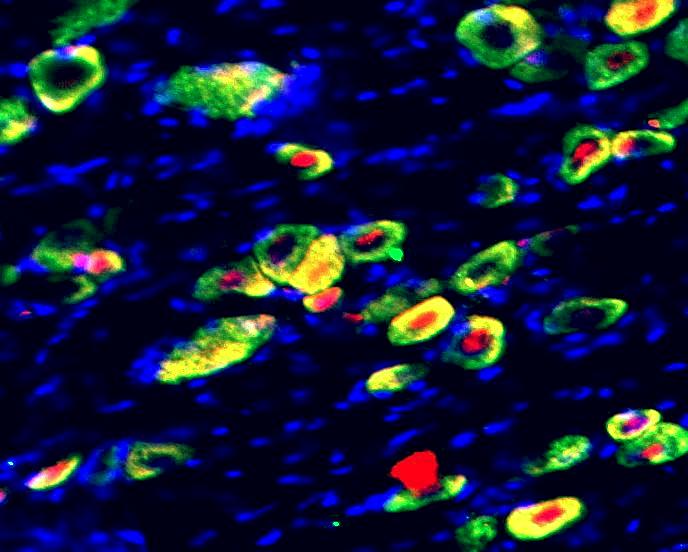

Supplement: Supplementary file 9 — Source data Fig. 7 [file 44319_2024_292_MOESM9_ESM.zip › EMBOR-2024-59294V3-Figure_7_Source_Data-sd/embr202459294-sup-sdatafig7/7G/S3.jpg]

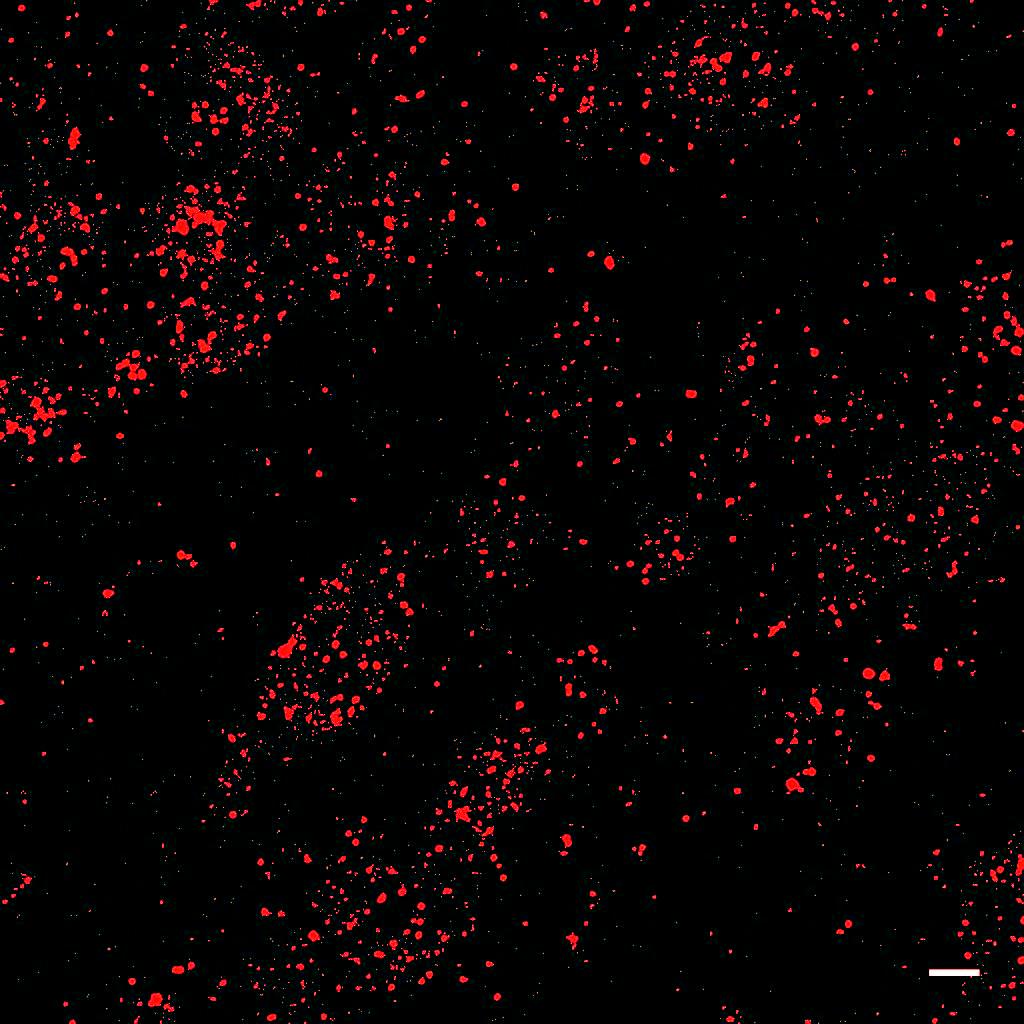

Supplement: Supplementary file 9 — Source data Fig. 7 [file 44319_2024_292_MOESM9_ESM.zip › EMBOR-2024-59294V3-Figure_7_Source_Data-sd/embr202459294-sup-sdatafig7/7I/1.jpg]

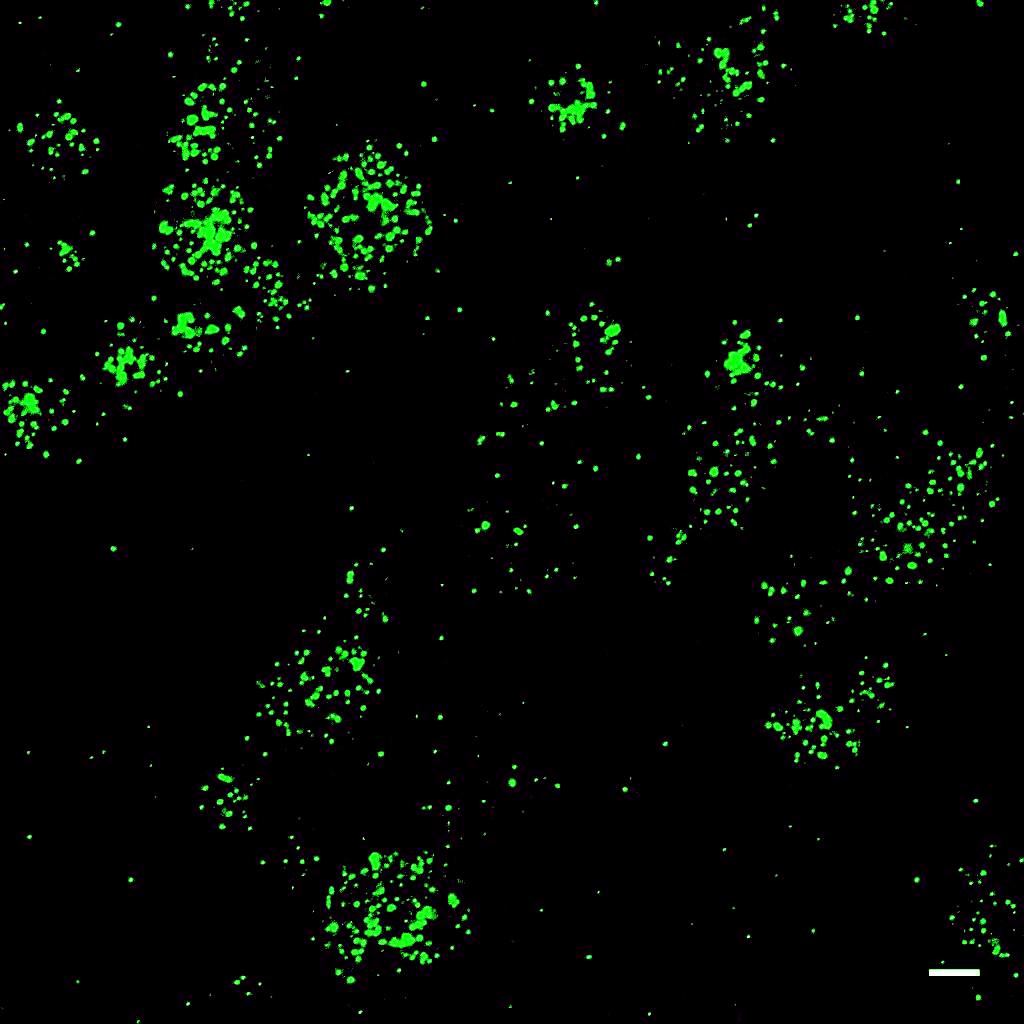

Supplement: Supplementary file 9 — Source data Fig. 7 [file 44319_2024_292_MOESM9_ESM.zip › EMBOR-2024-59294V3-Figure_7_Source_Data-sd/embr202459294-sup-sdatafig7/7I/2.jpg]

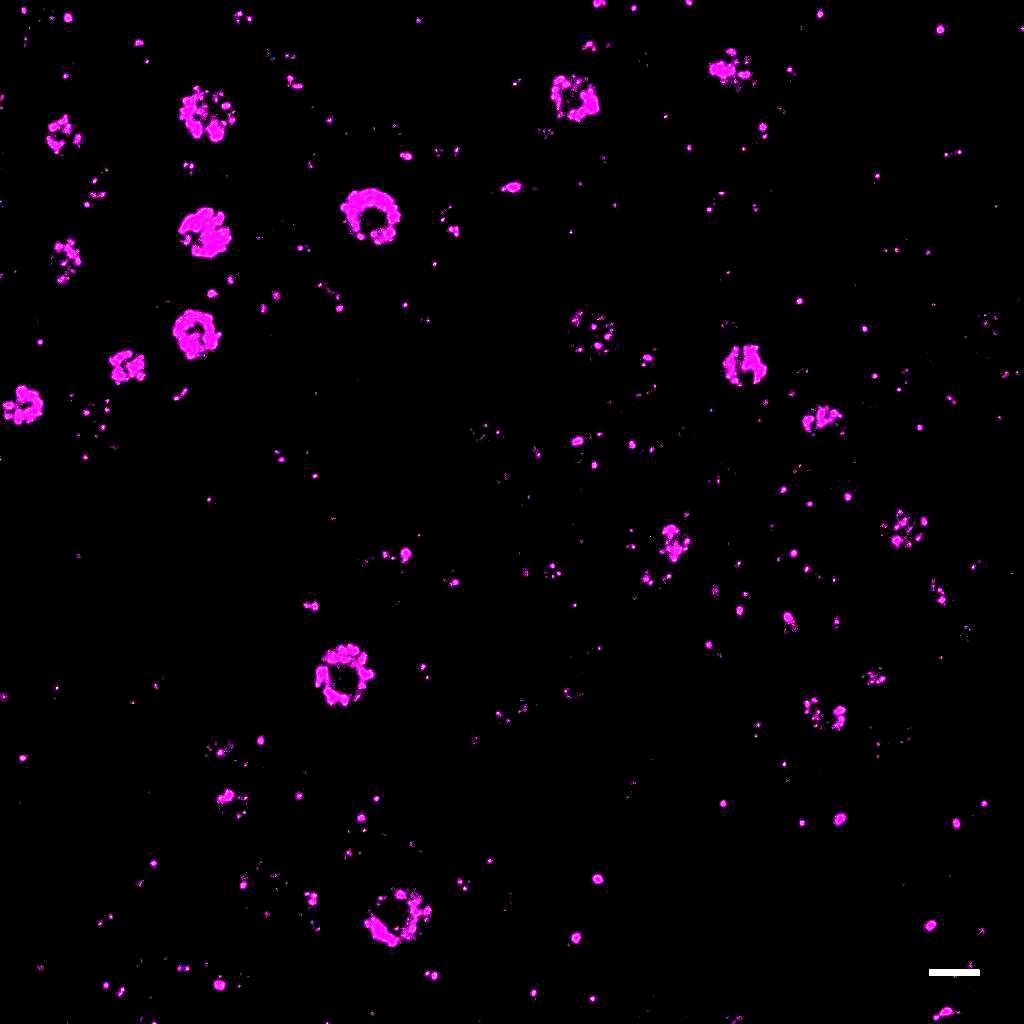

Supplement: Supplementary file 9 — Source data Fig. 7 [file 44319_2024_292_MOESM9_ESM.zip › EMBOR-2024-59294V3-Figure_7_Source_Data-sd/embr202459294-sup-sdatafig7/7I/3.jpg]

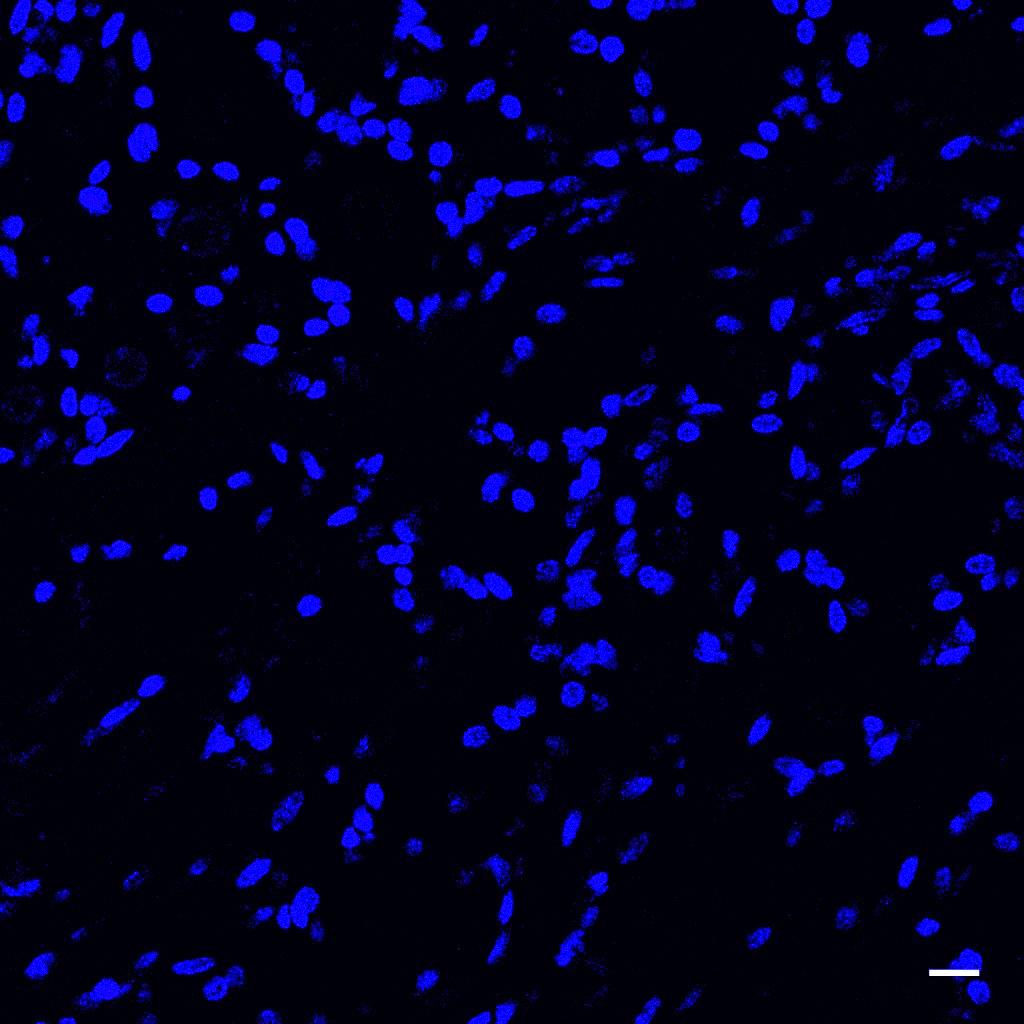

Supplement: Supplementary file 9 — Source data Fig. 7 [file 44319_2024_292_MOESM9_ESM.zip › EMBOR-2024-59294V3-Figure_7_Source_Data-sd/embr202459294-sup-sdatafig7/7I/4.jpg]

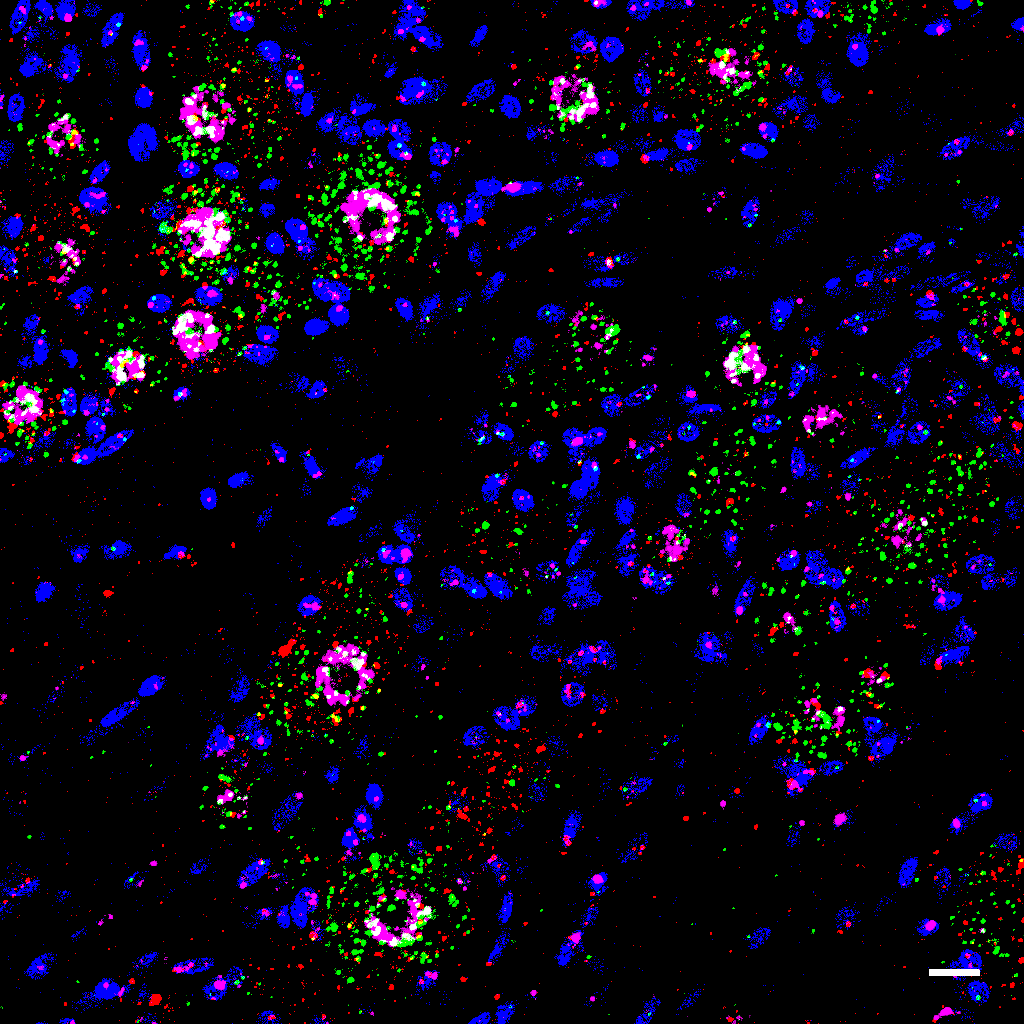

Supplement: Supplementary file 9 — Source data Fig. 7 [file 44319_2024_292_MOESM9_ESM.zip › EMBOR-2024-59294V3-Figure_7_Source_Data-sd/embr202459294-sup-sdatafig7/7I/5.tif]

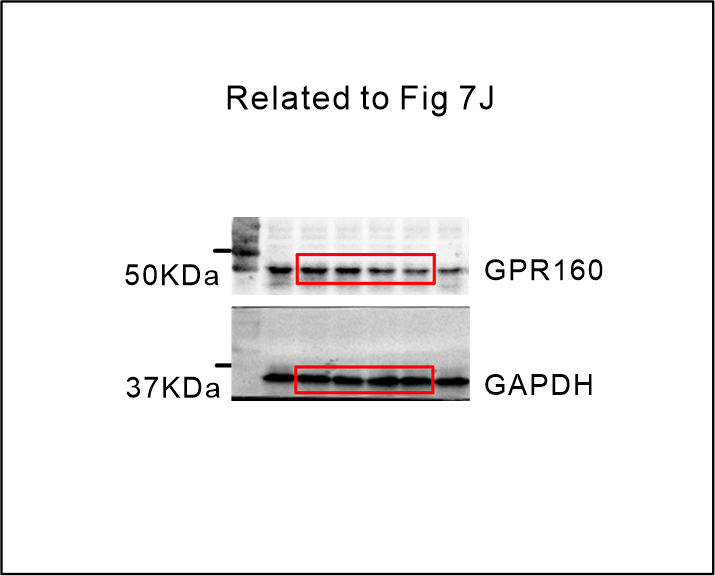

Supplement: Supplementary file 9 — Source data Fig. 7 [file 44319_2024_292_MOESM9_ESM.zip › EMBOR-2024-59294V3-Figure_7_Source_Data-sd/embr202459294-sup-sdatafig7/7J/7J-1.tif]

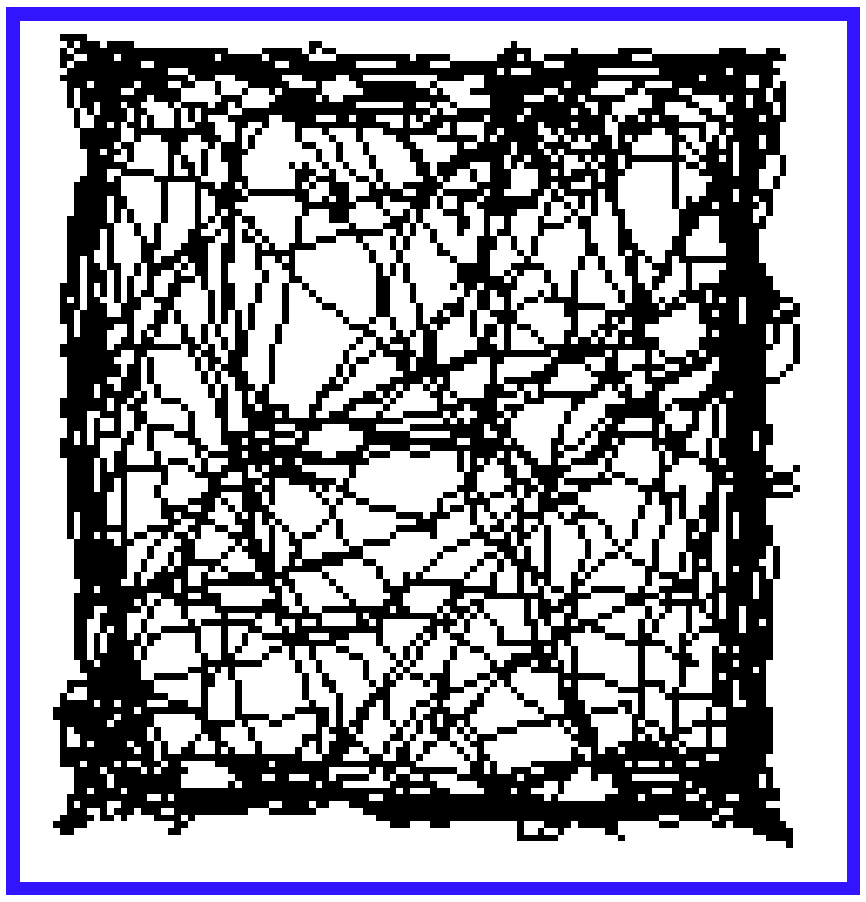

Supplement: Supplementary file 10 — EV and Appendix Figures Source Data [file 44319_2024_292_MOESM10_ESM.zip › EMBOR-2024-59294V3-Figure_EV5_Source_Data-sd/Figure EV5/EV5H/EV5H-1.png]

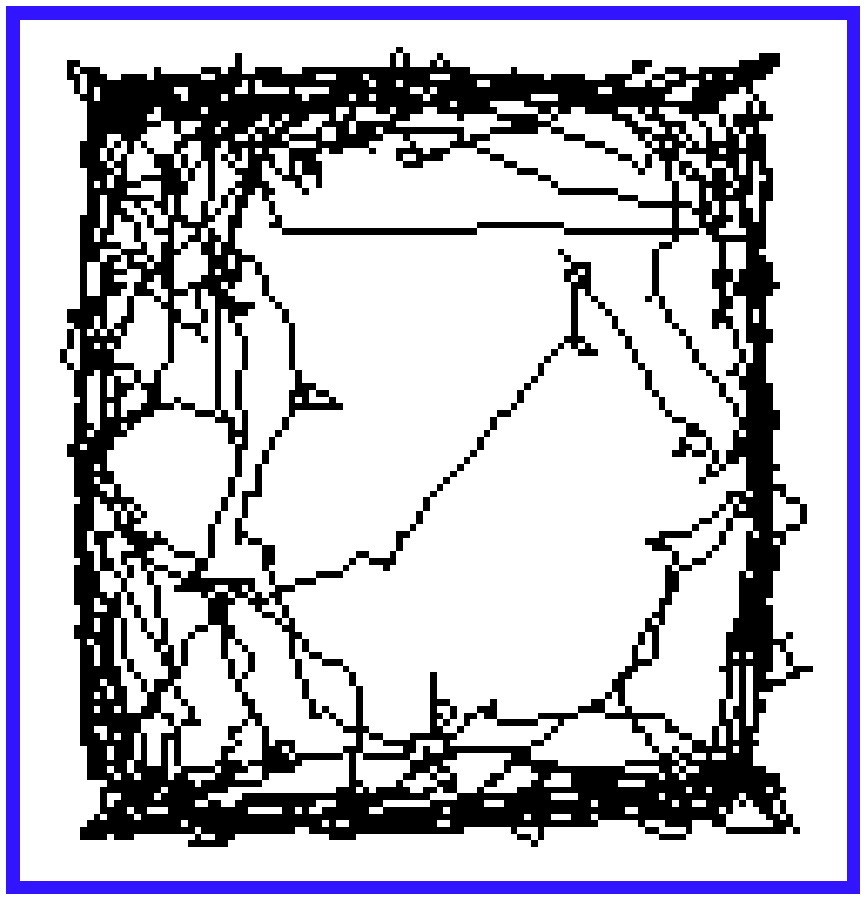

Supplement: Supplementary file 10 — EV and Appendix Figures Source Data [file 44319_2024_292_MOESM10_ESM.zip › EMBOR-2024-59294V3-Figure_EV5_Source_Data-sd/Figure EV5/EV5H/EV5H-2.png]

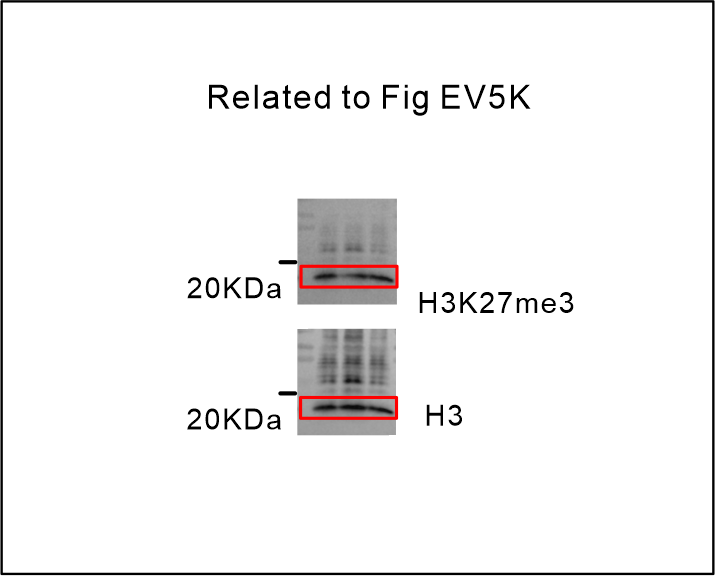

Supplement: Supplementary file 10 — EV and Appendix Figures Source Data [file 44319_2024_292_MOESM10_ESM.zip › EMBOR-2024-59294V3-Figure_EV5_Source_Data-sd/Figure EV5/EV5K-L/EV5K.tif]

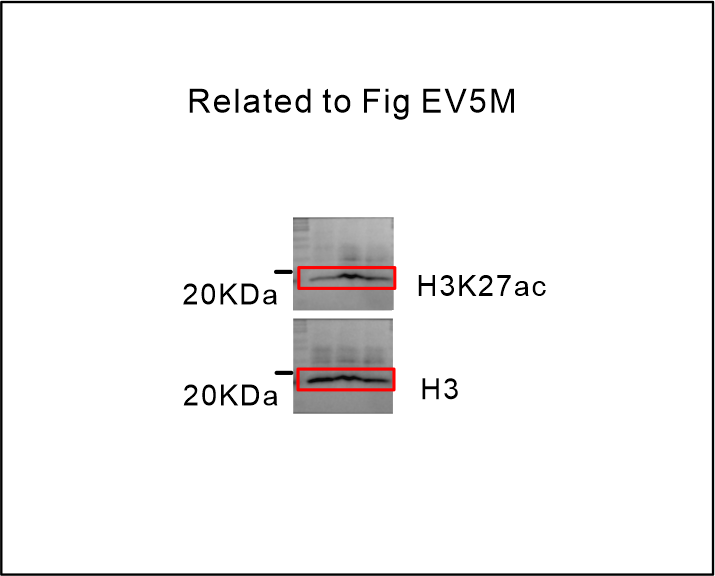

Supplement: Supplementary file 10 — EV and Appendix Figures Source Data [file 44319_2024_292_MOESM10_ESM.zip › EMBOR-2024-59294V3-Figure_EV5_Source_Data-sd/Figure EV5/EV5M-N/EV5M.tif]

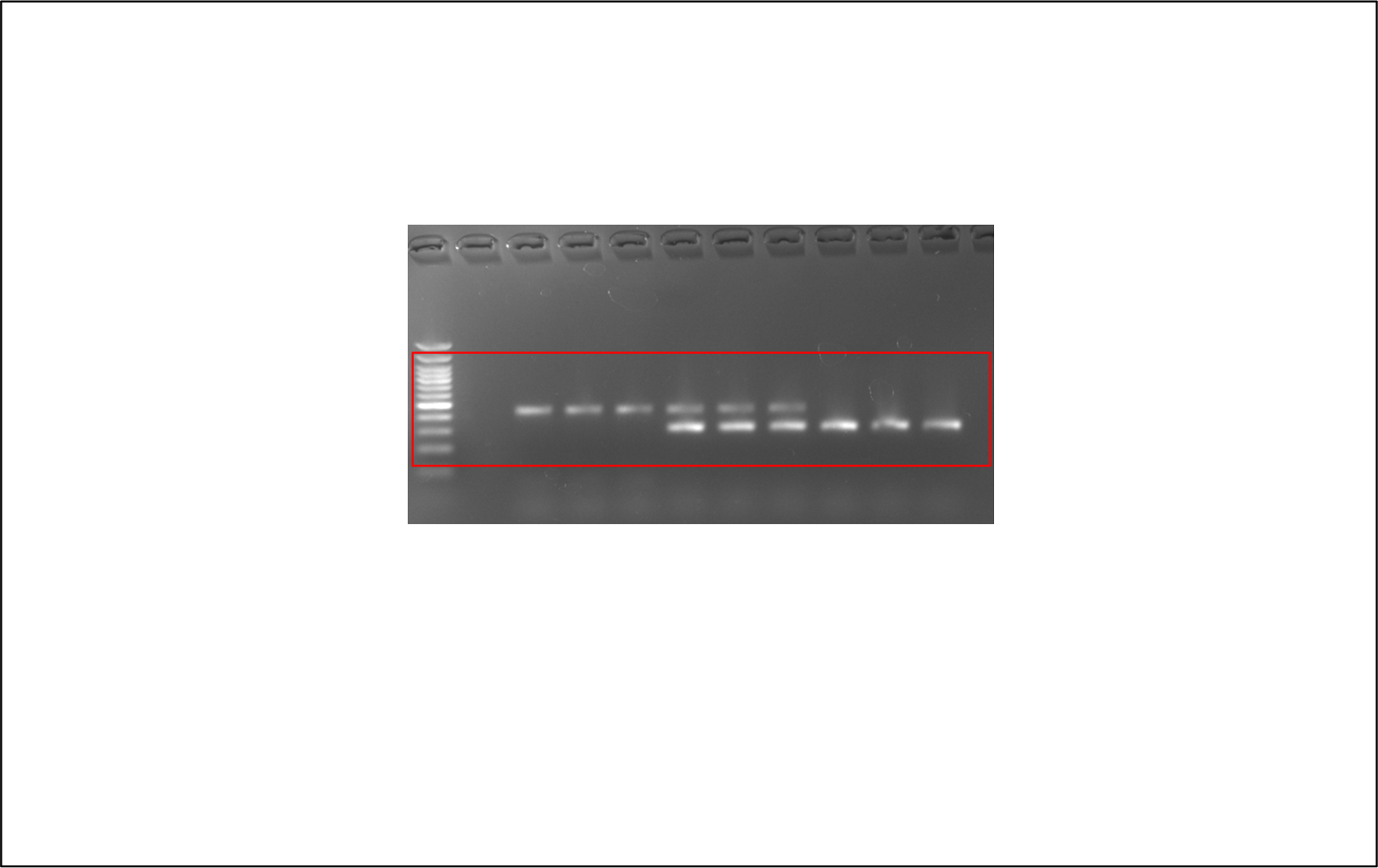

Supplement: Supplementary file 10 — EV and Appendix Figures Source Data [file 44319_2024_292_MOESM10_ESM.zip › EMBOR-2024-59294V3-Figure_S1_Source_Data-sd/Appendix Figure S1/S1B/S1B.tif]

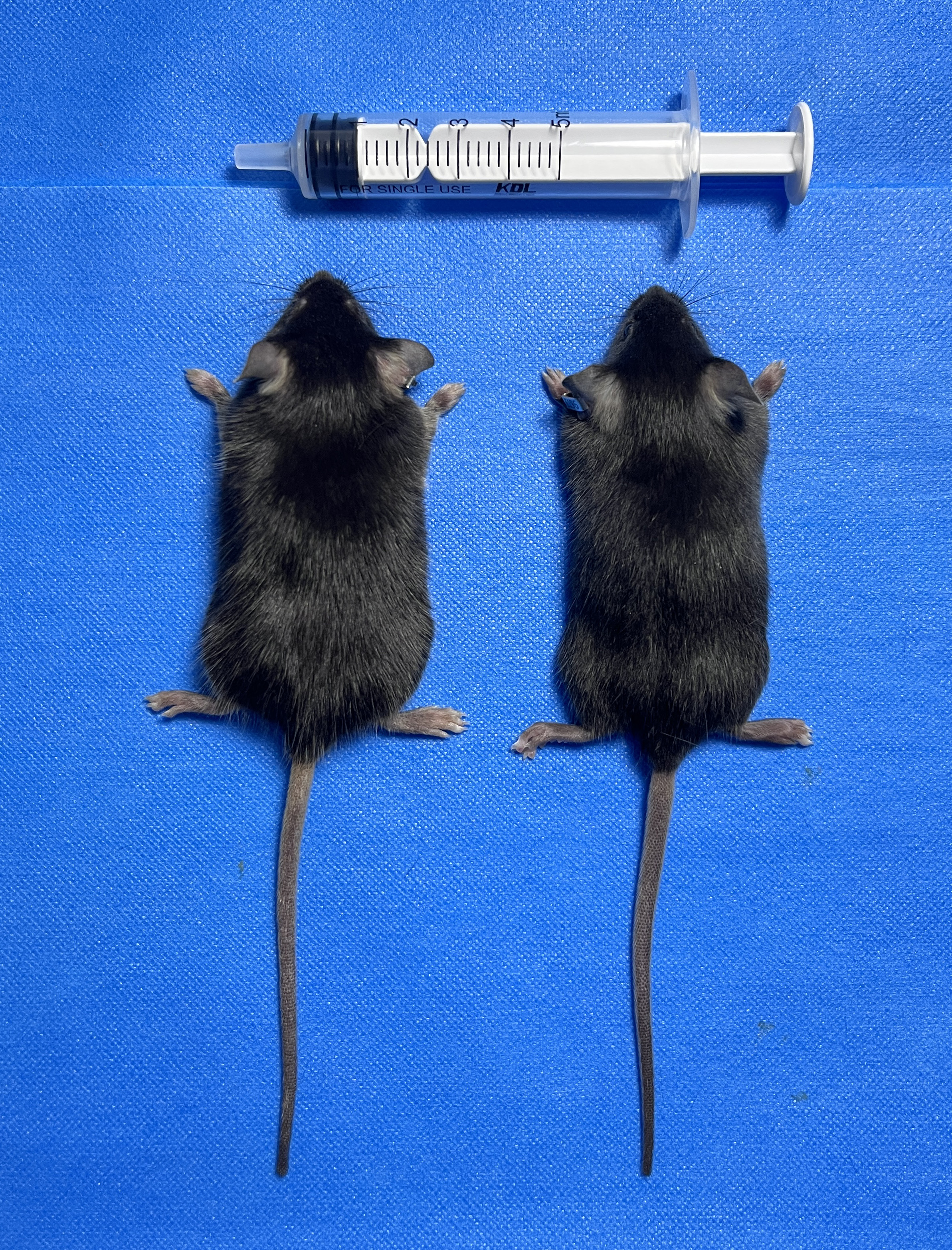

Supplement: Supplementary file 10 — EV and Appendix Figures Source Data [file 44319_2024_292_MOESM10_ESM.zip › EMBOR-2024-59294V3-Figure_S1_Source_Data-sd/Appendix Figure S1/S1C/S1C-1.jpg]

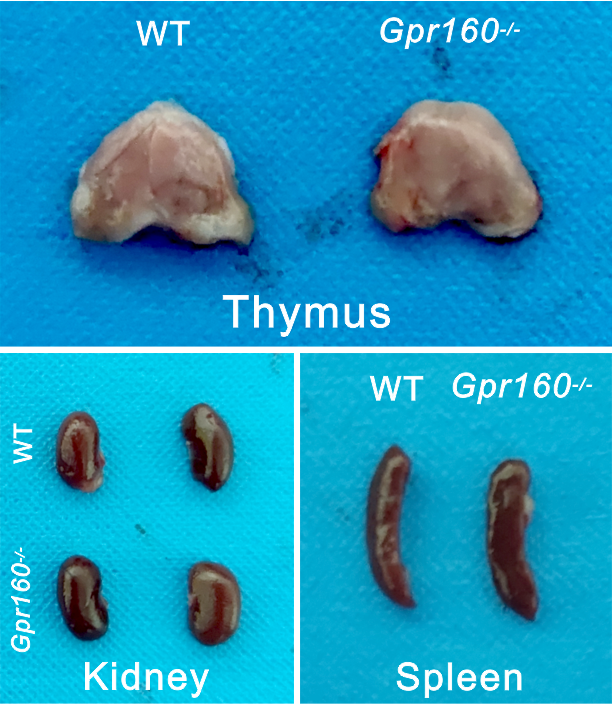

Supplement: Supplementary file 10 — EV and Appendix Figures Source Data [file 44319_2024_292_MOESM10_ESM.zip › EMBOR-2024-59294V3-Figure_S1_Source_Data-sd/Appendix Figure S1/S1C/S1C-2.tif]

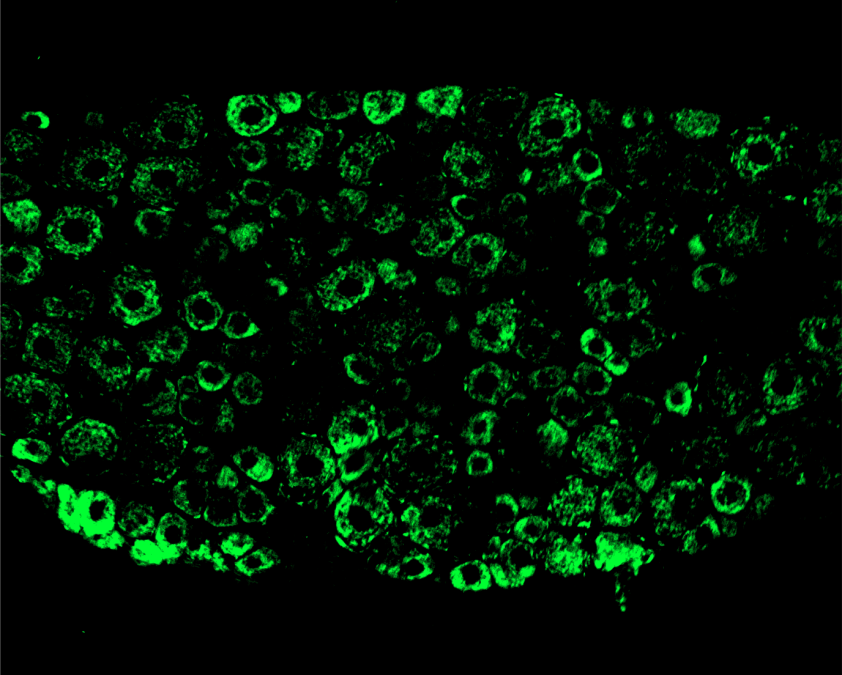

Supplement: Supplementary file 10 — EV and Appendix Figures Source Data [file 44319_2024_292_MOESM10_ESM.zip › EMBOR-2024-59294V3-Figure_S1_Source_Data-sd/Appendix Figure S1/S1D/S1D-1.tif]

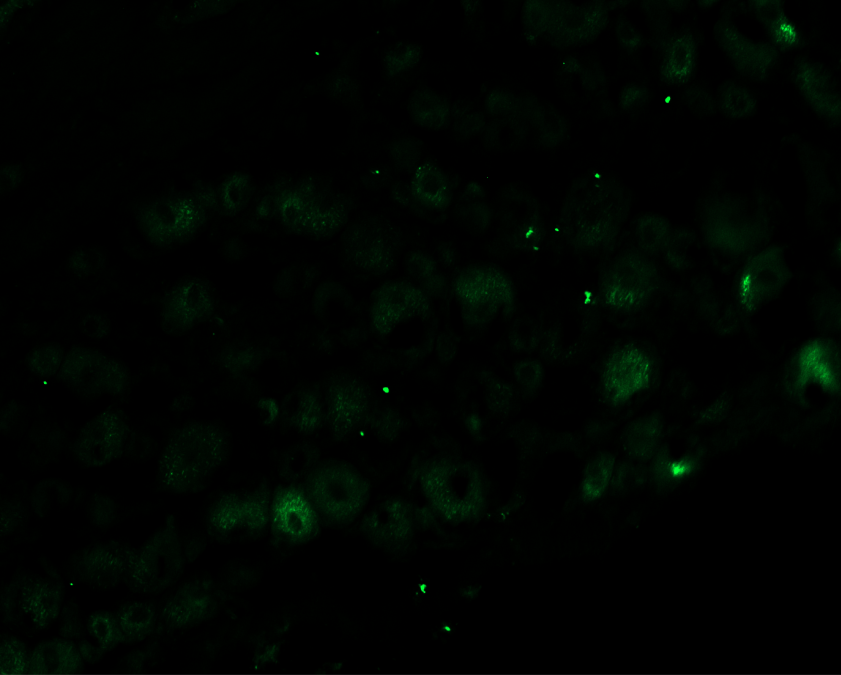

Supplement: Supplementary file 10 — EV and Appendix Figures Source Data [file 44319_2024_292_MOESM10_ESM.zip › EMBOR-2024-59294V3-Figure_S1_Source_Data-sd/Appendix Figure S1/S1D/S1D-2.tif]

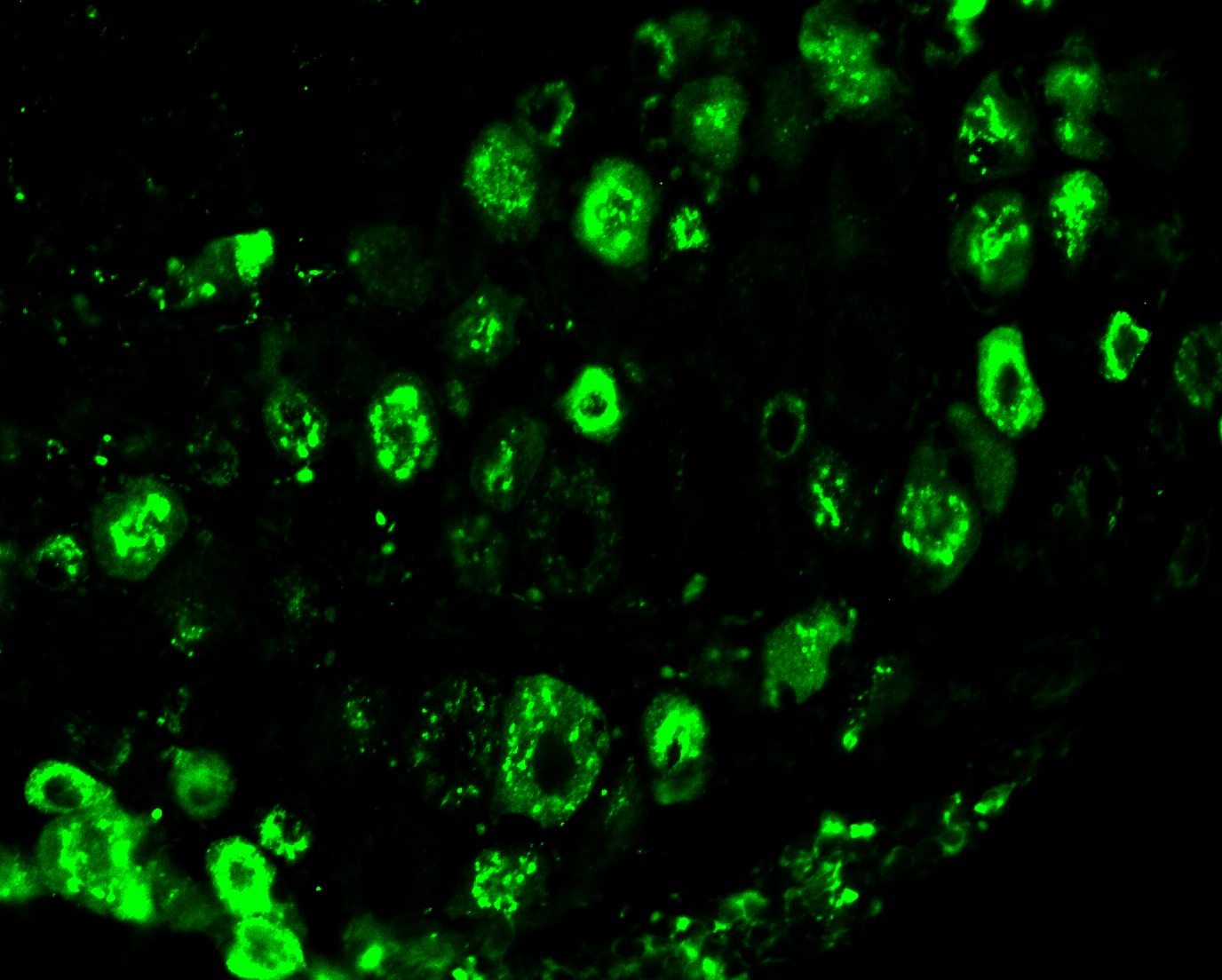

Supplement: Supplementary file 10 — EV and Appendix Figures Source Data [file 44319_2024_292_MOESM10_ESM.zip › EMBOR-2024-59294V3-Figure_S1_Source_Data-sd/Appendix Figure S1/S1E/S1E-1.jpg]

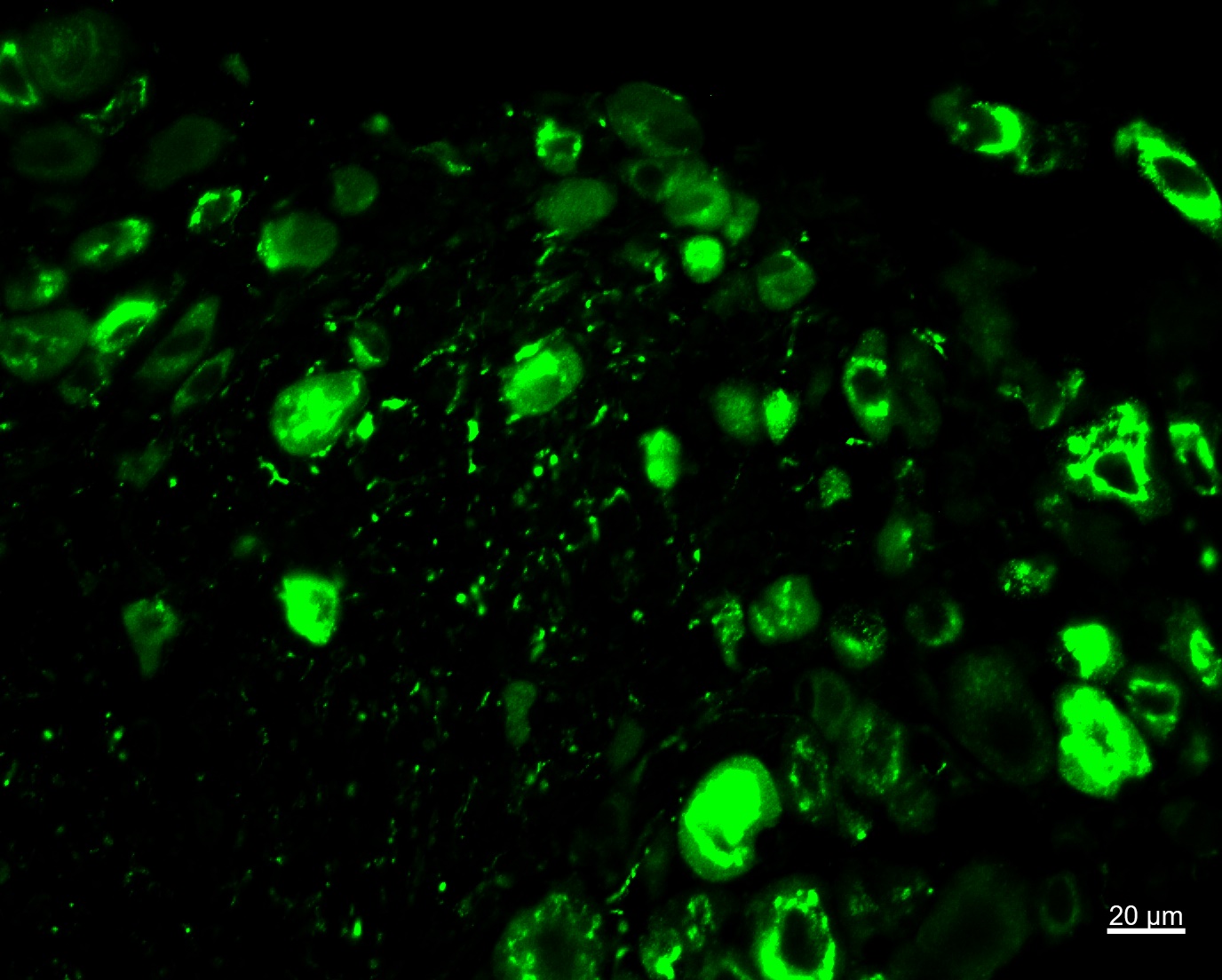

Supplement: Supplementary file 10 — EV and Appendix Figures Source Data [file 44319_2024_292_MOESM10_ESM.zip › EMBOR-2024-59294V3-Figure_S1_Source_Data-sd/Appendix Figure S1/S1E/S1E-2.jpg]

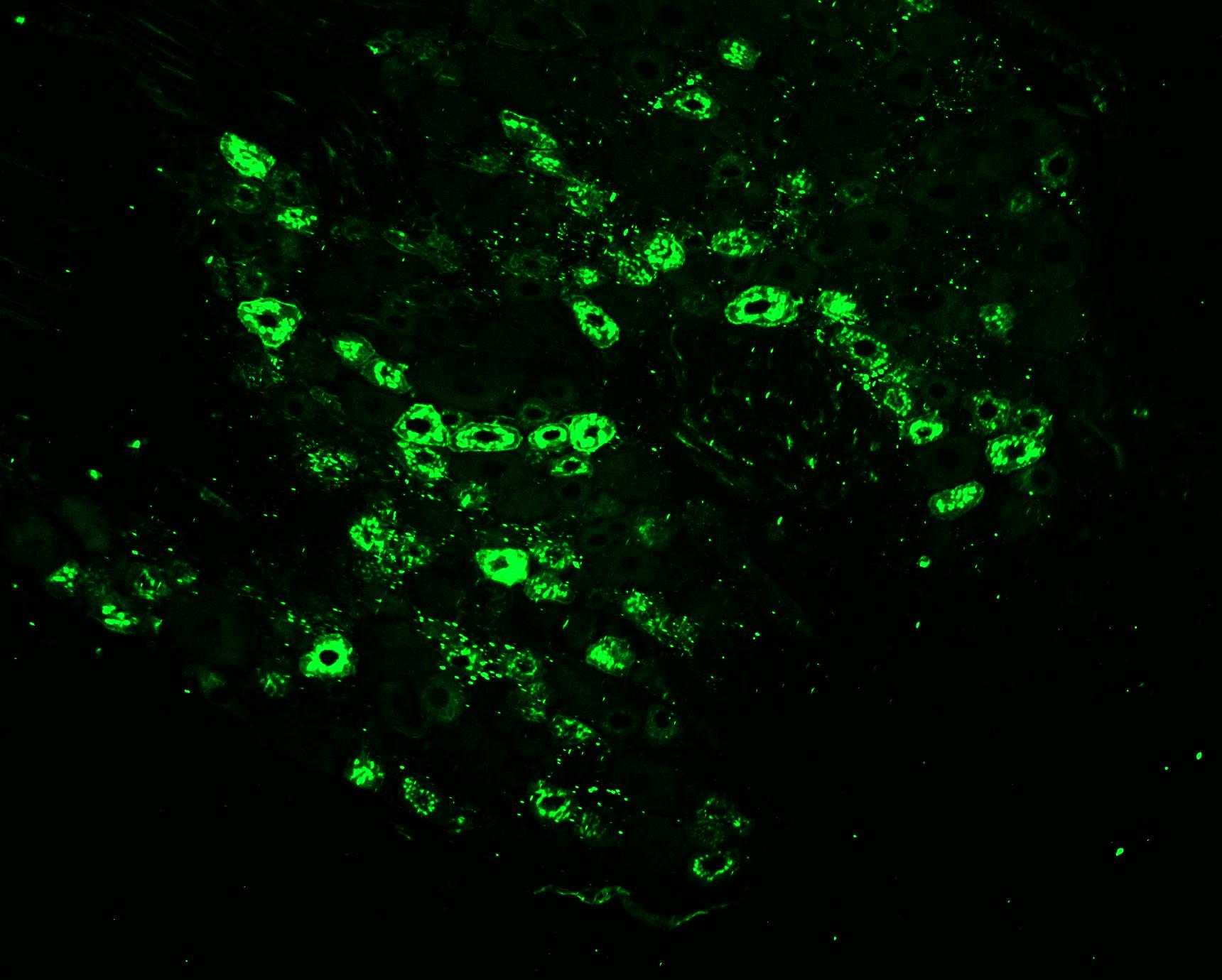

Supplement: Supplementary file 10 — EV and Appendix Figures Source Data [file 44319_2024_292_MOESM10_ESM.zip › EMBOR-2024-59294V3-Figure_S1_Source_Data-sd/Appendix Figure S1/S1F/S1F-1.jpg]

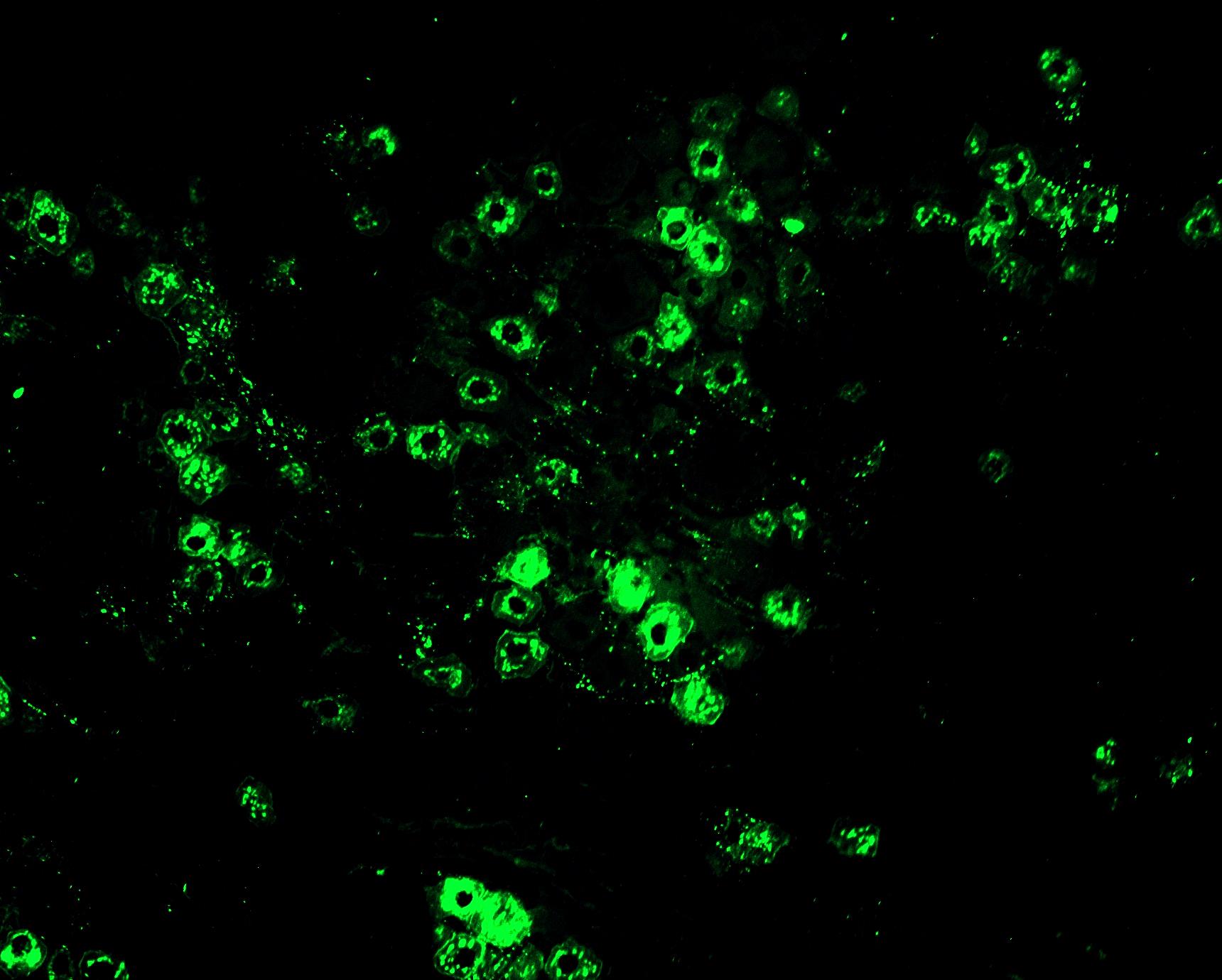

Supplement: Supplementary file 10 — EV and Appendix Figures Source Data [file 44319_2024_292_MOESM10_ESM.zip › EMBOR-2024-59294V3-Figure_S1_Source_Data-sd/Appendix Figure S1/S1F/S1F-2.jpg]

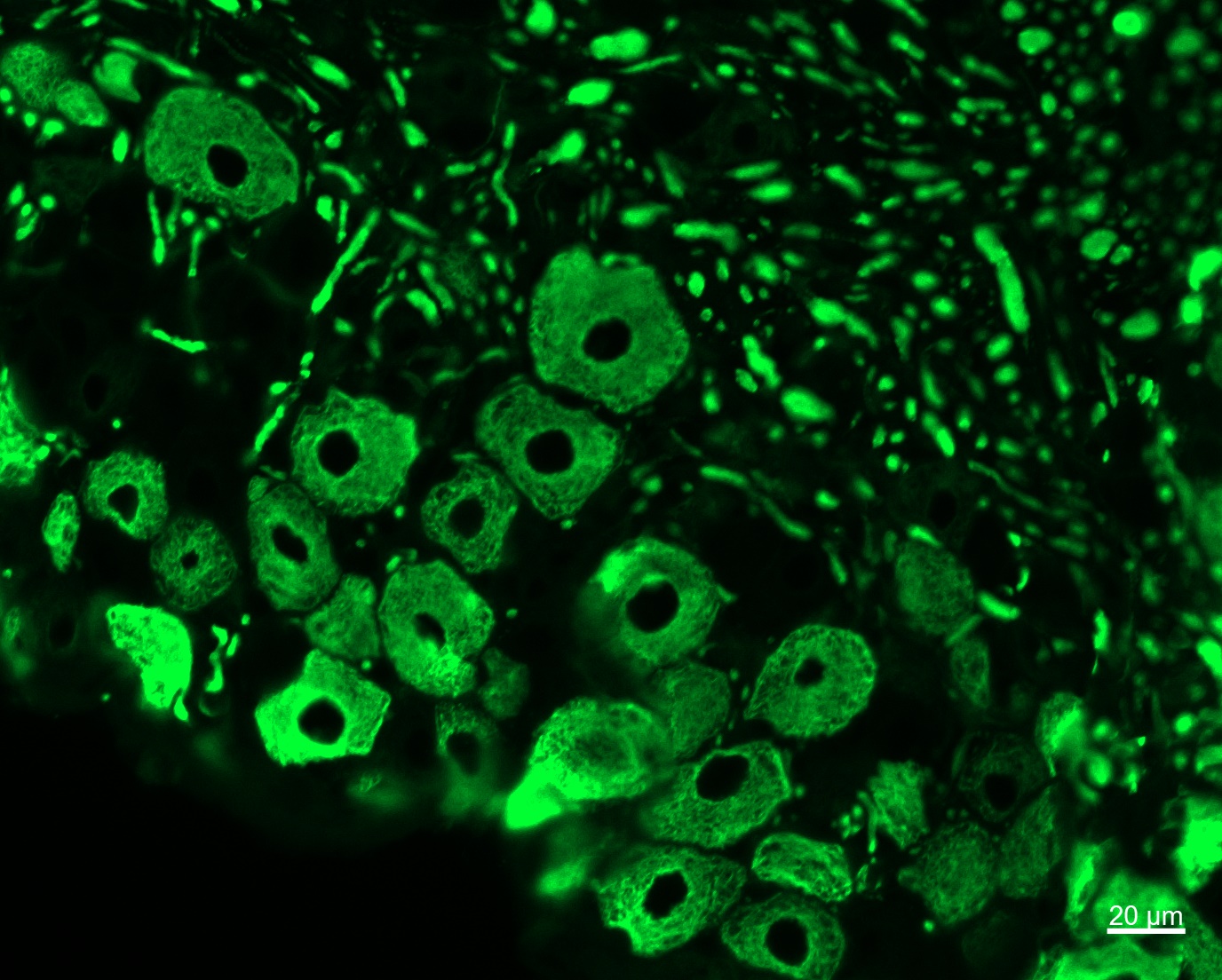

Supplement: Supplementary file 10 — EV and Appendix Figures Source Data [file 44319_2024_292_MOESM10_ESM.zip › EMBOR-2024-59294V3-Figure_S1_Source_Data-sd/Appendix Figure S1/S1G/S1G-1.jpg]

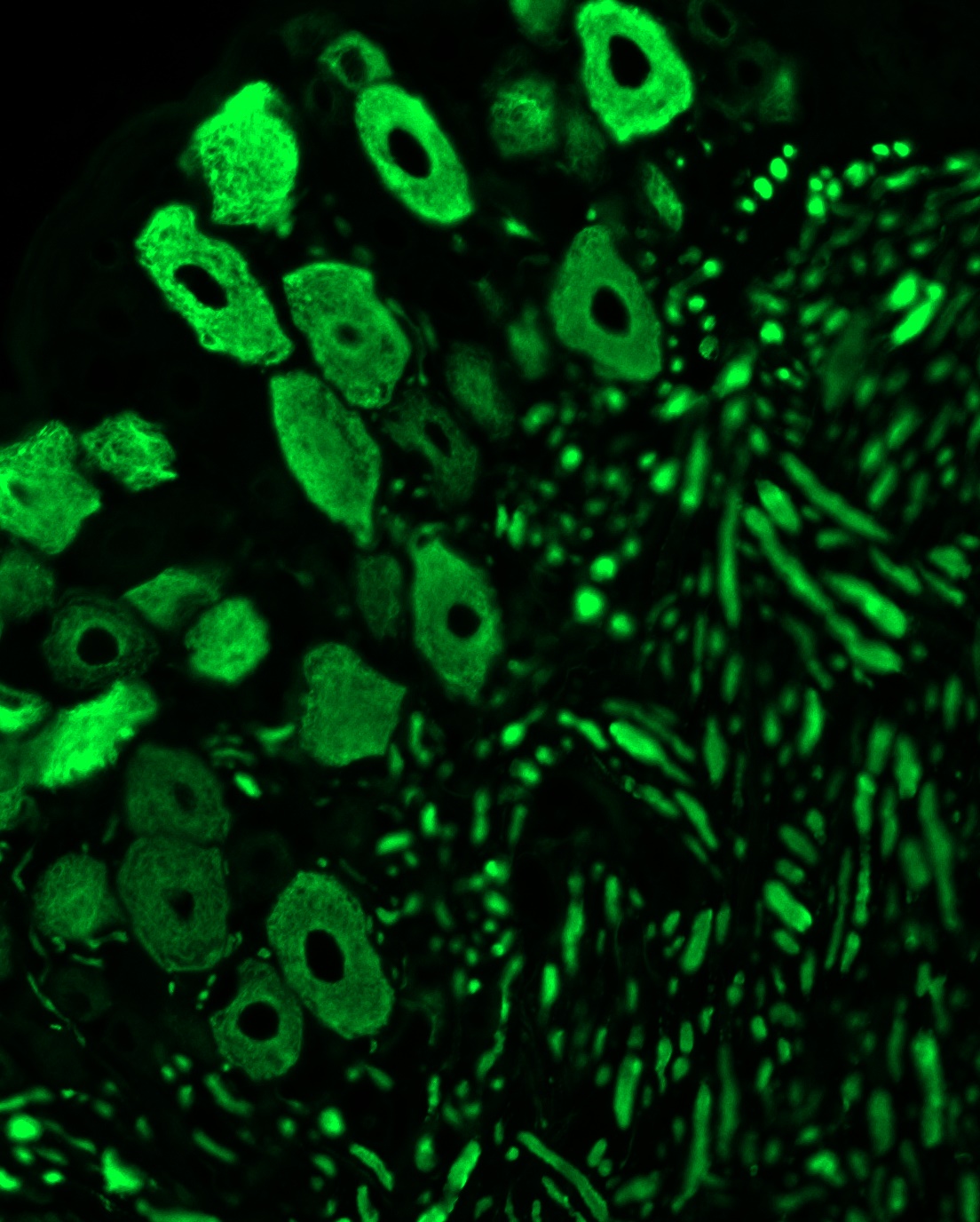

Supplement: Supplementary file 10 — EV and Appendix Figures Source Data [file 44319_2024_292_MOESM10_ESM.zip › EMBOR-2024-59294V3-Figure_S1_Source_Data-sd/Appendix Figure S1/S1G/S1G-2.jpg]

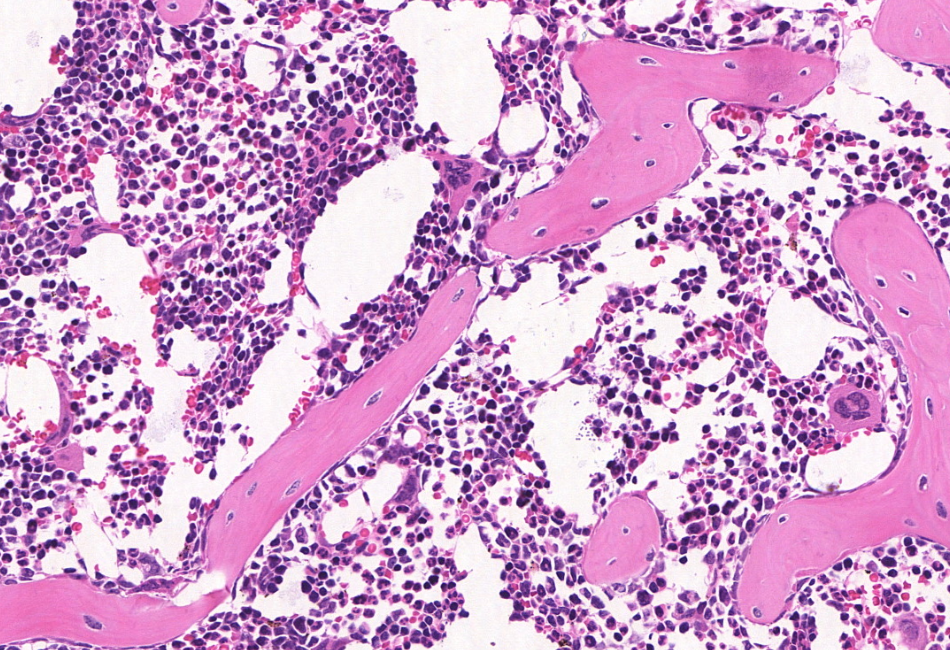

Supplement: Supplementary file 10 — EV and Appendix Figures Source Data [file 44319_2024_292_MOESM10_ESM.zip › EMBOR-2024-59294V3-Figure_S2_Source_Data-sd/Appendix Figure S2/S2A/S2A-1.png]

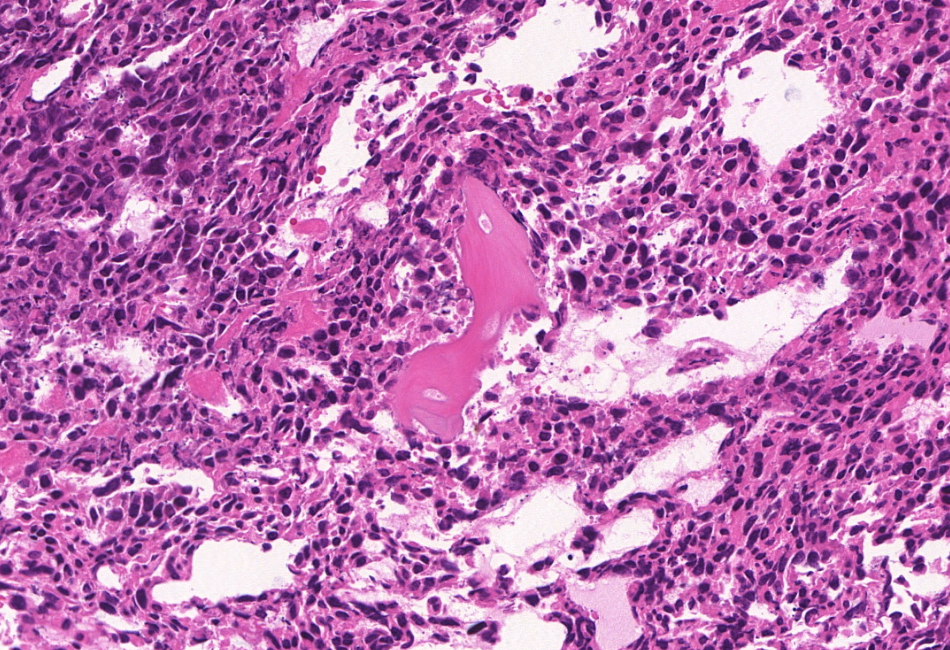

Supplement: Supplementary file 10 — EV and Appendix Figures Source Data [file 44319_2024_292_MOESM10_ESM.zip › EMBOR-2024-59294V3-Figure_S2_Source_Data-sd/Appendix Figure S2/S2A/S2A-2.png]

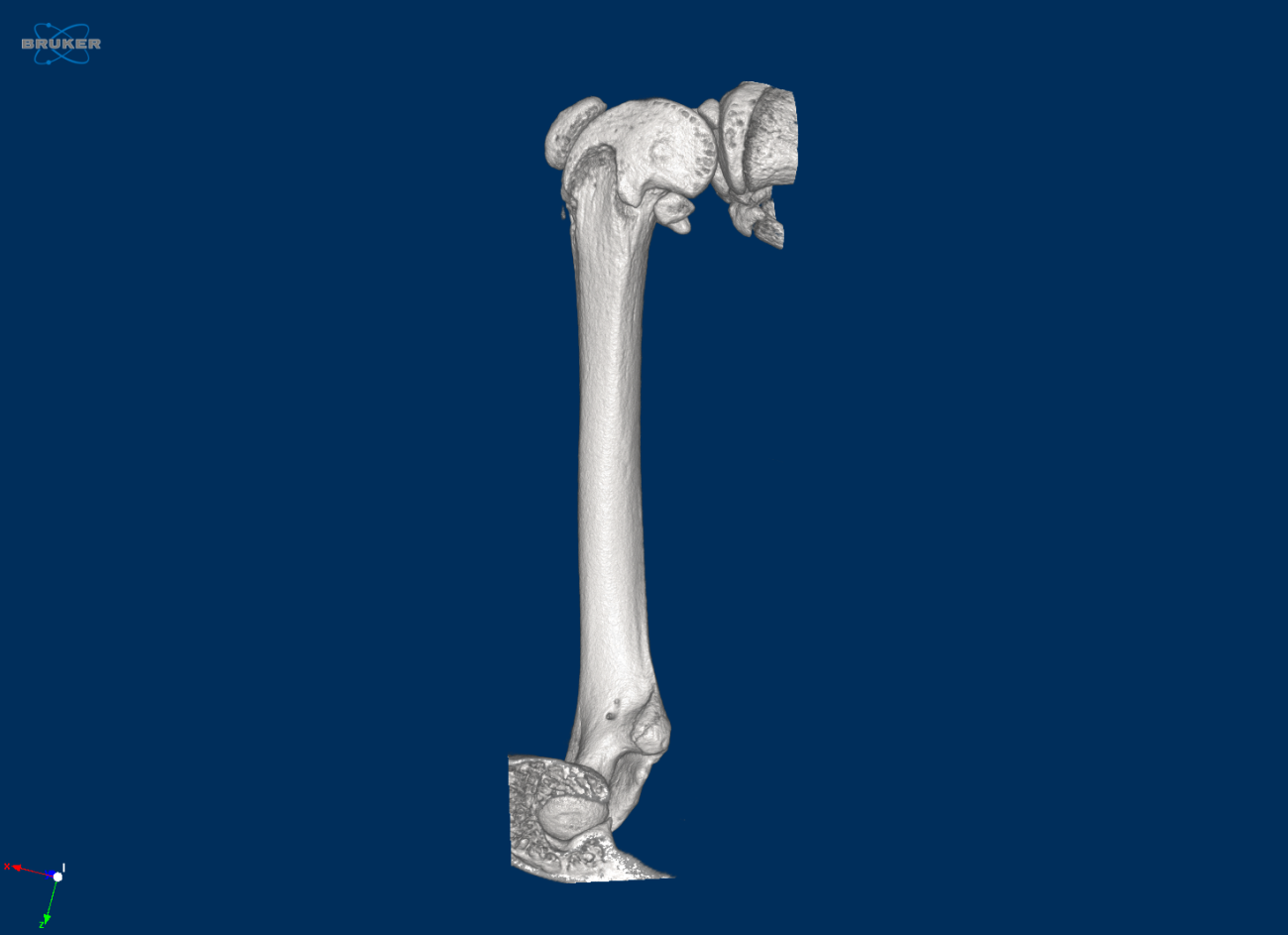

Supplement: Supplementary file 10 — EV and Appendix Figures Source Data [file 44319_2024_292_MOESM10_ESM.zip › EMBOR-2024-59294V3-Figure_S2_Source_Data-sd/Appendix Figure S2/S2B/S2B-1.tif]

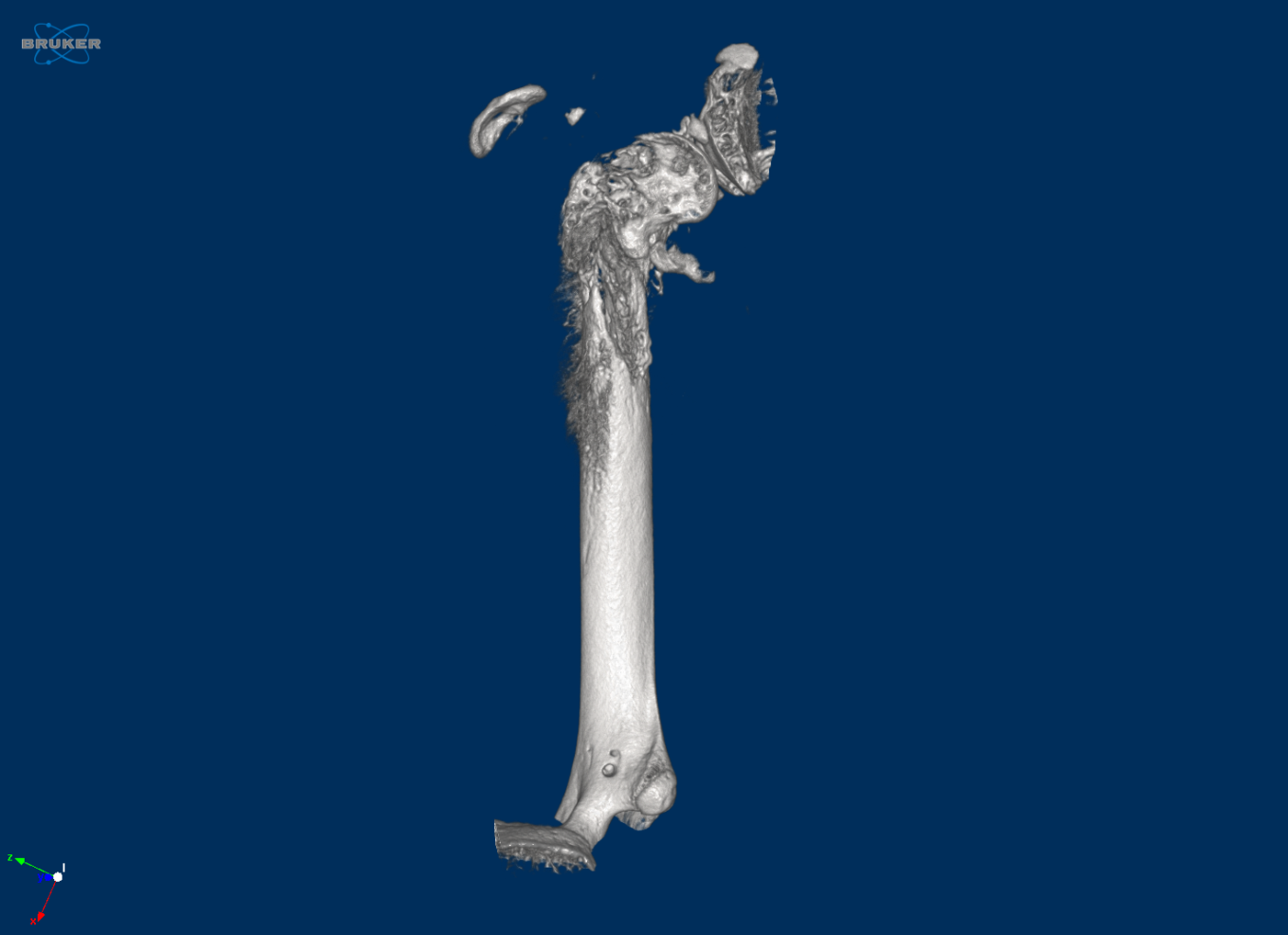

Supplement: Supplementary file 10 — EV and Appendix Figures Source Data [file 44319_2024_292_MOESM10_ESM.zip › EMBOR-2024-59294V3-Figure_S2_Source_Data-sd/Appendix Figure S2/S2B/S2B-2.tif]

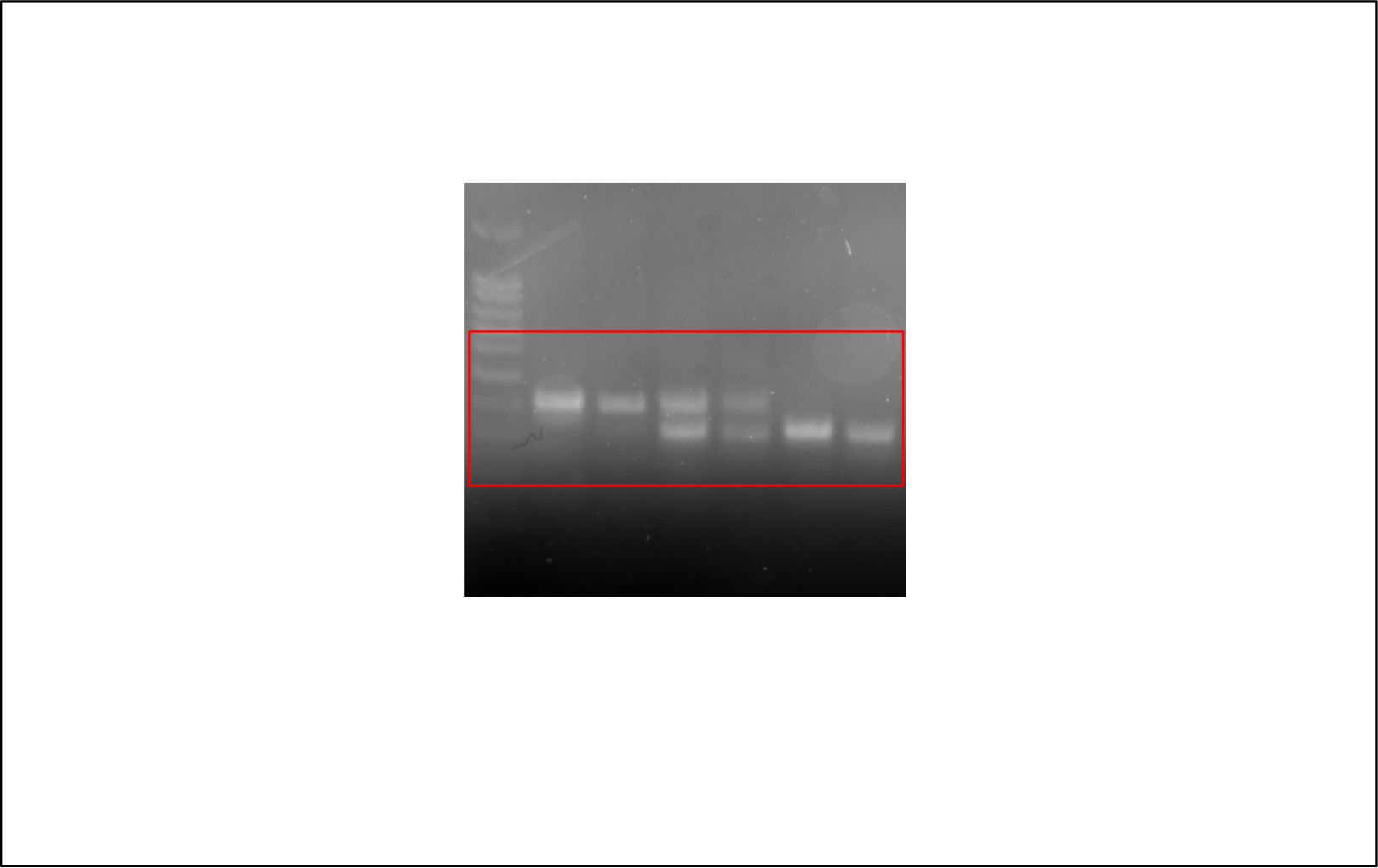

Supplement: Supplementary file 10 — EV and Appendix Figures Source Data [file 44319_2024_292_MOESM10_ESM.zip › EMBOR-2024-59294V3-Figure_S2_Source_Data-sd/Appendix Figure S2/S2C/S2C.tif]

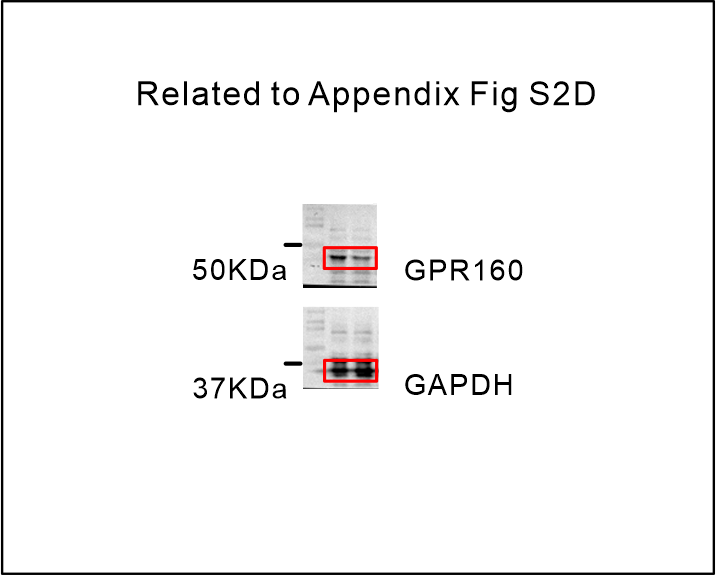

Supplement: Supplementary file 10 — EV and Appendix Figures Source Data [file 44319_2024_292_MOESM10_ESM.zip › EMBOR-2024-59294V3-Figure_S2_Source_Data-sd/Appendix Figure S2/S2D/S2D.tif]

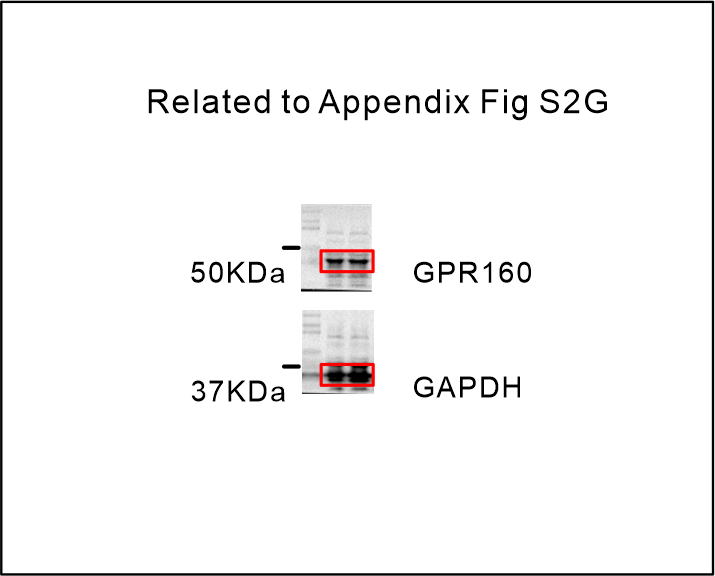

Supplement: Supplementary file 10 — EV and Appendix Figures Source Data [file 44319_2024_292_MOESM10_ESM.zip › EMBOR-2024-59294V3-Figure_S2_Source_Data-sd/Appendix Figure S2/S2G/S2G.tif]

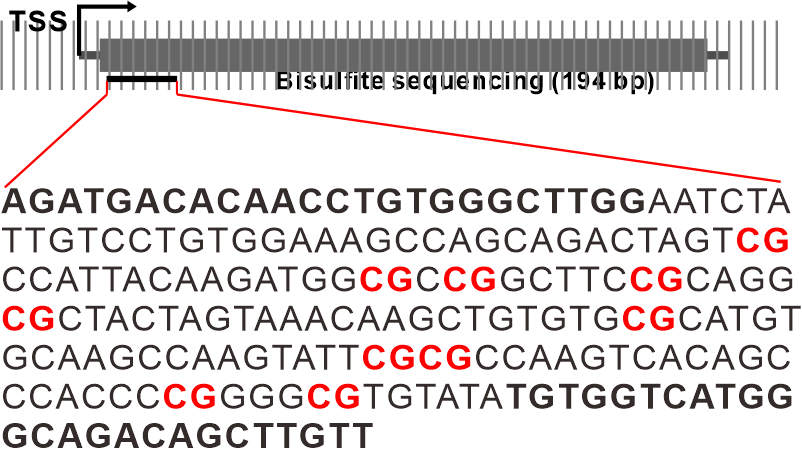

Supplement: Supplementary file 10 — EV and Appendix Figures Source Data [file 44319_2024_292_MOESM10_ESM.zip › EMBOR-2024-59294V3-Figure_S3_Source_Data-sd/Appendix Figure S3/S3A-B/S3A.tif]

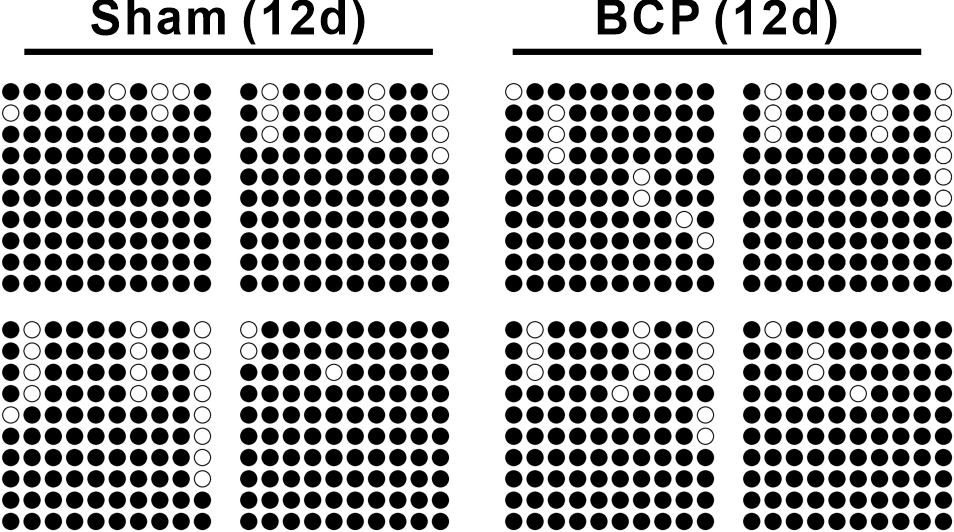

Supplement: Supplementary file 10 — EV and Appendix Figures Source Data [file 44319_2024_292_MOESM10_ESM.zip › EMBOR-2024-59294V3-Figure_S3_Source_Data-sd/Appendix Figure S3/S3A-B/S3B.tif]

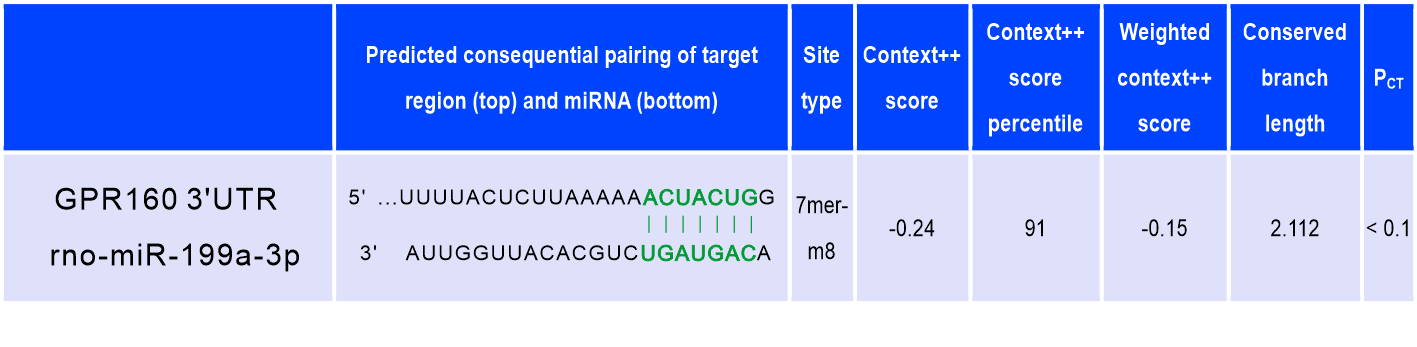

Supplement: Supplementary file 10 — EV and Appendix Figures Source Data [file 44319_2024_292_MOESM10_ESM.zip › EMBOR-2024-59294V3-Figure_S3_Source_Data-sd/Appendix Figure S3/S3D/S3D.tif]

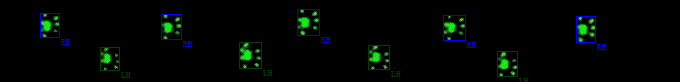

Supplement: Supplementary file 10 — EV and Appendix Figures Source Data [file 44319_2024_292_MOESM10_ESM.zip › EMBOR-2024-59294V3-Figure_EV1_Source_Data-sd/Figure EV1/EV1D/1-1.jpg]

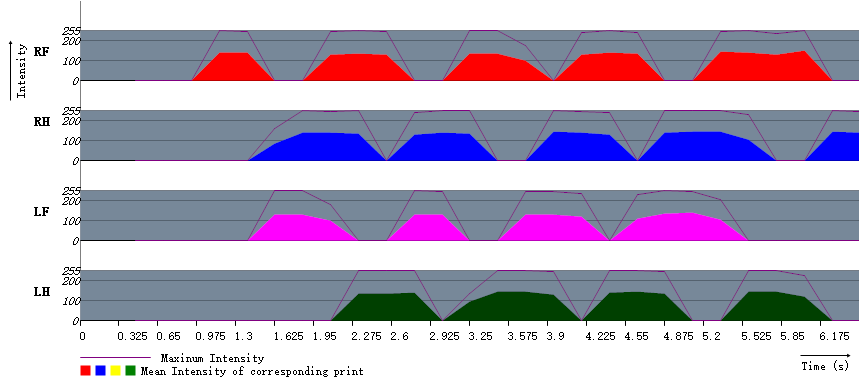

Supplement: Supplementary file 10 — EV and Appendix Figures Source Data [file 44319_2024_292_MOESM10_ESM.zip › EMBOR-2024-59294V3-Figure_EV1_Source_Data-sd/Figure EV1/EV1D/1-2.jpg]

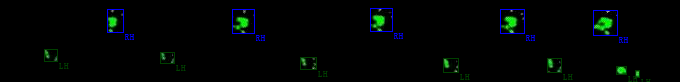

Supplement: Supplementary file 10 — EV and Appendix Figures Source Data [file 44319_2024_292_MOESM10_ESM.zip › EMBOR-2024-59294V3-Figure_EV1_Source_Data-sd/Figure EV1/EV1D/2-1.jpg]

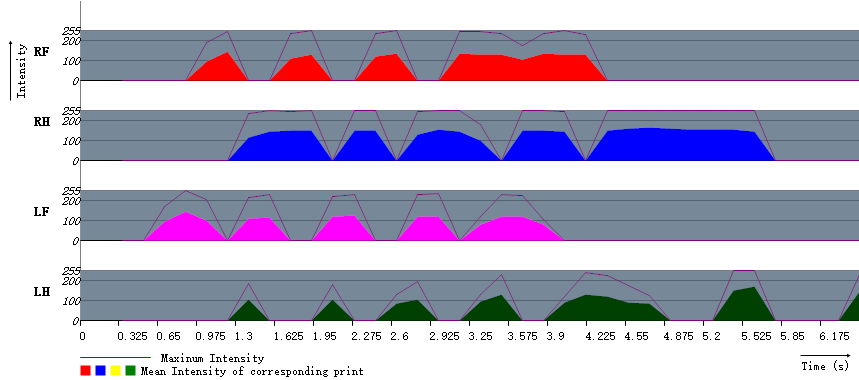

Supplement: Supplementary file 10 — EV and Appendix Figures Source Data [file 44319_2024_292_MOESM10_ESM.zip › EMBOR-2024-59294V3-Figure_EV1_Source_Data-sd/Figure EV1/EV1D/2-2.jpg]

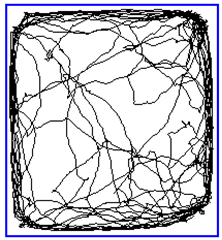

Supplement: Supplementary file 10 — EV and Appendix Figures Source Data [file 44319_2024_292_MOESM10_ESM.zip › EMBOR-2024-59294V3-Figure_EV1_Source_Data-sd/Figure EV1/EV1I/EV1I-1.tif]

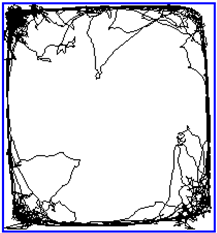

Supplement: Supplementary file 10 — EV and Appendix Figures Source Data [file 44319_2024_292_MOESM10_ESM.zip › EMBOR-2024-59294V3-Figure_EV1_Source_Data-sd/Figure EV1/EV1I/EV1I-2.tif]

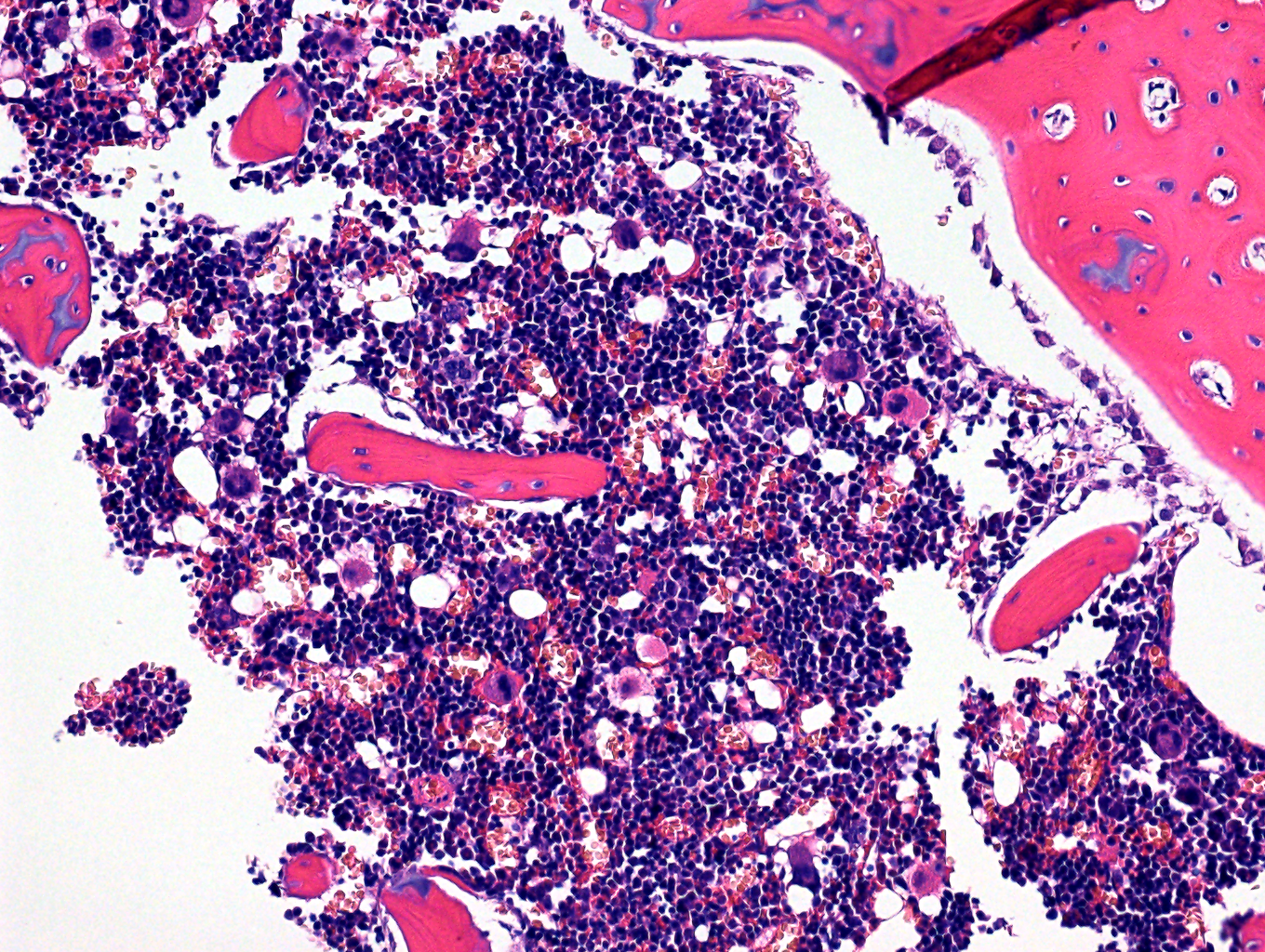

Supplement: Supplementary file 10 — EV and Appendix Figures Source Data [file 44319_2024_292_MOESM10_ESM.zip › EMBOR-2024-59294V3-Figure_EV1_Source_Data-sd/Figure EV1/EV1L/EV1L-1.tif]

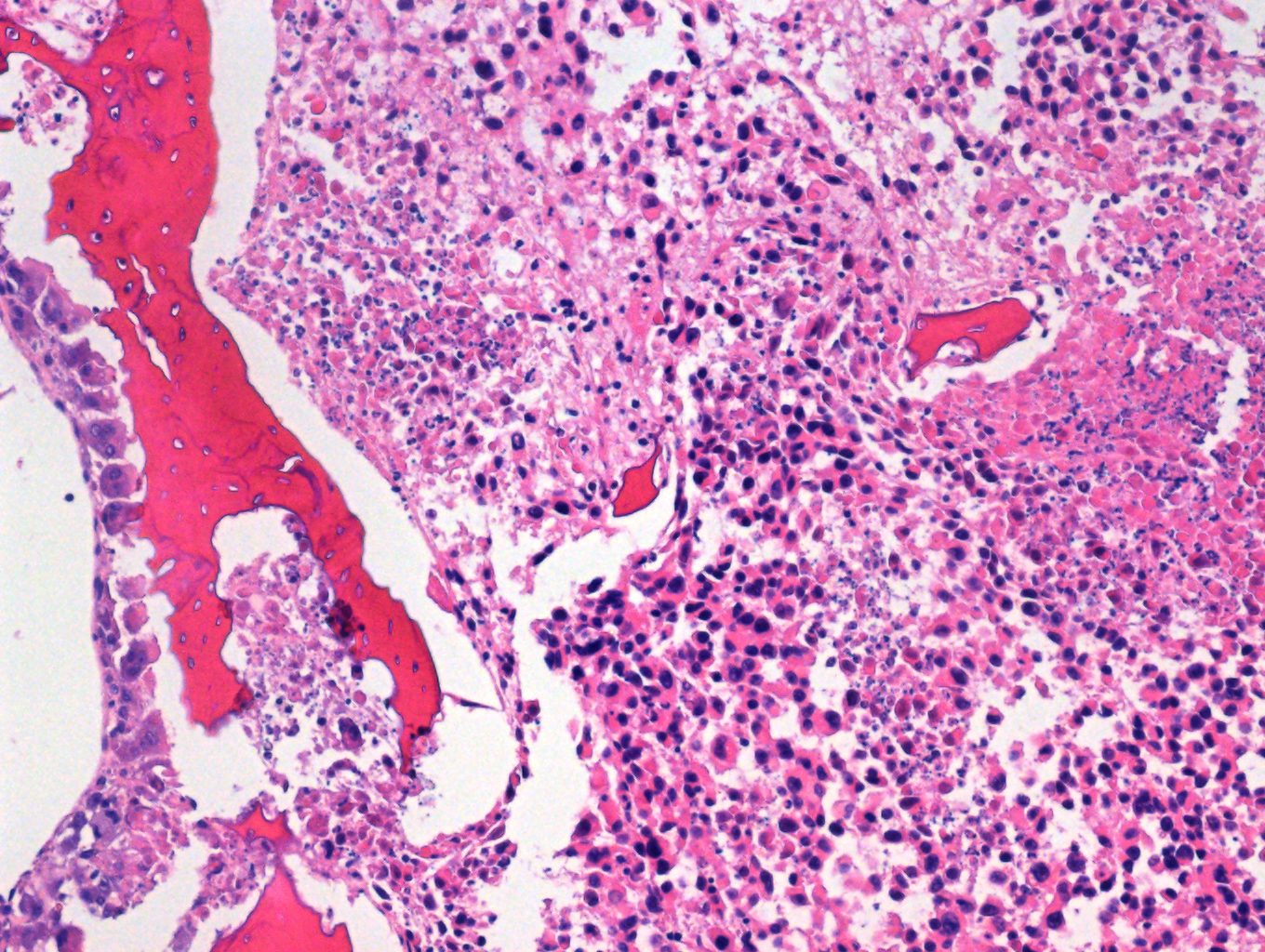

Supplement: Supplementary file 10 — EV and Appendix Figures Source Data [file 44319_2024_292_MOESM10_ESM.zip › EMBOR-2024-59294V3-Figure_EV1_Source_Data-sd/Figure EV1/EV1L/EV1L-2.tif]

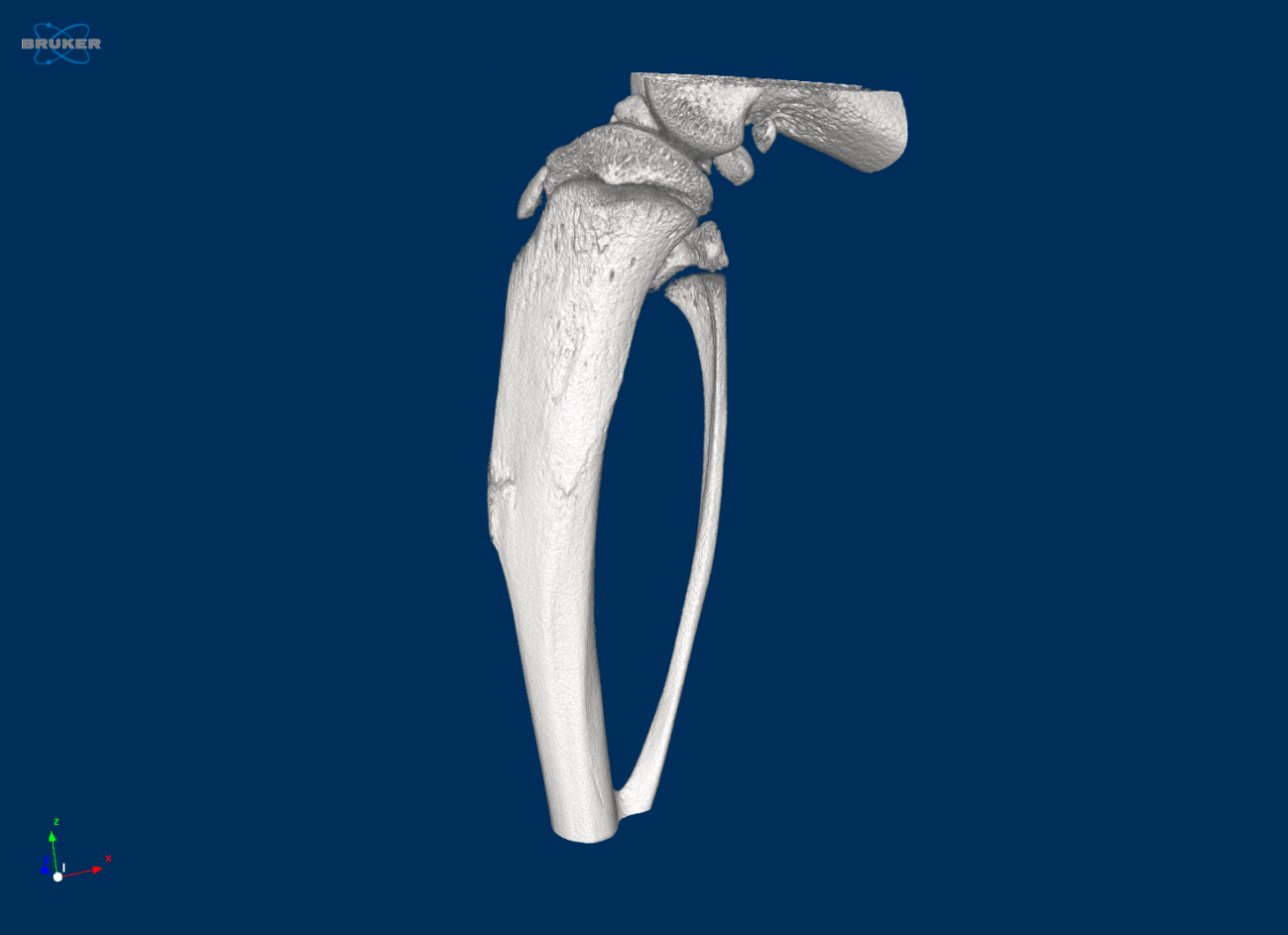

Supplement: Supplementary file 10 — EV and Appendix Figures Source Data [file 44319_2024_292_MOESM10_ESM.zip › EMBOR-2024-59294V3-Figure_EV1_Source_Data-sd/Figure EV1/EV1M/EV1M-1.tif]
