# Supplementary figures and images for: Histone modifications and Sp1 promote GPR160 expression in bone cancer pain within rodent models (part 2 of 2)
Source: EMBO Rep. 2024 Oct 24;25(12):5429–55. doi: 10.1038/s44319-024-00292-6 (PMC11624276; doi:10.1038/s44319-024-00292-6)

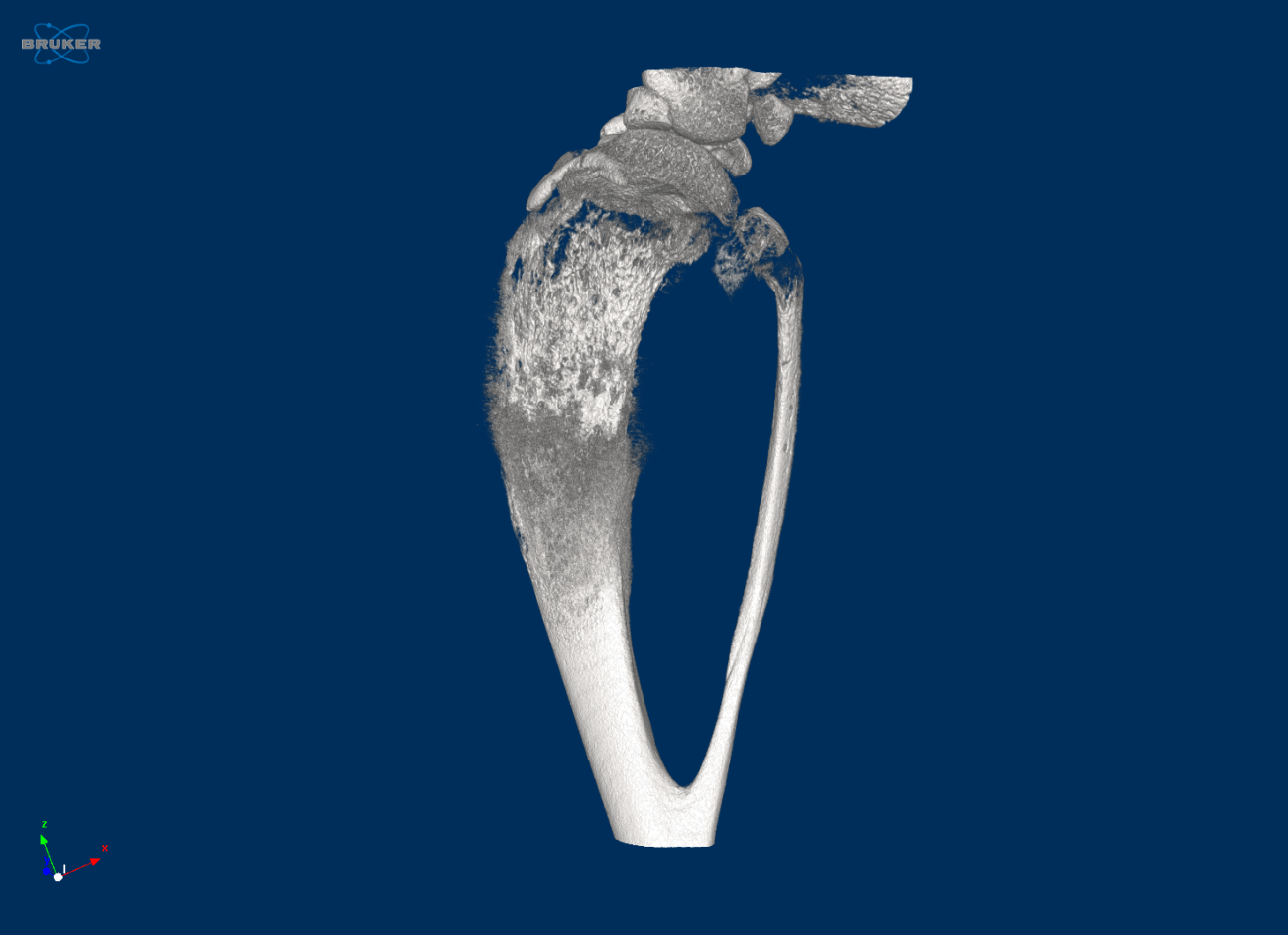

Supplement: Supplementary file 10 — EV and Appendix Figures Source Data [file 44319_2024_292_MOESM10_ESM.zip › EMBOR-2024-59294V3-Figure_EV1_Source_Data-sd/Figure EV1/EV1M/EV1M-2.tif]

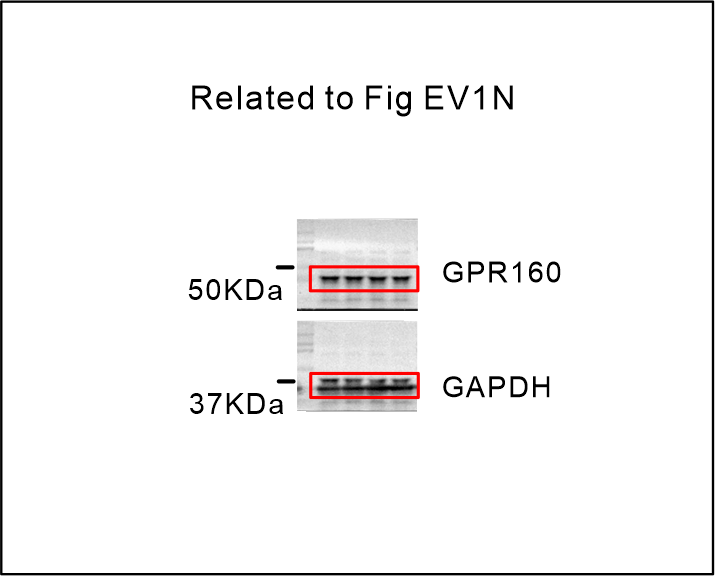

Supplement: Supplementary file 10 — EV and Appendix Figures Source Data [file 44319_2024_292_MOESM10_ESM.zip › EMBOR-2024-59294V3-Figure_EV1_Source_Data-sd/Figure EV1/EV1N/EV1N-1.tif]

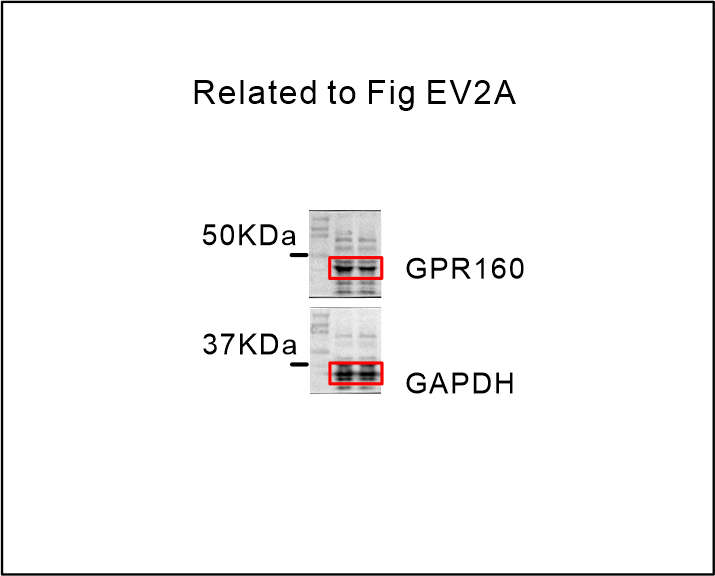

Supplement: Supplementary file 10 — EV and Appendix Figures Source Data [file 44319_2024_292_MOESM10_ESM.zip › EMBOR-2024-59294V3-Figure_EV2_Source_Data-sd/Figure EV2/EV2A/EV2A.tif]

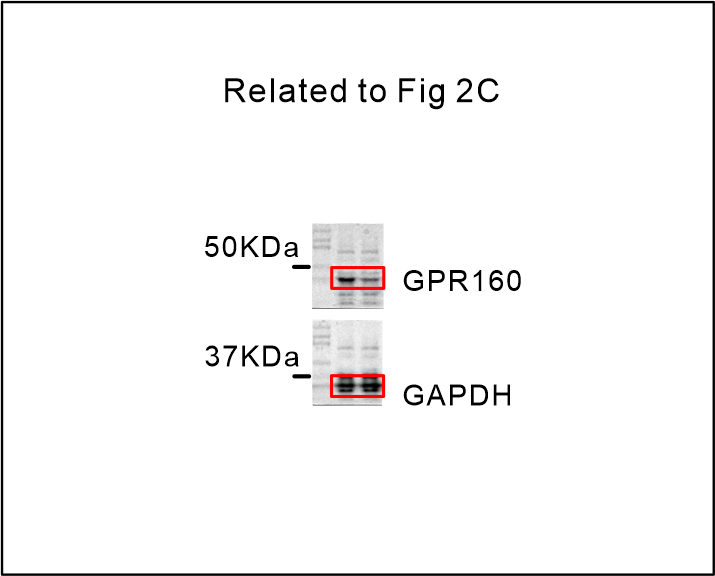

Supplement: Supplementary file 10 — EV and Appendix Figures Source Data [file 44319_2024_292_MOESM10_ESM.zip › EMBOR-2024-59294V3-Figure_EV2_Source_Data-sd/Figure EV2/EV2C/EV2C.tif]

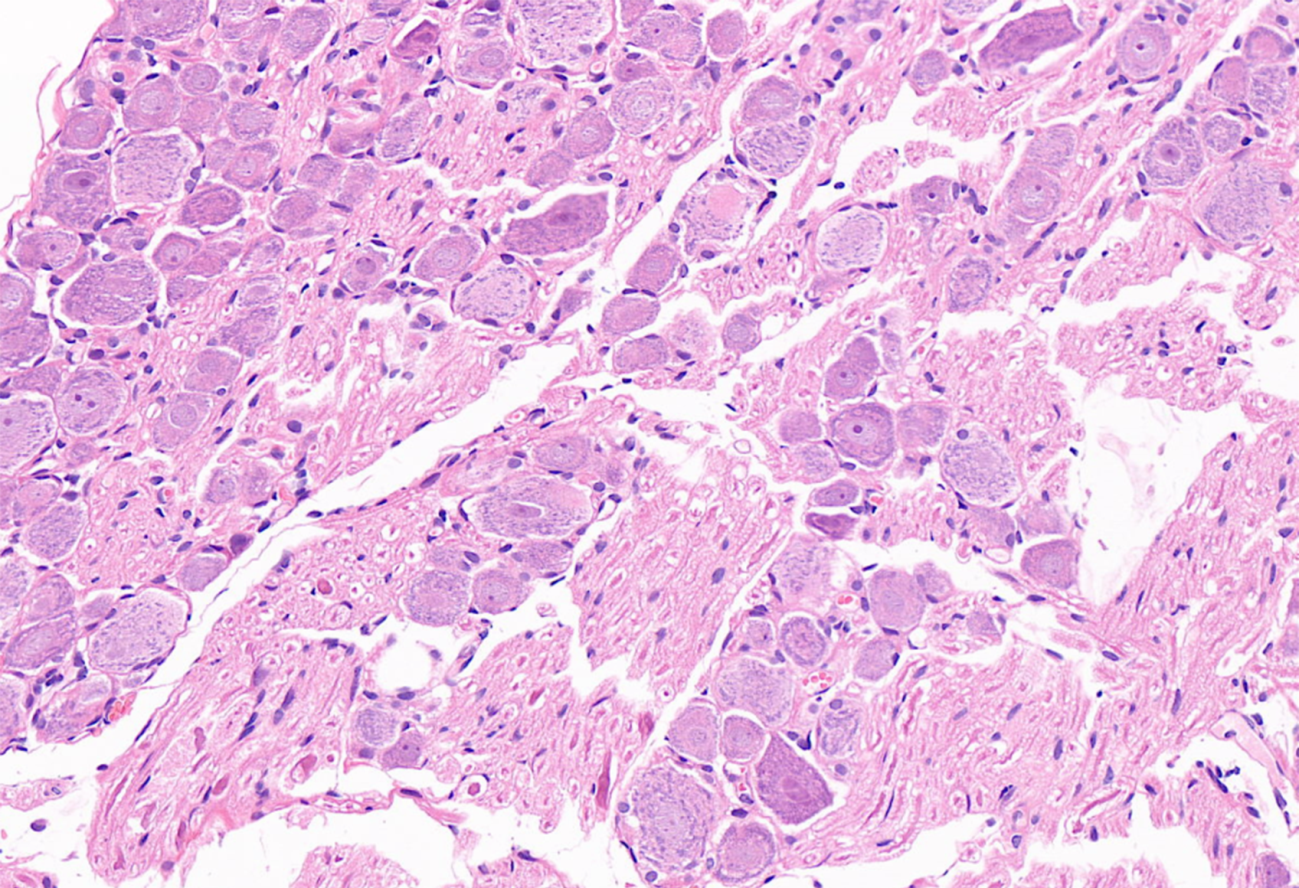

Supplement: Supplementary file 10 — EV and Appendix Figures Source Data [file 44319_2024_292_MOESM10_ESM.zip › EMBOR-2024-59294V3-Figure_EV3_Source_Data-sd/Figure EV3/EV3A/EV3A-1.tif]

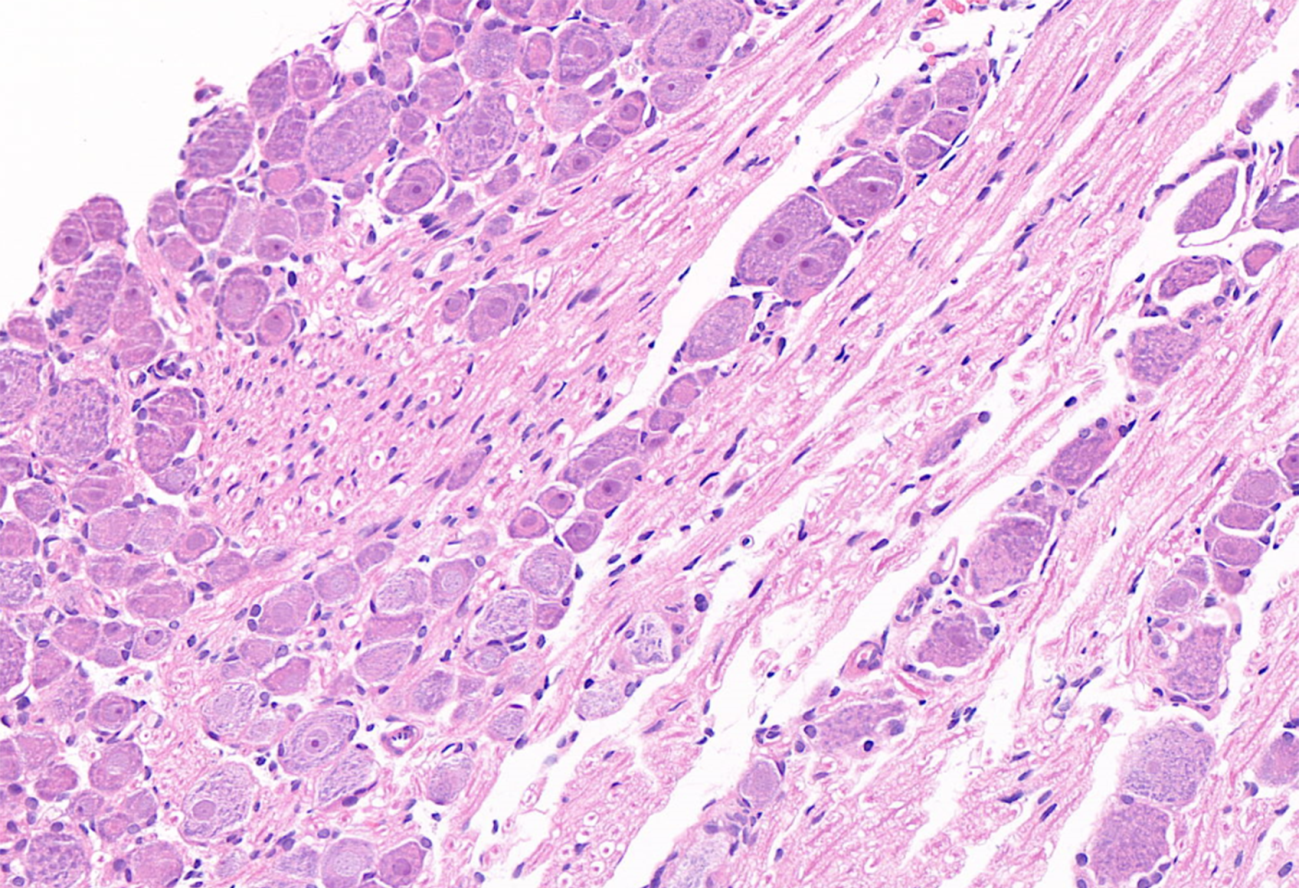

Supplement: Supplementary file 10 — EV and Appendix Figures Source Data [file 44319_2024_292_MOESM10_ESM.zip › EMBOR-2024-59294V3-Figure_EV3_Source_Data-sd/Figure EV3/EV3A/EV3A-2.tif]

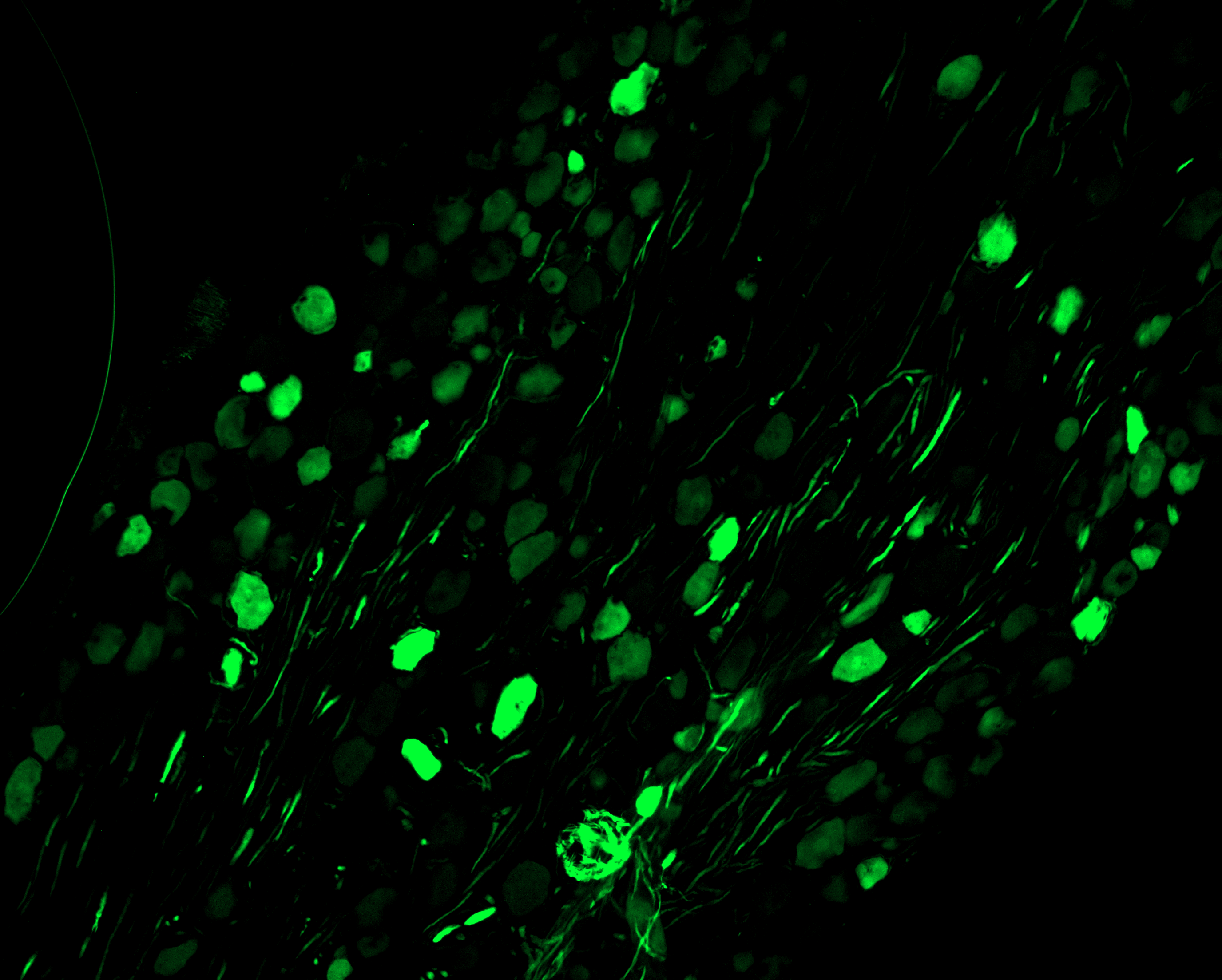

Supplement: Supplementary file 10 — EV and Appendix Figures Source Data [file 44319_2024_292_MOESM10_ESM.zip › EMBOR-2024-59294V3-Figure_EV3_Source_Data-sd/Figure EV3/EV3B/EV3B-1.tif]

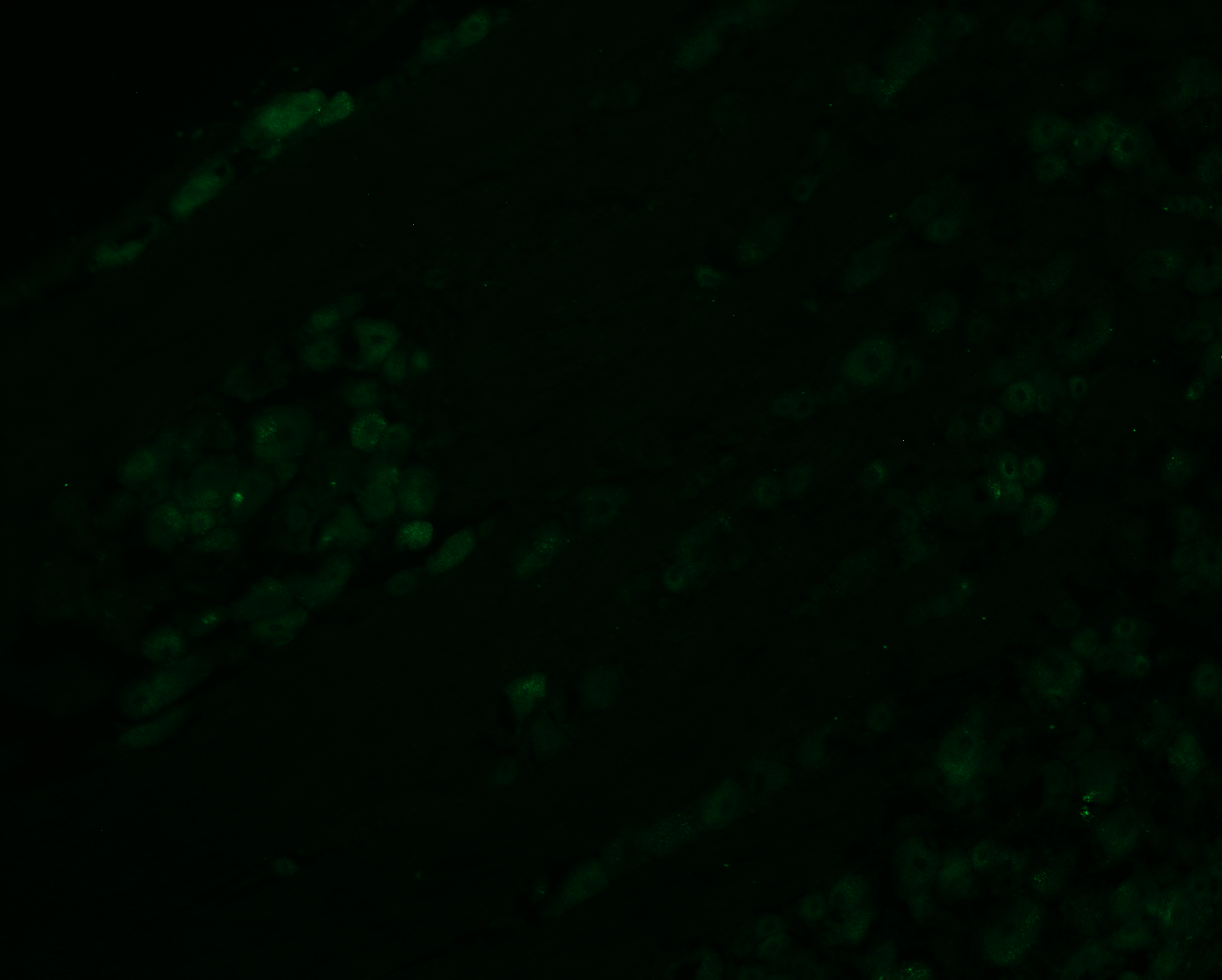

Supplement: Supplementary file 10 — EV and Appendix Figures Source Data [file 44319_2024_292_MOESM10_ESM.zip › EMBOR-2024-59294V3-Figure_EV3_Source_Data-sd/Figure EV3/EV3B/EV3B-2.tif]

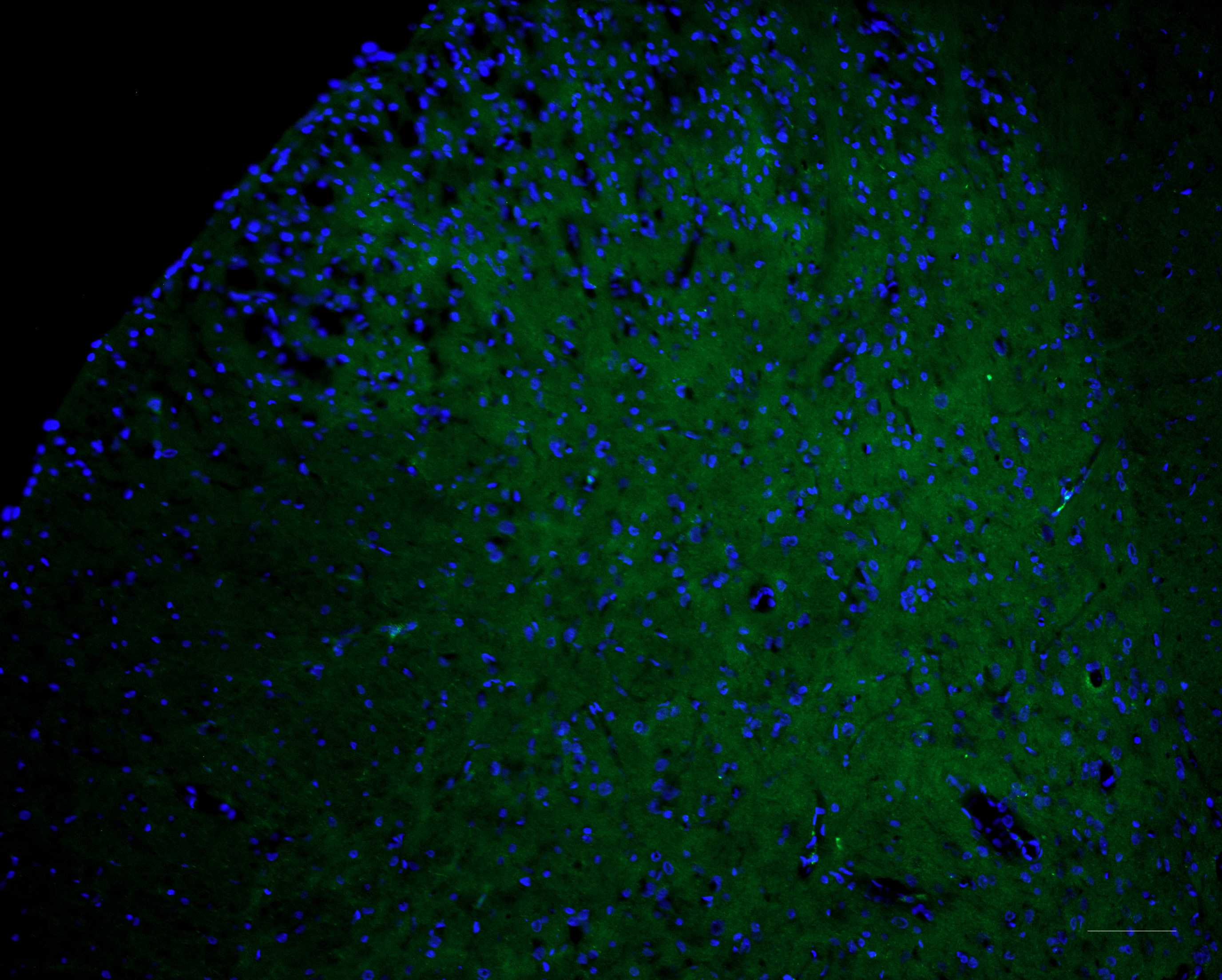

Supplement: Supplementary file 10 — EV and Appendix Figures Source Data [file 44319_2024_292_MOESM10_ESM.zip › EMBOR-2024-59294V3-Figure_EV3_Source_Data-sd/Figure EV3/EV3B/EV3B-3.tif]

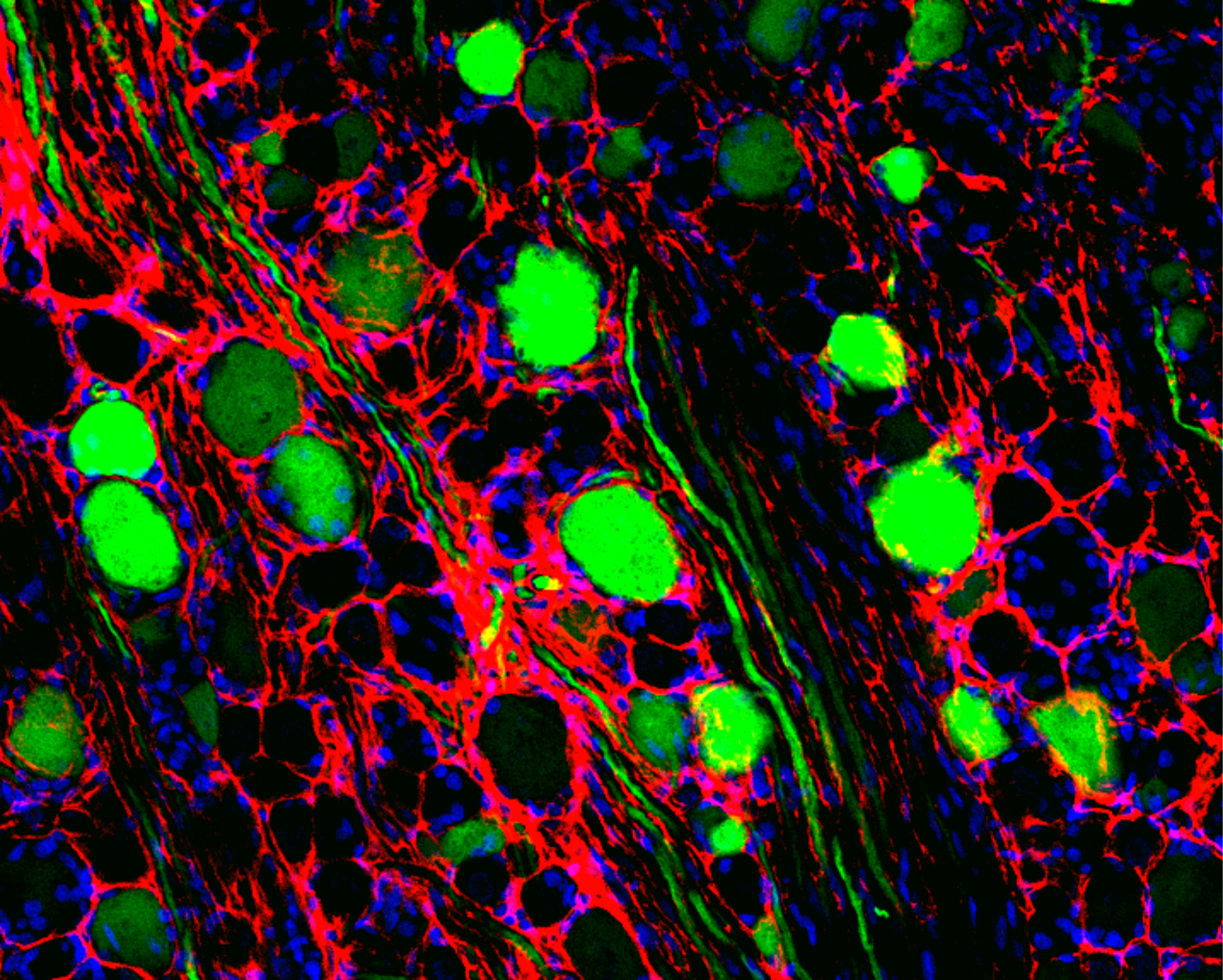

Supplement: Supplementary file 10 — EV and Appendix Figures Source Data [file 44319_2024_292_MOESM10_ESM.zip › EMBOR-2024-59294V3-Figure_EV3_Source_Data-sd/Figure EV3/EV3C/EV3C-1.tif]

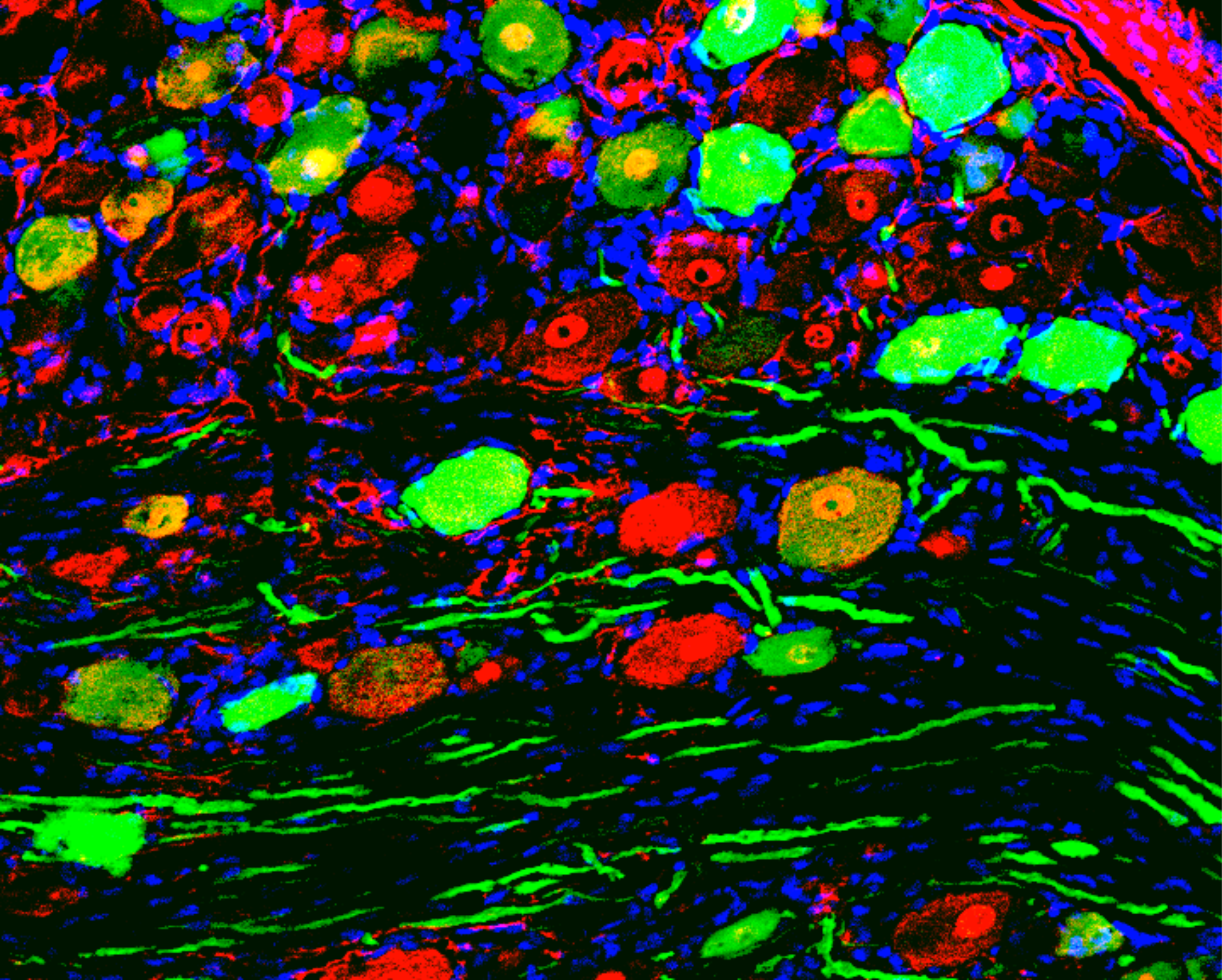

Supplement: Supplementary file 10 — EV and Appendix Figures Source Data [file 44319_2024_292_MOESM10_ESM.zip › EMBOR-2024-59294V3-Figure_EV3_Source_Data-sd/Figure EV3/EV3C/EV3C-2.tif]

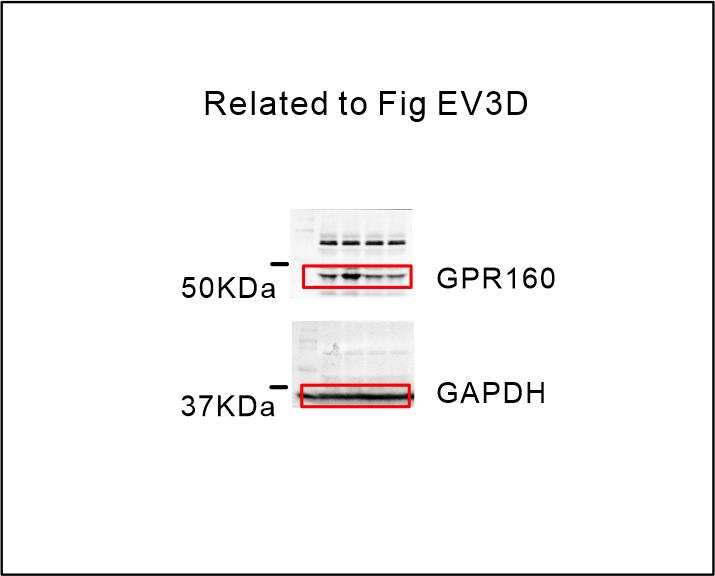

Supplement: Supplementary file 10 — EV and Appendix Figures Source Data [file 44319_2024_292_MOESM10_ESM.zip › EMBOR-2024-59294V3-Figure_EV3_Source_Data-sd/Figure EV3/EV3D/EV3D.tif]

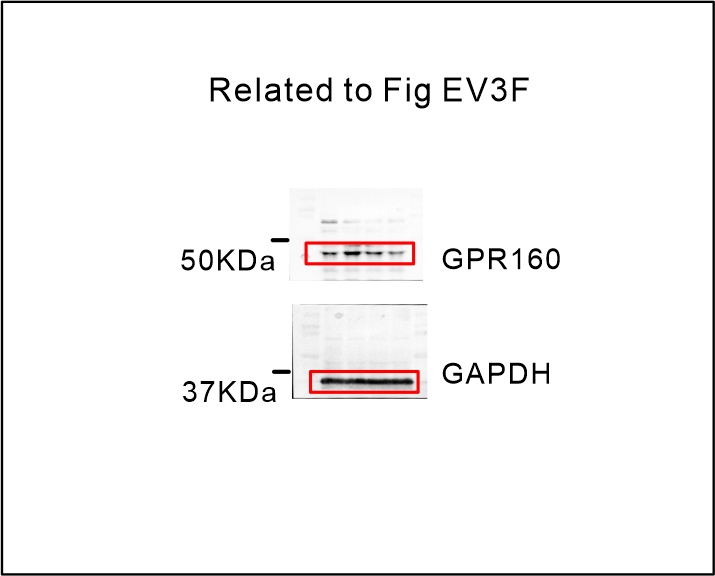

Supplement: Supplementary file 10 — EV and Appendix Figures Source Data [file 44319_2024_292_MOESM10_ESM.zip › EMBOR-2024-59294V3-Figure_EV3_Source_Data-sd/Figure EV3/EV3F/EV3F.tif]

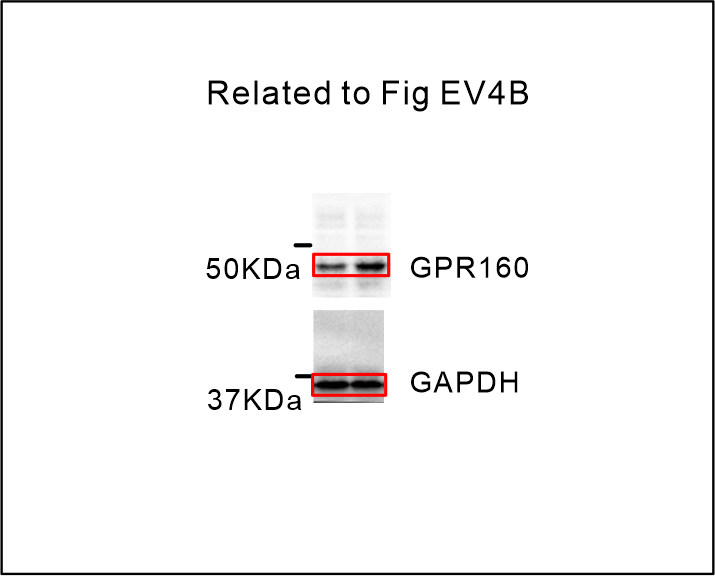

Supplement: Supplementary file 10 — EV and Appendix Figures Source Data [file 44319_2024_292_MOESM10_ESM.zip › EMBOR-2024-59294V3-Figure_EV4_Source_Data-sd/Figure EV4/EV4B/EV4B.tif]

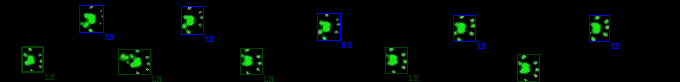

Supplement: Supplementary file 10 — EV and Appendix Figures Source Data [file 44319_2024_292_MOESM10_ESM.zip › EMBOR-2024-59294V3-Figure_EV4_Source_Data-sd/Figure EV4/EV4H/1-1.jpg]

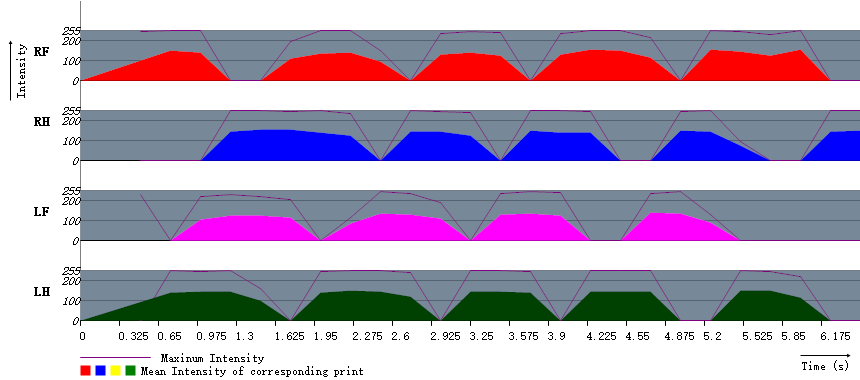

Supplement: Supplementary file 10 — EV and Appendix Figures Source Data [file 44319_2024_292_MOESM10_ESM.zip › EMBOR-2024-59294V3-Figure_EV4_Source_Data-sd/Figure EV4/EV4H/1-2.jpg]

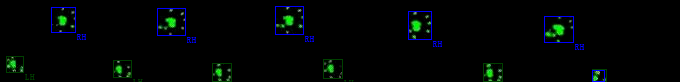

Supplement: Supplementary file 10 — EV and Appendix Figures Source Data [file 44319_2024_292_MOESM10_ESM.zip › EMBOR-2024-59294V3-Figure_EV4_Source_Data-sd/Figure EV4/EV4H/2-1.jpg]

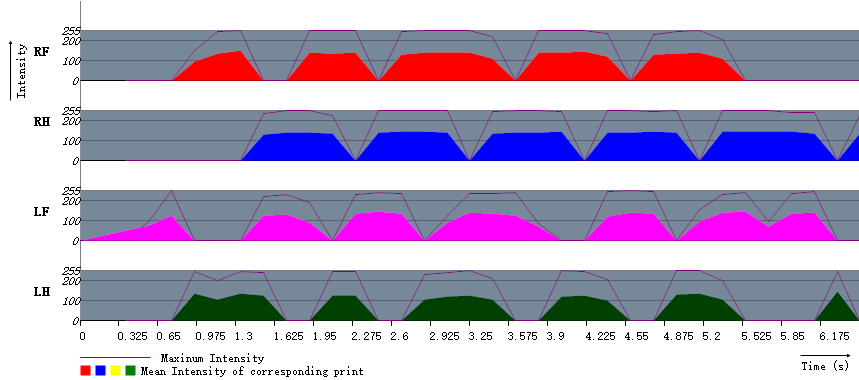

Supplement: Supplementary file 10 — EV and Appendix Figures Source Data [file 44319_2024_292_MOESM10_ESM.zip › EMBOR-2024-59294V3-Figure_EV4_Source_Data-sd/Figure EV4/EV4H/2-2.jpg]

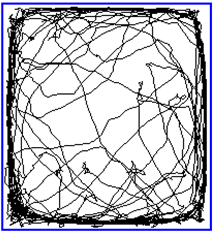

Supplement: Supplementary file 10 — EV and Appendix Figures Source Data [file 44319_2024_292_MOESM10_ESM.zip › EMBOR-2024-59294V3-Figure_EV4_Source_Data-sd/Figure EV4/EV4M/EV4M-1.tif]

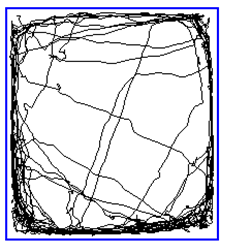

Supplement: Supplementary file 10 — EV and Appendix Figures Source Data [file 44319_2024_292_MOESM10_ESM.zip › EMBOR-2024-59294V3-Figure_EV4_Source_Data-sd/Figure EV4/EV4M/EV4M-2.tif]
