# Supplementary material for: Histone modifications and Sp1 promote GPR160 expression in bone cancer pain within rodent models
Source: EMBO Rep. 2024 Oct 24;25(12):5429–55. doi: 10.1038/s44319-024-00292-6 (PMC11624276; doi:10.1038/s44319-024-00292-6)
Supplement: Supplementary file 11 — Expanded View Figures [file 44319_2024_292_MOESM11_ESM.pdf]

Expanded View Figures

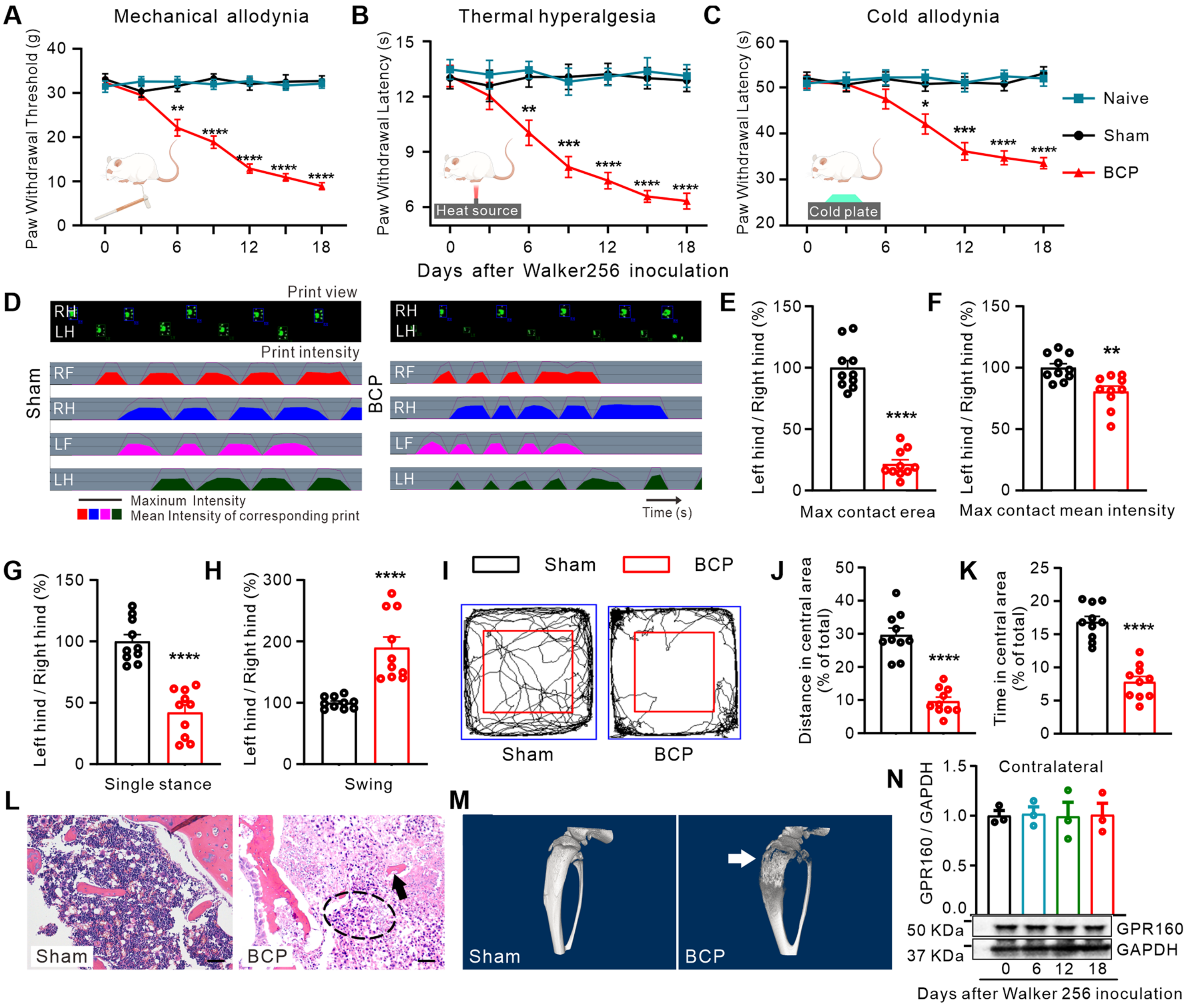

Figure EV1. Tumor infiltration to establish a bone cancer pain model induces pain-related behaviors in rats.

(**A–C**) Paw withdrawal threshold (PWT) to von Frey filament stimuli (**A**, $^{**}P = 0.0019$, $^{****}P < 0.0001$ versus sham group) as well as paw withdrawal latencies (PWLs) to thermal (**B**, $^{**}P = 0.0081$, $^{***}P = 0.0001$, $^{****}P < 0.0001$ versus sham group) and cold (**C**, $^{*}P = 0.0127$, $^{****}P < 0.0001$ versus sham group) stimuli on the ipsilateral (Ipsi) at various time points (days 0, 3, 6, 9, 12, 15 and 18) after BCP or sham surgery. Data are mean ± SEM of biological replicates $n = 10$ rats/group, two-way ANOVA with repeated measures followed by the post hoc Tukey test. (**D**) Representative CatWalk gait, including print view and print intensity, for sham and BCP group. (**E–H**) Tumor infiltration resulted in a reduction of maximum contact area (**E**, $^{****}P < 0.0001$ versus sham group), maximum contact mean intensity (**F**, $^{**}P = 0.0024$ versus sham group), and single stance duration (**G**, $^{****}P < 0.0001$ versus sham group), along with an increase in swing duration (**H**, $^{****}P < 0.0001$ versus sham group) among tumor-bearing rats. LH, left hind; RH, right hind. Student's unpaired *t* test, Data are mean ± SEM of biological replicates $n = 10$ rats. (**I**) Representative rodent tracks recorded from rats 12 days post-surgery during the open field test. (**J, K**) Center distance (**J**, $^{****}P < 0.0001$ versus sham group) and time (**K**, $^{****}P < 0.0001$ versus sham group) for rats 12 days post-surgery in the open field test. Student's unpaired *t* test, Data are mean ± SEM of biological replicates $n = 10$ rats. (**L**) Hematoxylin and eosin (H&E) staining revealed the infiltration of tumor cells, accompanied by medullary bone loss and tibial bone destruction, on POD 18. Scale bar: 50 µm. (**M**) Representative micro-CT images showing tibia bone microstructure in sham and BCP rats on POD 18. (**N**) Representative immunoblots and summarized data showing the GPR160 protein expression in the contralateral L4/5 DRG on days 0, 6, 12, and 18 post-BCP. One-way ANOVA with repeated measures followed by post hoc Tukey test, Data are mean ± SEM of biological replicates $n = 3$ rats/group, $P = 0.9993$ versus sham group. Source data are available online for this figure.

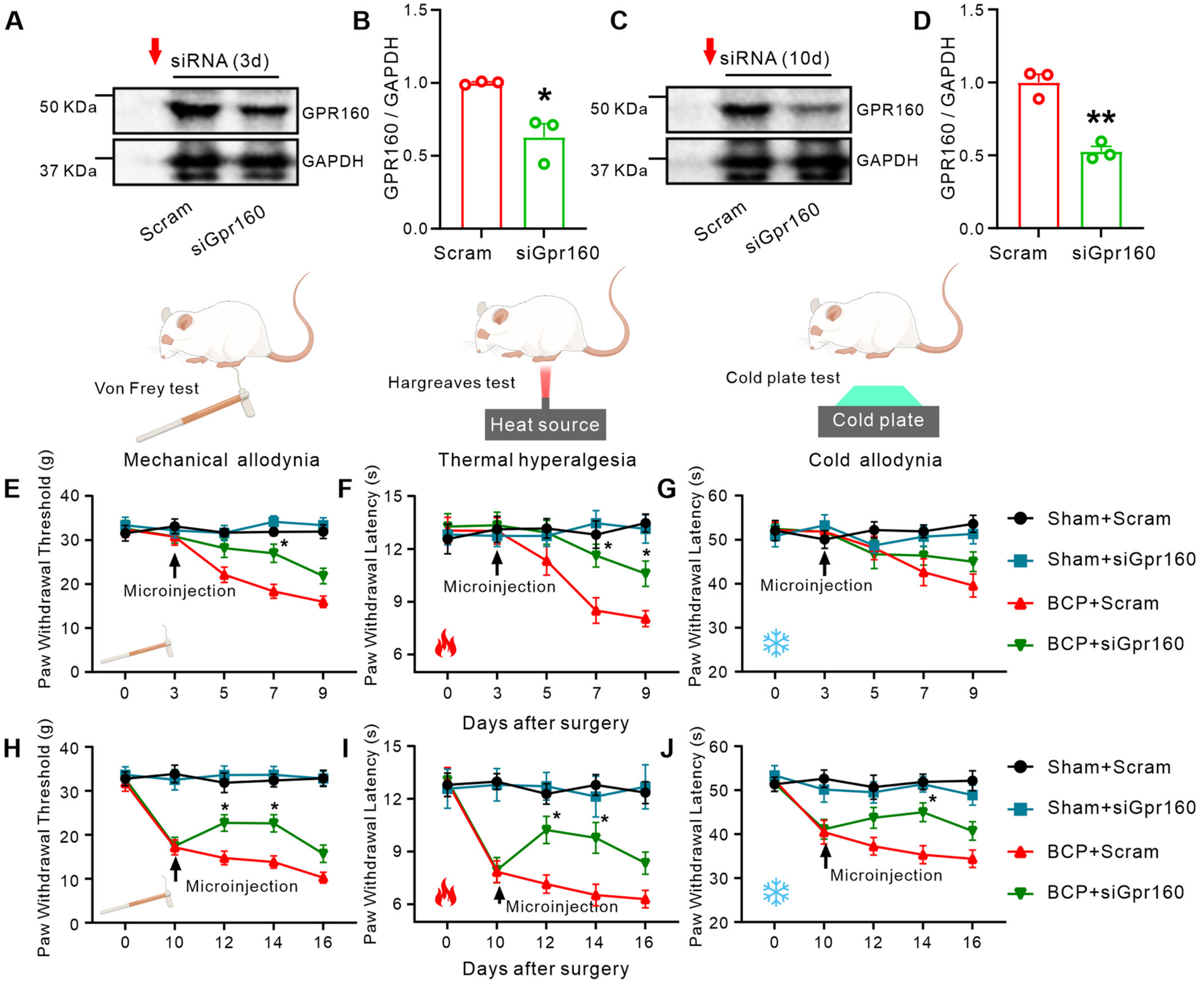

Figure EV2. DRG increased GPR160 is required for development and maintenance of BCP in rats.

(**A–D**) Effect of microinjection of *Gpr160* siRNA (*siGpr160*) or scramble siRNA (Scram) into the ipsilateral L4/5 DRG in rats on the expression of GPR160 on POD 7 (**B**, *$P = 0.0195$ versus BCP plus Scram group) and POD 14 (**D**, **$P = 0.0016$ versus BCP plus Scram group). Student's unpaired *t* test. Data are mean ± SEM of biological replicates $n = 3$ rats/group. The red arrow represents microinjection. (**E–G**) Effect of pre-microinjection of *siGpr160* or Scram into the ipsilateral L4/5 DRG of rats on the development of BCP-induced mechanical allodynia (**E**, *$P = 0.0251$ versus BCP plus Scram group) and heat hyperalgesia (**F**, *$P = 0.0317$, *$P = 0.0496$ versus BCP plus Scram group), with no discernible effect on cold allodynia (**G**, $P = 0.4244$ versus BCP plus Scram group) on the ipsilateral side. Data are mean ± SEM of biological replicates $n = 7$–8 rats/group. Two-way ANOVA with repeated measures followed by post hoc Tukey test. (**H–J**) Effect of siRNA microinjection of siGpr160 or Scram into the ipsilateral L4/5 DRG of rats on the persistence of BCP-induced mechanical allodynia (**H**, *$P = 0.0302$, *$P = 0.0171$ versus BCP plus Scram group), heat hyperalgesia (**I**, *$P = 0.0302$, *$P = 0.0472$ versus BCP plus Scram group) and cold allodynia (**J**, *$P = 0.0282$ versus BCP plus Scram group) on the ipsilateral side. Data are mean ± SEM of biological replicates $n = 7$–8 rats/group. Two-way ANOVA with repeated measures followed by post hoc Tukey test. Source data are available online for this figure.

 © The Author(s)

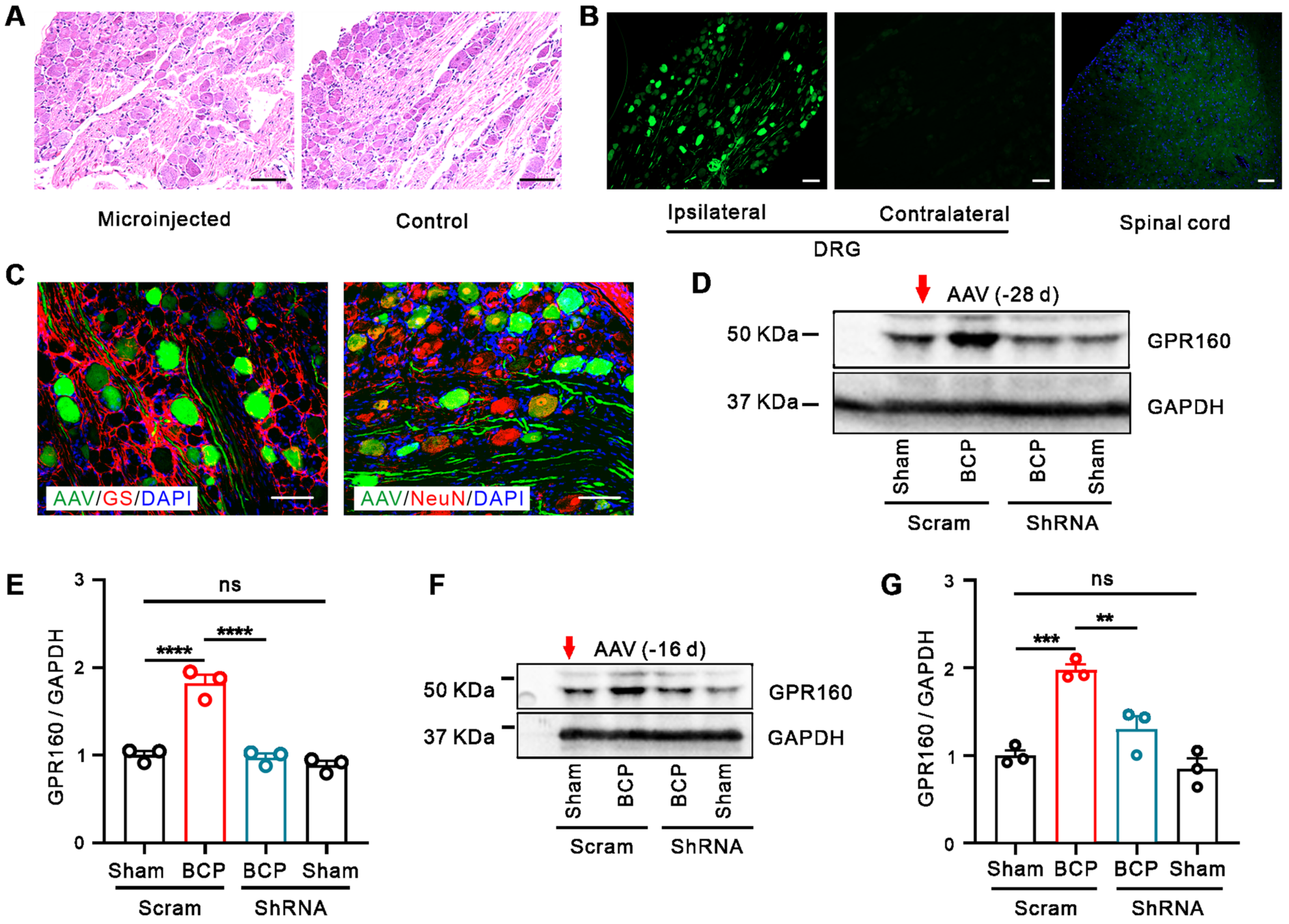

Figure EV3. Validation of targeted gene delivery and expression.

(**A**) Representative hematoxylin and eosin-stained images of control un-injected and injected DRGs, captured 28 days post-microinjection. Scale bar: 100 µm. (**B**) Representative images displaying GFP expression in the ipsilateral and contralateral L4/5 DRG and spinal cord at 28 days post-microinjection with AAV-*Gpr160* (*Gpr160*) or AAV-*Gfp* (*Gfp*) into the ipsilateral L4/5 DRG. $n = 3$ rats. Scale bar: 100 µm. (**C**) Representative images of co-localization of GFP and GS, or NeuN in the AAV-injected DRG. $n = 3$ rats. Scale bar: 100 µm. (**D–G**) Representative immunoblots (**D**, **F**) and summarized data (**E**, $^{****}P < 0.0001$ versus the BCP plus Scram group, $P = 0.8416$ versus the Sham plus Scram group and **G**, $^{***}P = 0.0008$, $^{**}P = 0.0085$ versus the BCP plus Scram group, $P = 0.6486$ versus the Sham plus Scram group) illustrating the GPR160 protein expression on POD 18 in the ipsilateral L4/5 DRG pre-microinjected with AAV-*Gpr160* (*Gpr160*) or control AAV-*Gfp* (*Gfp*) 28 days (**D**, **E**) and 16 days (**F**, **G**) prior to surgery. Two-way ANOVA with repeated measures followed by post hoc Tukey test, Data are mean ± SEM of biological replicates $n = 3$ rats/group. The red arrow represents microinjection. Source data are available online for this figure.

© The Author(s)

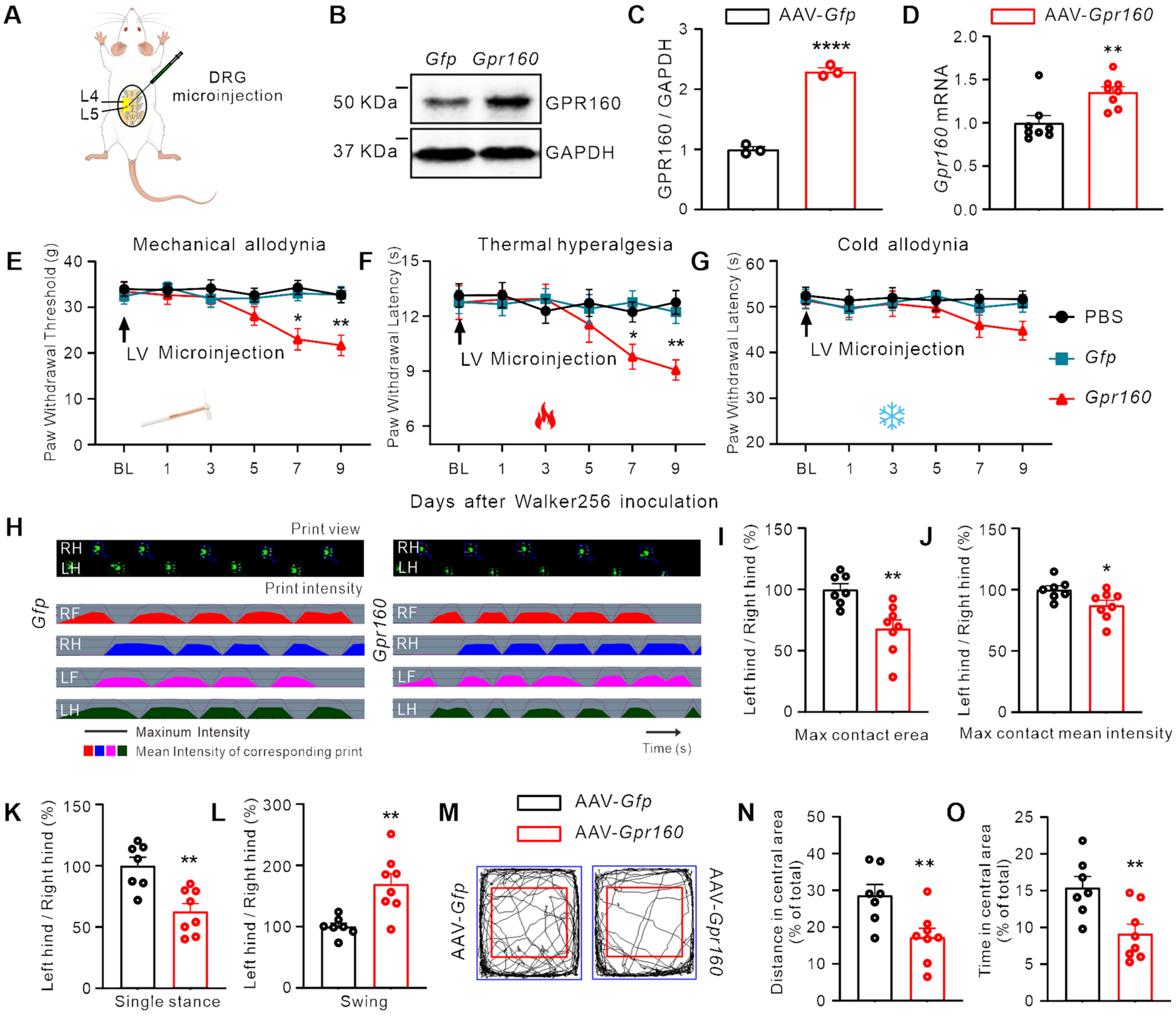

Figure EV4. Effect of DRG GPR160 overexpression on nociceptive thresholds and pain-related behavior in naive female rats.

(A) Schematic protocol for DRG microinjection in rat. **(B, C)** Representative immunoblots and summarized data showing GPR160 protein expression in the ipsilateral L4/5 DRG of rats at 5 weeks post-microinjection with AAV-*Gpr160* (*Gpr160*) or AAV-*Gfp* (*Gfp*). Student's unpaired t test, Data are mean ± SEM of biological replicates $n = 3$ rats/group, $^{****}P < 0.0001$ versus AAV-*Gfp* group. **(D)** The *Gpr160* mRNA expression in the ipsilateral DRG of rats at 5 weeks post-microinjection with AAV-*Gpr160* or AAV-*Gfp*. Student's unpaired *t* test, Data are mean ± SEM of biological replicates $n = 8$ rats/group, $^{**}P = 0.0041$ versus AAV-*Gfp* group. **(E–G)** Paw withdrawal responses to mechanical (**E**, $^{*}P = 0.0114$, $^{**}P = 0.0058$ versus the *Gfp* group), heat (**F**, $^{*}P = 0.0193$, $^{**}P = 0.0055$ versus the *Gfp* group), and cold (**G**, $P = 0.1229$ versus the *Gfp* group) stimuli on the ipsilateral side, recorded at specified time points following microinjection of LV-*Gpr160* (*Gpr160*), LV-*Gfp* (*Gfp*), or PBS into unilateral L4/5 DRGs in naive rats. Data are mean ± SEM of biological replicates $n = 7$–8 rats/group. Two-way ANOVA with repeated measures followed by post hoc Tukey test. **(H)** Representative CatWalk gait, including print view and print intensity, within the DRG microinjection groups treated with AAV-*Gfp* or AAV-*Gpr160*. **(I–L)** DRG microinjection of AAV resulted in a reduction of max contact area (**I**, $^{**}P = 0.0032$ versus AAV-*Gfp* group), max contact mean intensity (**J**, $^{*}P = 0.0309$ versus AAV-*Gfp* group) and single stance (**K**, $^{**}P = 0.0017$ versus AAV-*Gfp* group), along with an increase in swing duration (**L**, $^{**}P = 0.0027$ versus AAV-*Gfp* group) in naive female rats. Student's unpaired t test, Data are mean ± SEM of biological replicates $n = 7$–8 rats/group. **(M)** Representative animal tracks observed in naive rats following AAV microinjection during the open field test. **(N, O)** Center time (**N**, $^{**}P = 0.0071$ versus AAV-*Gfp* group) and distance (**O**, $^{**}P = 0.0098$ versus AAV-*Gfp* group) measurements for rats 5 weeks post-AAV microinjection during the open field test. Student's unpaired *t* test, Data are mean ± SEM of biological replicates $n = 7$–8 rats/group. Source data are available online for this figure.

 © The Author(s)

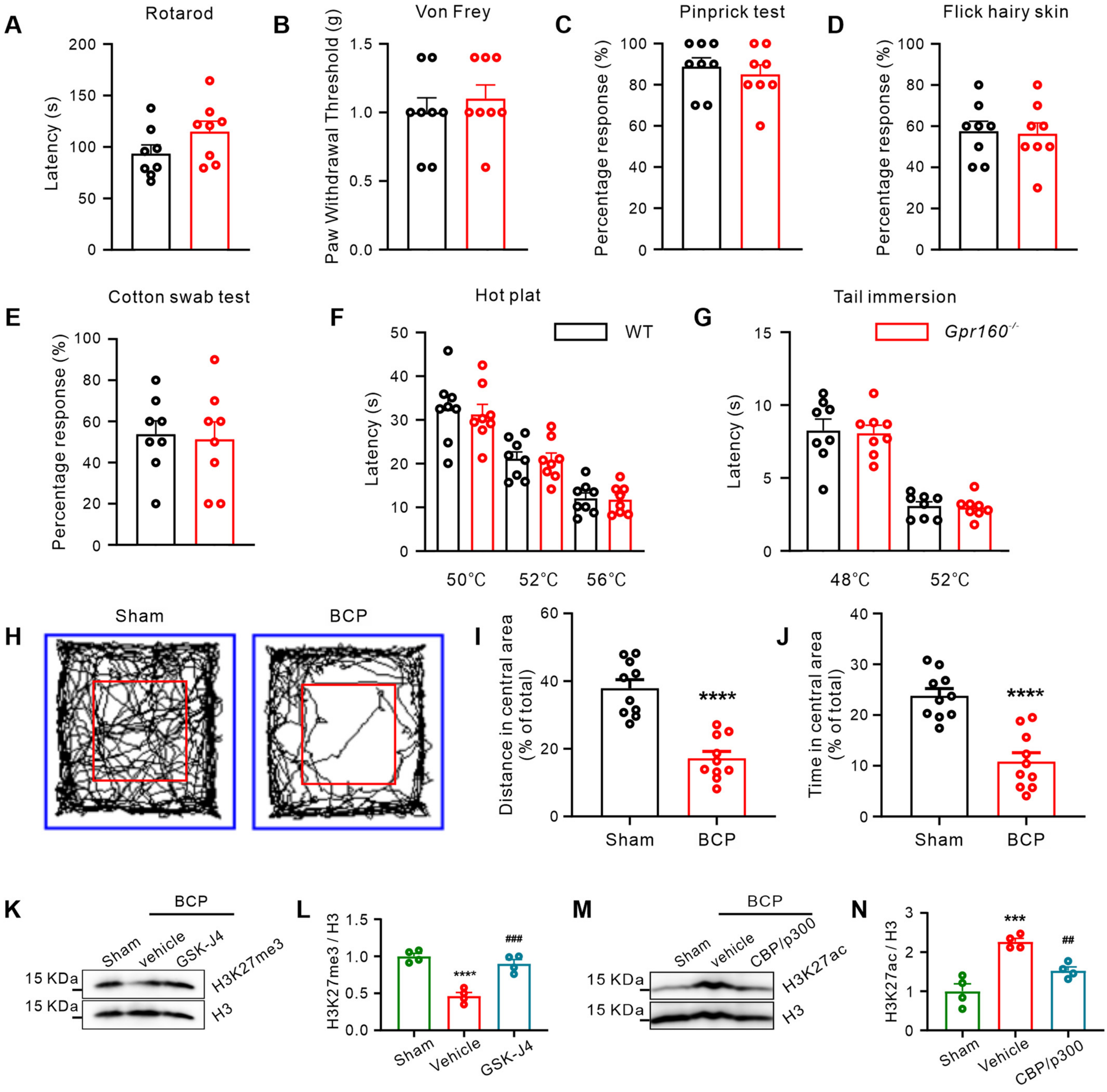

Figure EV5. Basal motor and sensory function in WT and *Gpr160*$^{-/-}$ mice, and establishment of the mouse bone cancer pain model.

(**A**) Motor function was tested through Rotarod assay. Student's unpaired *t* test, Data are mean ± SEM of biological replicates n = 8 mice/group, $P = 0.1301$ versus WT group. (**B**, **C**) Mechanical pain sensation was measured through von Frey test (**B**, $P = 0.5057$ versus WT group) and pinprick test (**C**, $P = 0.5667$ versus WT group). Student's unpaired *t* test, Data are mean ± SEM of biological replicates $n = 8$ mice/group. (**D**, **E**) Evaluation of light-touch sensation was conducted via the flick hairy skin test (**D**, $P = 0.8654$ versus WT group) and cotton swab test (**E**, $P = 0.8195$ versus WT group). Student's unpaired *t* test, Data are mean ± SEM of biological replicates n = 8 mice/group. (**F**, **G**) Thermal sensation was gauged using the hot plate assay (**F**, $P = 0.7378$, $P = 0.8807$, $P = 0.8572$ versus WT group) and tail immersion assay (**G**, $P = 0.8463$, $P = 0.8006$ versus WT group). Student's unpaired *t* test, Data are mean ± SEM of biological replicates $n = 8$ mice/group. (**H**) Representative animal tracks were recorded from mice 18 d after surgery in the open field test. (**I**, **J**) Measurements of distance (**I**, ****$P < 0.0001$ versus WT group) and time (**J**, ****$P < 0.0001$ versus WT group) in the center were obtained for mice 12 d post-surgery in the open field test. Student's unpaired *t* test, Data are mean ± SEM of biological replicates $n = 10$ mice/group. (**K**, **L**) Representative immunoblots (**K**) and summarized data (**L**) showing the decreased protein expression level of H3K27me3 in DRG on POD 15 after BCP was intensified by GSK-J4. One-way ANOVA with repeated measures followed by post hoc Tukey test, Data are mean ± SEM of biological replicates $n = 4$ rats/group, ****$P < 0.0001$ versus Sham group. ###$P = 0.0004$ versus BCP plus vehicle group. (**M**, **N**) Representative immunoblots (**M**) and summarized data (**N**) showing the increased protein expression level of H3K27ac in DRG on POD 15 after BCP was attenuated by CBP/p300. One-way ANOVA with repeated measures followed by post hoc Tukey test, Data are mean ± SEM of biological replicates $n = 4$ rats/group, ***$P = 0.0002$ versus Sham group. ##$P = 0.0091$ versus BCP plus vehicle group. Source data are available online for this figure.
